# A systematic evaluation of the performance and properties of the UK Biobank Polygenic Risk Score (PRS) Release

**DOI:** 10.1371/journal.pone.0307270

**Published:** 2024-09-18

**Authors:** Deborah J. Thompson, Daniel Wells, Saskia Selzam, Iliana Peneva, Rachel Moore, Kevin Sharp, William A. Tarran, Edward J. Beard, Fernando Riveros-Mckay, Carla Giner-Delgado, Duncan Palmer, Priyanka Seth, James Harrison, Marta Futema, Genomics England Research Consortium, Gil McVean, Vincent Plagnol, Peter Donnelly, Michael E. Weale

**Affiliations:** 1 Genomics plc, Oxford, United Kingdom; 2 Cardiology Research Centre, Molecular and Clinical Sciences Research Institute, St George’s University of London, London, United Kingdom; 3 Institute of Cardiovascular Science, Faculty of Population Health Sciences, University College London, London, United Kingdom; Penn State: The Pennsylvania State University, UNITED STATES OF AMERICA

## Abstract

We assess the UK Biobank (UKB) Polygenic Risk Score (PRS) Release, a set of PRSs for 28 diseases and 25 quantitative traits that has been made available on the individuals in UKB, using a unified pipeline for PRS evaluation. We also release a benchmarking software tool to enable like-for-like performance evaluation for different PRSs for the same disease or trait. Extensive benchmarking shows the PRSs in the UKB Release to outperform a broad set of 76 published PRSs. For many of the diseases and traits we also validate the PRS algorithms in a separate cohort (100,000 Genomes Project). The availability of PRSs for 53 traits on the same set of individuals also allows a systematic assessment of their properties, and the increased power of these PRSs increases the evidence for their potential clinical benefit.

## Introduction

Polygenic risk scores (PRSs) provide a personalised measure of genetic liability of disease, combining genetic risk information from across the genome [1, 2]. PRSs can also be used to measure the genetic contribution to quantitative traits (for simplicity, we also use the term PRS here for such traits). The field is growing rapidly, with advances in methods [3], tools [4], reporting standards [5], and cataloguing [6–8]. There is also mounting evidence for their clinical utility [9]. For example, a PRS algorithm for coronary artery disease has similar predictive power to LDL cholesterol [10], an established clinical risk factor, while PRS algorithms for coronary artery disease [11] and for breast cancer [12] have been shown to identify groups with equivalent risk to rare monogenic variant carriers. The extensive interest in PRSs means there are a large number now available; in some cases more than 100 PRS algorithms have been published for the same disease [7].

Prospectively collected biobanks such as UK Biobank [13] (UKB) play an important role in enabling PRS development, evaluation, and application [14]. They can provide data for PRS training, but more importantly they provide large representative population samples for evaluation of PRSs in multiple contexts, including ancestry (herein, ‘ancestry’ refers to genetically inferred ancestry, see S1 File). They also provide a broad base of other clinical and biomolecular information, both to train and evaluate multi-factor clinical risk models and for other research applications [15–19].

To enable PRS research and development, we have previously made available the UK Biobank PRS Release (Category 300, https://biobank.ndph.ox.ac.uk/ukb/label.cgi?id=300), which comprises well-powered PRSs for 28 diseases and 25 quantitative traits, together with associated data [20]. The UK Biobank PRS Release comprises two PRS sets, a Standard Set with scores calculated on all individuals in UKB, trained on external data only, and an Enhanced Set calculated on a subset of individuals in UKB, which has the advantage of being trained on external data plus additional training data from a separate subgroup of UKB. The Release covers a representative sample of common chronic diseases and disease-relevant quantitative traits, spanning a range of genetic architectures, and for which sufficient genomewide training data were available.

Since its release in mid-2022, the UK Biobank PRS Release has been widely used in research, with over 60 publications to date taking advantage of the resource. In this paper we provide an evaluation of the resource. Our evaluation of the UK Biobank PRS Release falls into several parts. First, we consider predictive performance, comparing the Standard and Enhanced PRS sets to each other and to 76 PRSs from published algorithms, with favourable results. We take care to separate results according to genetic ancestry, as this is a known driver of performance [21, 22]. Next, we consider the interplay between predictive performance and potential for clinical utility. In line with earlier results [11, 12, 23–25], we show that risk profiles of individuals with appropriately high PRS scores are similar to those seen in carriers of known rare pathogenic variants, that these high-PRS individuals account for a much higher fraction of disease, and of early onset disease, and that the PRS score modulates the effect of rare pathogenic variants. Next, we quantify the impact of age and sex on predictive PRS performance, and also describe some multivariate PRS properties and model their influence on mortality. Finally, we provide a separate evaluation of predictive performance in an external dataset, the 100,000 Genomes Project maintained by Genomics England [26, 27]. The polygenic risk scores used in this external validation have also been released for use in the Genomics England research environment.

A level playing field is essential for fair comparisons and evaluations of PRS performance [28]. Reported performance can be influenced by many factors, including the choice of performance metric, covariate adjustment, demographic and study properties of the evaluation cohort, and decisions on how the phenotype was defined [14]. These choices can confound inferences about the performance of the underlying PRS algorithm or PRS methodologies. To address these issues, we have built a unified pipeline for PRS evaluation, constructing a standardised Testing Subgroup within UKB and a standardised set of disease and quantitative trait definitions, and we have used this pipeline to benchmark the UK Biobank PRS Release. We have made this pipeline available as an open source tool within the UK Biobank Research Access Platform, along with the associated phenotype definitions, to allow other researchers to check reported metrics and perform evaluations of their own or others’ PRSs against the UK Biobank Release.

For clarity, in what follows we distinguish three closely related concepts. Throughout, we use: (1) *PRS score*, or just PRS, for the score assigned to a particular individual; (2) *PRS algorithm* for the function which calculates the PRS score from genetic data on an individual; and (3) *PRS methodology* for the approach used to determine a particular PRS algorithm.

## Materials and methods

This is a PRS risk model evaluation study, as per established reporting standards [5]. See S1 File for a point-by-point discussion regarding PRS-RS reporting standards. To ensure uniformity of evaluation using the same individuals, the performance of both Standard and Enhanced PRS sets was evaluated on a standard array of metrics in a single UKB Testing Subgroup. The UKB Testing Subgroup comprised 97,608, 9,542, 9,476 and 2,864 individuals with predominantly European, South Asian, African, and East Asian genetic ancestries, respectively, and was designed to maximise the representation of non-European ancestries for testing and to be independent of other individuals in the UK Biobank (see S1 File). The Standard PRS Set (S1 Table) consists of scores for 28 diseases and 11 quantitative traits, calculated on all UKB individuals, using non-UKB GWAS datasets. The Enhanced PRS Set (S1 Table), consists of scores for 28 diseases and an expanded list of 25 quantitative traits, calculated on UKB individuals not used for training [20]. The phenotype definitions for these traits, as used for evaluating the PRS Sets in the UKB Testing Subgroup, are described in S1 Table.

**Fig 1:**
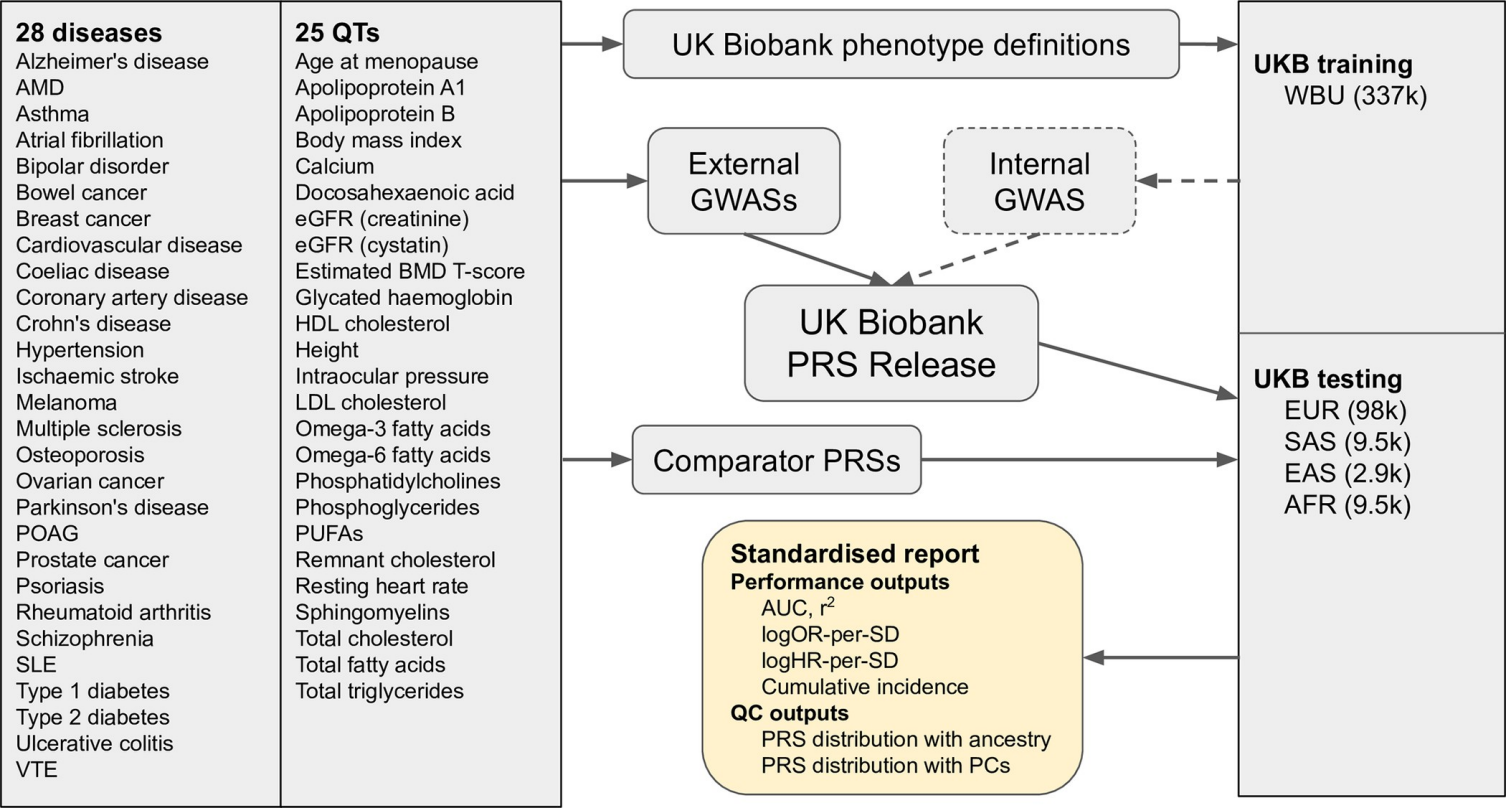
Schematic workflow of the standardised evaluation pipeline for the UK Biobank PRS Release. The UK Biobank PRS Release comprises a Standard PRS set, trained on external GWASs only, and an Enhanced PRS set, trained on both external and internal GWASs, targeting 28 disease and 25 quantitative traits. The evaluation pipeline generates a standardised report for a PRS (either from the UK Biobank PRS Release or from a comparator) across five genetic ancestry groups in a separate UK Biobank testing subgroup. The standardised report includes information on cumulative incidence stratified by PRS; performance metrics including AUC, logHR-per-SD and logOR-per-SD for disease traits and r^2^ for quantitative traits; and PRS distribution metrics. QT = quantitative trait. AMD = age-related macular degeneration. POAG = primary open angle glaucoma. SLE = systemic lupus erythematosus. VTE = venous thromboembolic disease. eGFR = estimated glomerular filtration rate. BMD = bone mineral density. HDL/LDL = high/low density lipoprotein. PUFAs = polyunsaturated fatty acids. UKB = UK Biobank. WBU = white British unrelated. GWAS = genomewide association study. PRS = polygenic risk score. AUC = area under the receiver operating characteristic curve. logOR-per-SD/logHR-per-SD = log odds/hazard ratio per standard deviation of PRS. EUR = European ancestry. SAS = South Asian ancestry. EAS = East Asian ancestry. AFR = African (Sub-Saharan) ancestry. Throughout, ovarian cancer refers specifically to epithelial ovarian cancer.

We built a pipeline for evaluating the performance and properties of the UK Biobank PRS Release in the Testing Subgroup, which is available as a command line tool within the UK Biobank Research Access Platform and as source code (https://github.com/Genomicsplc/ukb-pret) (Fig 1). In order to provide reassurance against UKB-specificity and overfitting, we also carried out performance evaluations in the 100,000 Genomes Project (S2 Table). Our evaluation methods are further described in S1 File.

At the time of recruitment, all UK Biobank participants were provided with an information leaflet and were given the opportunity to ask questions about the project. They consented to the statement “I give permission for access to my medical and other health-related records, and for long-term storage and use of this and other information about me, for health-related research purposes (even after my incapacity or death)” by tapping “I agree” on a touch-screen monitor, followed by a recording of their signature using a stylus on an electronic signature pad. A recruitment staff member determined whether the participant had the mental capacity to provide informed consent, and participants were told they could withdraw their consent at any time (see https://www.ukbiobank.ac.uk/explore-your-participation/basis-of-your-participation). Our research project (project application number 9659) was approved by the UK Biobank according to their established access procedures [29], and legal and ethical approval is covered by the Research Tissue Bank approval obtained from the UK Biobank’s governing Research Ethics Committee (REC 16/NW/0274), as recommended by the National Research Ethics Service.

## Results

### Performance of the PRS Release in UK Biobank

**Fig 2:**
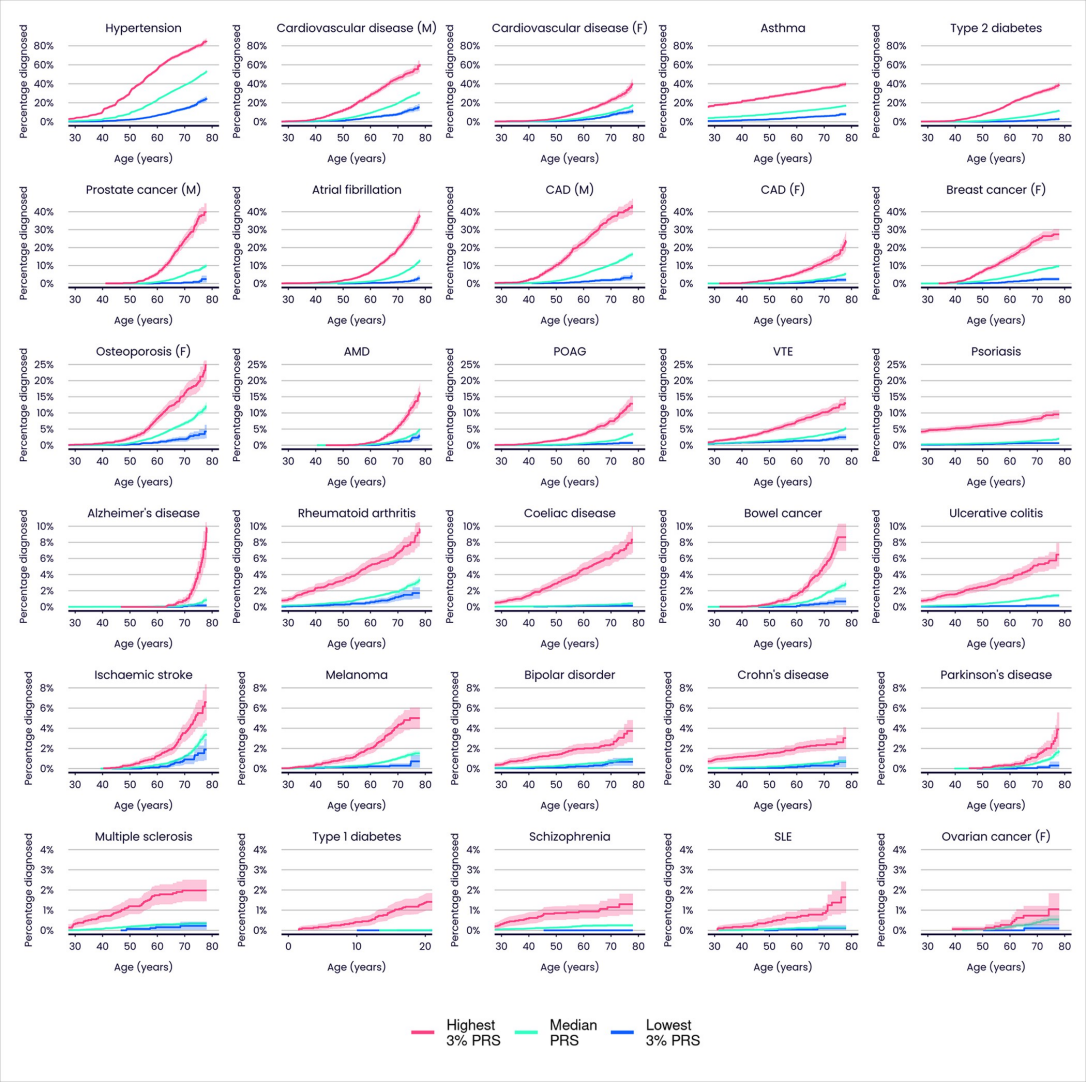
Cumulative incidence plots illustrating the predictive performance of the UK Biobank PRS Release for 28 diseases in individuals with European ancestries (Enhanced PRS Set). Each plot shows the estimated percentage of individuals diagnosed with the stated disease by a given age, for three groups within the UKB Testing Subgroup defined only by their PRS scores. Colours indicate individuals in the highest 3% (red), median 40–60% (green) and lowest 3% (blue) of the Enhanced PRS distribution. M = male, F = female. Shadings indicate 95% confidence intervals. Type 1 diabetes age range is restricted to 0–20 years. CAD = coronary artery disease. Refer to Fig 1 legend for other disease abbreviations.

PRS performance has been quantified in multiple ways. Cumulative incidence plots (Fig 2) provide a useful visual tool for comparing disease incidence over time among individuals grouped according to their PRS for that disease. Notwithstanding known issues in UKB healthy bias [30] and underreporting of some diseases (e.g. type 2 diabetes is reported mainly from primary care records, which are only available for ~40% of UKB participants), Fig 2 reveals large differences in disease incidence across ages for groups defined by the Enhanced PRS Set, further emphasising the potential of PRSs for powerful individual risk stratification.

**Fig 3:**
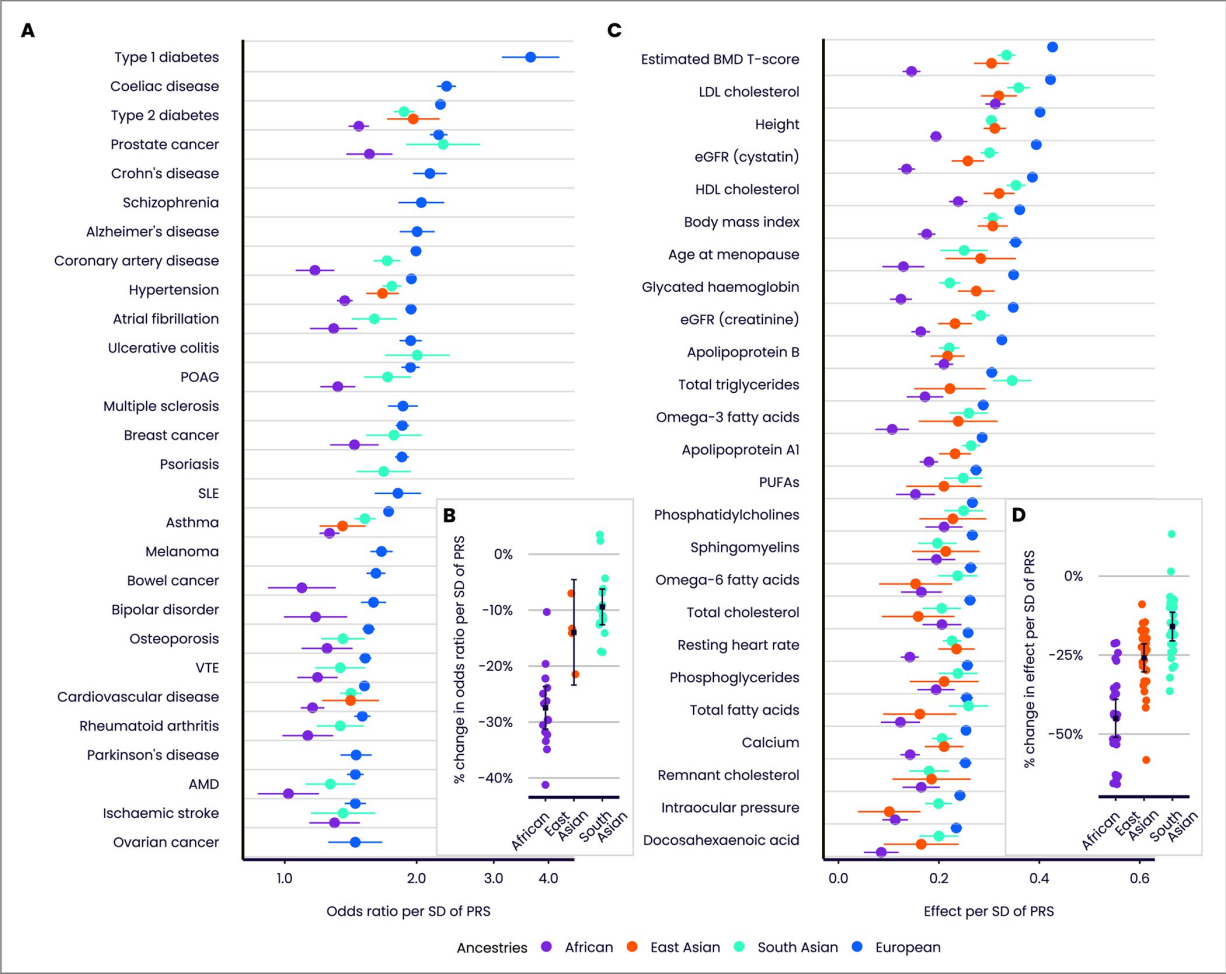
Predictive performance of the UK Biobank PRS Release (Enhanced PRS Set) by ancestry group. Performance (odds ratio, or effect on standardised quantitative trait, per SD of PRS, adjusting for age and sex), measured in the independent UKB Testing Subgroup, of the disease traits (A) and quantitative traits (C), stratified by genetically inferred ancestry. Odds ratios are shown on a log scale. Results for non-European ancestries are shown if at least 100 cases are available for testing. Relative change in performance in non-European compared to European ancestries for disease traits (B) and quantitative traits (D). Bars indicate 95% confidence intervals (CI). Refer to Fig 1 legend for disease and quantitative trait abbreviations.

Fig 3 and S3 and S4 Tables quantify performance properties of the Enhanced PRS Set, for disease and quantitative traits, in the Testing Subgroup. Performance is assessed across multiple ancestries, subject to a minimum threshold on case numbers, as reliable performance metrics cannot be evaluated in some ancestries for diseases which are rare in UKB. In individuals with European ancestries, performance in disease traits (measured by odds ratio per SD of PRS, from logistic regression adjusting for age and sex) was variable, ranging from 3.64 (type 1 diabetes) to 1.45 (epithelial ovarian cancer), with median 1.85. Performance in quantitative traits in individuals with European ancestries (measured by effect on standardised trait per SD of PRS, from linear regression adjusting for age and sex) ranged from 0.421 (estimated BMD T-score) to 0.223 (docosahexaenoic acid), with median 0.286. Similar patterns were seen for the Standard PRS Set (S1 Fig and S3, S4 Tables), and also when performance was evaluated on the disease traits using the area under the receiver operating characteristic curve (AUC) as an alternative performance metric (S2 Fig and S3 Table). In individuals with European ancestries, the AUC for the Enhanced PRS alone ranged from 0.596 (age-related macular degeneration) to 0.888 (type 1 diabetes) among disease traits, with median 0.666.

As previously reported for other PRSs [21, 22], performance in individuals with non-European genetic ancestries was generally lower than in those with European ancestries (Fig 3). Averaging across all diseases, the odds ratio per SD of PRS reduced by 9.4% in individuals with South Asian ancestries (95% CI 6.2–12.6%), and by 14.0% in individuals with East Asian ancestries (95% CI 4.5–23.4%). Consistent with previous observations, the largest reduction was in individuals with African ancestries (27.5%, 95% CI 23.6–31.3%). Reductions in effect per SD were numerically larger for the 25 quantitative traits (Fig 3D), but the OR per SD and effect per SD are on different scales [31], and so the magnitude of changes for the quantitative traits cannot be directly compared to those for the disease traits. Averaging across all quantitative traits, the effect size of the PRS reduced by 16.0% in individuals with South Asian ancestries (95% CI 11.4–20.5%), 25.9% in individuals with East Asian ancestries (95% CI 21.4–30.3%), and 45.0% in individuals with African ancestries (95% CI 39.0–51.0%). While some point estimates of performance were greater in individuals with South Asian ancestries, none of these differences were significant following correction for multiple testing.

As expected, the Enhanced PRS Set generally outperformed the Standard PRS Set (S3 Fig and S5 Table). Across the 37 diseases and quantitative traits with independent Standard and Enhanced PRS scores, there were 27 instances where the Enhanced PRS significantly outperformed (at a nominal 5% level) the Standard PRS in individuals with European ancestries, and no instance where the Standard PRS significantly outperformed the Enhanced PRS (median relative increase in Enhanced odds ratio = 1.03, range 0.97–1.24). In a separate comparison, looking across diseases and quantitative traits, the relationship between training sample size [20] and predictive performance was positive but noisy (S4 and S5 Figs), indicating that other trait-specific factors, such as heritability, genetic architecture, and prevalence, may also be important in determining performance [14, 32].

**Fig 4:**
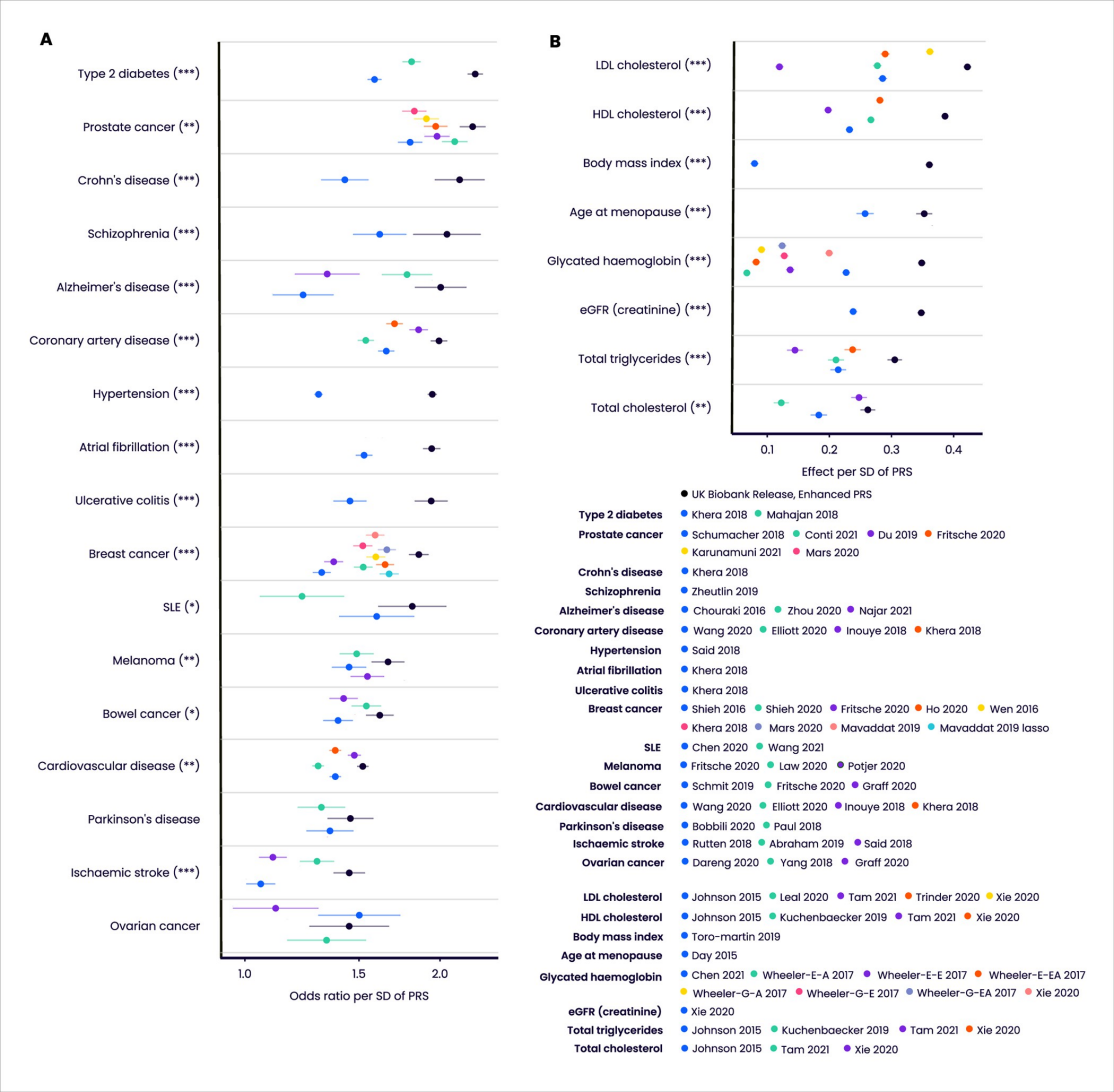
Predictive performance of the UK Biobank PRS Release against published comparator PRSs. Performance (odds ratio, or effect on standardised quantitative trait, per SD of PRS, adjusting for age and sex) in the independent UKB Testing Subgroup (European ancestries) of the Enhanced PRS sets for disease traits (A) and quantitative traits (B), for those traits for which there are published PRS algorithms (citations provided in S6 Table). Odds ratios are shown on a log scale. Bars indicate 95% confidence intervals. Asterisks indicate significance level for difference in performance between the Enhanced PRS and the nearest comparator PRS (5000 bootstraps): * p<0.05, ** p<0.01, *** p<0.001. Wheeler-E-A, Wheeler-E-E and Wheeler-E-EA refer respectively to the African, European and East Asian ancestry versions of the Wheeler 2017 PRSs for glycated haemoglobin using erythrocytic variants. Wheeler-G-A, Wheeler-G-E and Wheeler-G-EA refer respectively to the African, European and East Asian ancestry versions of the Wheeler 2017 PRSs for glycated haemoglobin using glycemic variants. Refer to Fig 1 legend for disease and quantitative trait abbreviations.

We benchmarked the UK Biobank PRS Release against 76 comparator PRS scores generated from published algorithms, across a range of disease and quantitative traits (Fig 4 and S3–S6 Tables). Among individuals with European ancestries, the odds ratio or effect per SD of the Enhanced PRS Set was larger than all comparator PRS for all diseases and traits apart from epithelial ovarian cancer, and the Enhanced PRS Set significantly outperformed (at a nominal 5% level) all comparator PRSs for all diseases apart from Parkinson’s disease and epithelial ovarian cancer (median relative increase in Enhanced odds ratio = 1.12, range 0.96–1.50), and for all quantitative traits (median change in Enhanced standardised effect size = 0.11, range 0.014–0.28). Similar patterns were seen in comparisons of the Standard PRS Set against comparators (S6 Fig and S5 Table). In most cases, the Enhanced and Standard PRSs were based on larger training data sample sizes than the corresponding best performing comparator PRS, suggesting that differential sample size was one of the factors responsible for the performance difference (S7 Fig).

**Fig 5:**
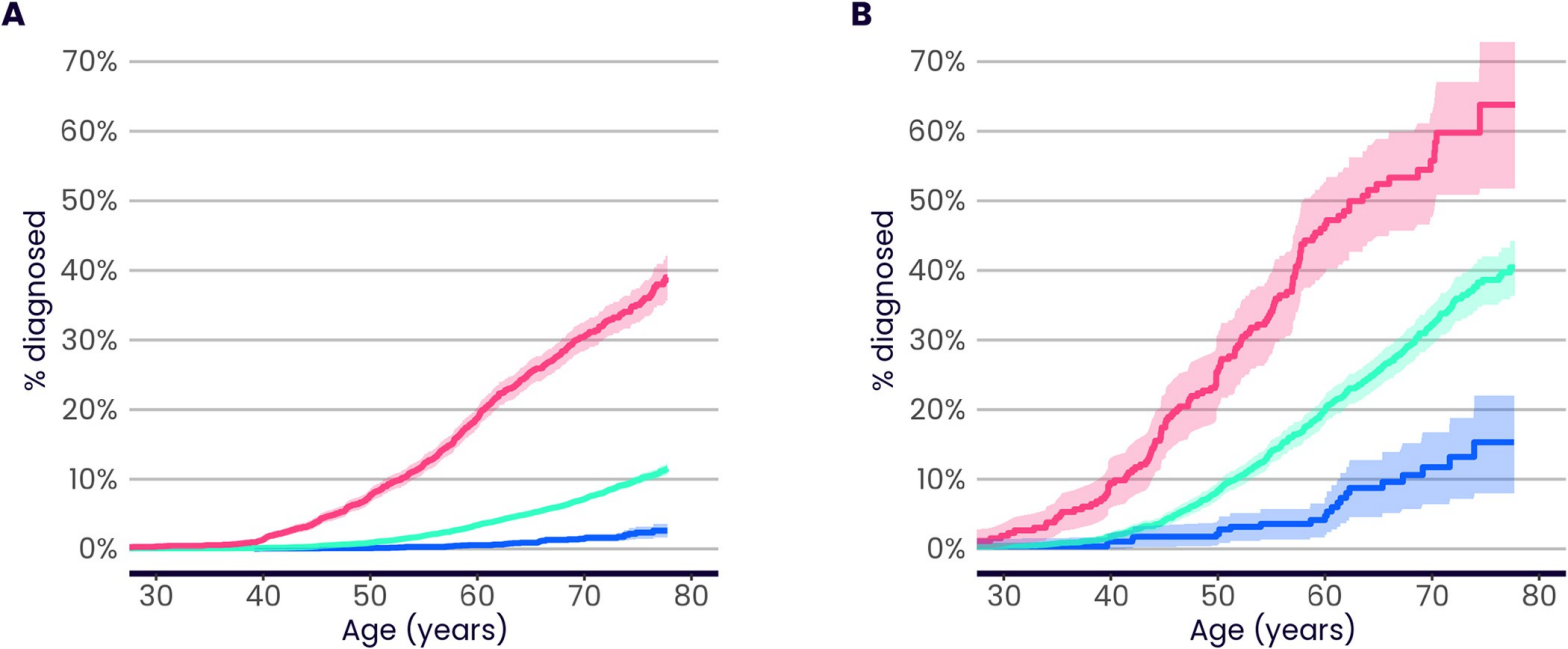
Cumulative incidence of type 2 diabetes in two ancestry groups, stratified by Enhanced PRS. Incidence is shown for the UKB Testing Subgroup with European ancestries (A) and South Asian ancestries (B). Colours indicate individuals in the highest 3% (red), median 40–60% (green) and lowest 3% (blue) of the Enhanced PRS distribution. Shaded areas indicate 95% CI.

We noted above that absolute performance of the UK Biobank PRS Release was reduced in non-European ancestries. This is also true of the comparator PRSs (S3 and S4 Tables), which indicate that, when compared within each ancestry group, the UK Biobank PRS Release performed favourably across all traits relative to comparator PRSs. We also note that differences in absolute risk can sometimes compensate for differences in discriminatory performance. For example, the odds ratio per SD of the Enhanced PRS for type 2 diabetes is lower in individuals with South Asian ancestries (OR per SD = 1.87, 95% CI 1.77–1.98), compared to European ancestries (OR per SD = 2.27, 95% CI 2.21–2.33) (S3 Table). But because the disease is more prevalent in individuals with South Asian ancestries, there is a bigger separation in absolute risk between the top and bottom 3% of the PRS distribution in individuals with South Asian compared to European ancestries (Fig 5).

### PRS risk profiles compared to high-risk variant carriers

Health systems already use genetics to identify individuals at increased risk of particular diseases, including some common diseases (e.g. breast cancer and heart disease), but to date this has focussed on carriers of high-risk rare mutations [33, 34]. PRS scores provide a way to measure a separate component of genetic risk, via the accumulation of many small-effect common variants, and so it is of interest to compare the risk profiles of these two components. Taking familial hypercholesterolemia (FH) and breast cancer (BC) as examples, and following previous work [11, 12], we find that individuals possessing high PRS scores have a cumulative incidence risk profile similar to carriers of high-risk variants in known functional genes identified from available whole exome sequencing in the same cohort.

**Fig 6:**
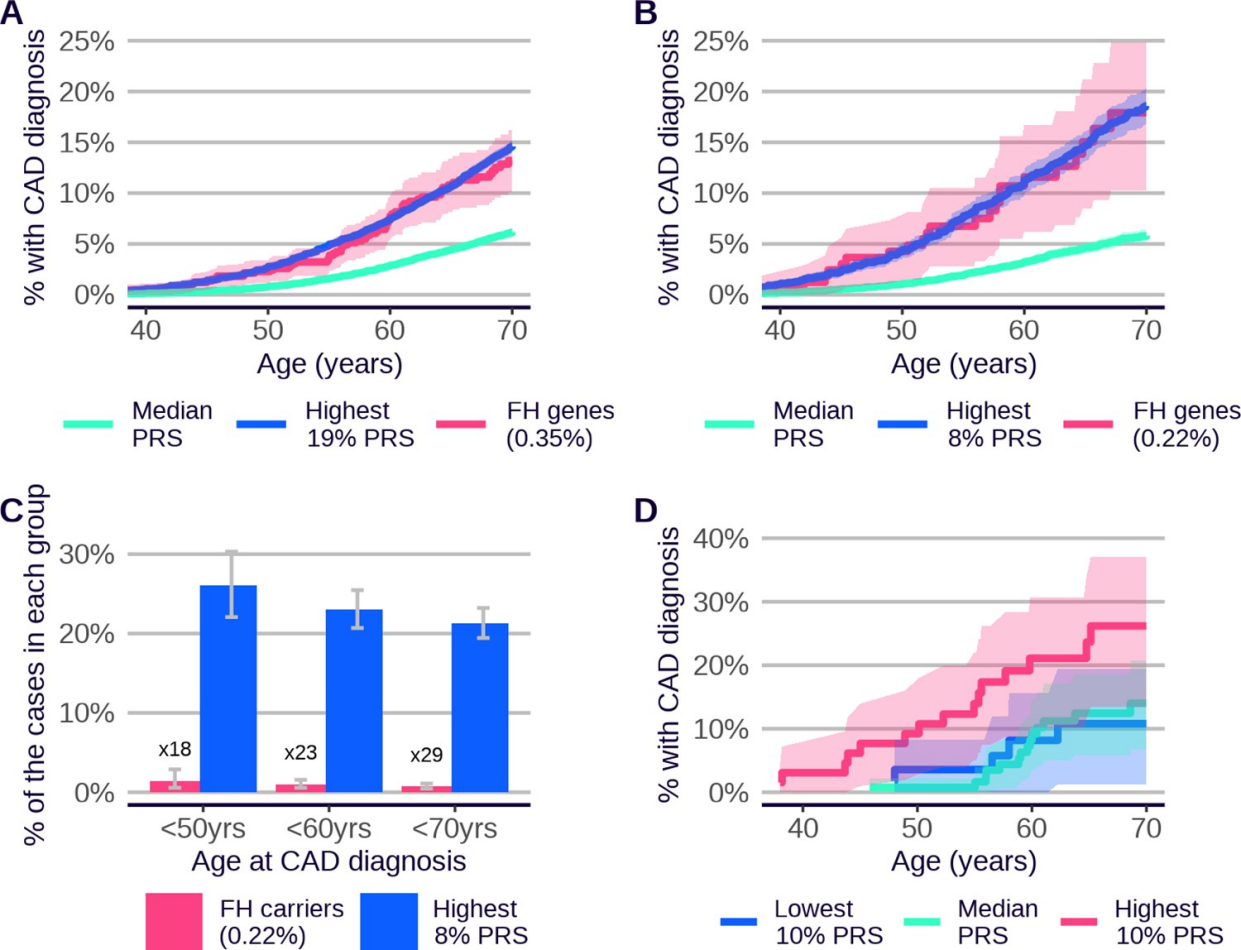
PRS risk profiles compared to functional variant carriers. A) Cumulative incidence of coronary artery disease (CAD) in familial hypercholesterolemia (FH) carriers (red, 0.35% of evaluation group), compared to individuals in the top 19% of the Enhanced CAD PRS distribution (blue, percentile chosen such that the risk up to age 70 is similar to that for mutation carriers), and the median 40–60% of the PRS (green). Carrier risks are evaluated in UKB individuals with European ancestries for whom whole exome sequencing data were available. PRS risks are evaluated in the UKB Testing Subgroup (European ancestries). B) Cumulative incidence of CAD in FH carriers (red, 0.22% of evaluation group), compared to individuals in the top 8% of the Enhanced CAD PRS distribution (blue) and the median 40–60% of the PRS (green). Carrier and PRS risks are evaluated in their respective Panel A groups, additionally restricted to those with primary care data linkage and no recorded statin prescription prior to CAD event. C) Percentage of CAD cases diagnosed in individuals aged <50, <60, or <70 years that occurred in FH carriers (red) or in individuals in the top 8% of the Enhanced PRS distribution (blue). Carrier and PRS risks are evaluated in their respective Panel B groups. The ratio between the number of high PRS cases and mutation carrier cases in each age group is shown on the plot. D) Cumulative incidence of CAD in FH carriers (evaluated as in Panel A), with additional stratification by the top 10% (red), median 40–60% (green), and bottom 10% (blue) of the Standard CAD PRS. The Standard PRS is used here to maximise the number of individuals with both whole exome sequencing data and a PRS value available for analysis. Sample size details are provided in S7 Table. Bars and shadings indicate 95% CI.

FH carriers (with a pathogenic or likely-pathogenic mutation in one of the four major FH genes *APOB*, *APOE*, *LDLR* or *PCSK9*, see S1 File) comprise 0.35% of UKB individuals with European ancestries for whom whole exome sequencing data were available (S7 Table). We find that the average risk, by age 70, of coronary artery disease (CAD) in FH carriers is 13.2% (95% CI 10.1–16.2%), in line with previous studies [35, 36]. A similar average risk is seen in individuals who are in the top 19% of the CAD Enhanced PRS distribution (risk by age 70 = 14.6%, 95% CI 14.0–15.2%, Fig 6A). Risks are higher both for mutation carriers and for high PRS individuals who are not using statins for primary prevention [37]. Restricting the analyses to individuals for whom primary care prescribing data are available and who have no recorded statin prescription (other than prescriptions following a CAD diagnosis), the average risk to age 70 in FH carriers is 17.9% (95% CI 10.3–24.9%), which is similar to that seen in statin-free individuals in the top 8% of the Enhanced PRS distribution (risk by age 70 = 18.6%, 95% CI 16.8–20.3%, Fig 6B). The proportion of high PRS individuals who are also carriers is small, and in line with the expected independence (due to low linkage disequilibrium [38]) of common-variant PRS scores with rare variation (S7 Table). Note that all analyses here have caveats. For example, while FH carriers not on statins are expected to have a higher CAD risk, these individuals might have avoided statin prescription due to lower measured cholesterol levels, an effect which will tend to reduce their CAD risk compared to other FH carriers.

For the example of female breast cancer, individuals in the top 0.3% of the breast cancer Enhanced PRS distribution have an equivalent average lifetime risk to deleterious mutation carriers for *BRCA1* or *BRCA2* genes in UK Biobank (35% to age 70, S8A Fig). We note that the level of carrier risk in UKB is lower than that reported by studies of *BRCA1/2* penetrance in women who were selected for genetic testing on the basis of a family history of breast and/or ovarian cancer (e.g. [39]), which likely include a degree of residual ascertainment bias, but is broadly in line with estimates from other unselected population-based studies (e.g. [40–42]). Considering breast cancer-associated mutations across a broader range of genes (*BRCA1*, *BRCA2*, *PALB2*, and the more moderate-risk genes, *ATM* and *CHEK2*), the average carrier risk is equivalent to the top 3% of the PRS distribution (S8B Fig, see S1 File for definition of mutation carriers).

Previous studies [11, 12] have also shown that individuals at equivalently high average risk due to PRS typically outnumber high-risk variant carriers, often massively so. Focusing on non-statin users, and on the individuals in the top 8% of the Enhanced UKB CAD PRS distribution with similar average lifetime CAD risk to FH carriers (combined carrier frequency 0.22%), the high PRS group accounts for between 18 and 29 times the number of CAD events, depending on age (Fig 6C). Both high PRS and FH mutation carriers convey a higher average risk at younger ages (S9 Fig), but the effect is stronger for FH carriers, explaining the reduced ratio when restricted to age less than 50 (Fig 6C).

Following previous work [23–25], we also find that carriers of high-risk variants can have their disease risk further modulated by their PRS score (Fig 6D), providing one explanation for the incomplete penetrance and variable expressivity seen in carriers [43]. The more-powerful PRSs described here should have a larger effect in modulating the impact of rare variants than has previously been observed.

Fig 6A and 6B display average incidence of disease with age for individuals in their respective PRS groups. Thus, our statements regarding equivalence of risk focus on average risk. We note that some of the individuals in a given PRS group will tend to have higher incidence with age, and some will have lower. This is also true for the mutation carriers—some will have higher disease risk and some will have lower risk, for a number of reasons, one of which is that the risk caused by the monogenic mutation is modulated by the individual’s PRS (Fig 6D).

### PRS risk profiles with age and sex

**Fig 7:**
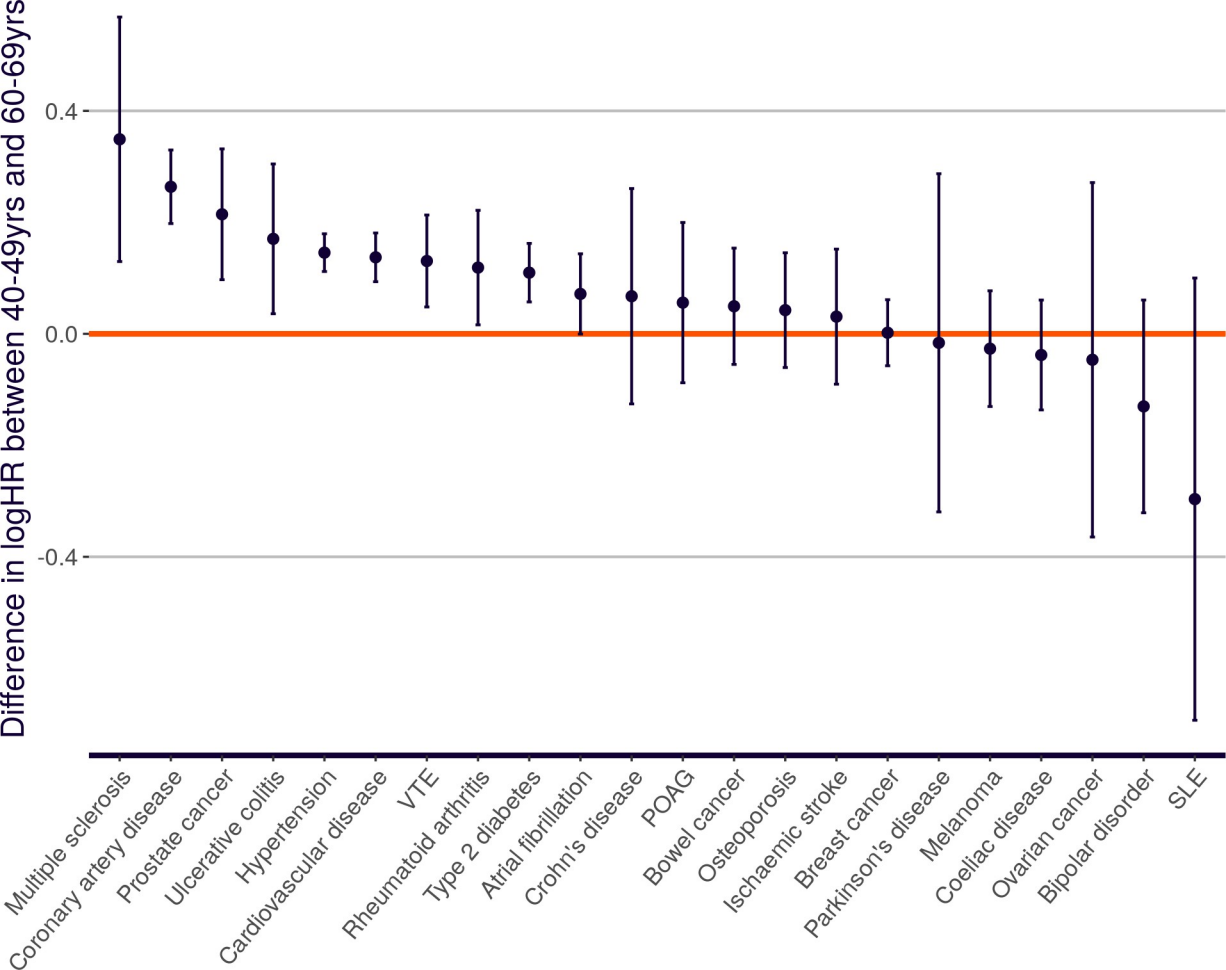
Change in Standard PRS disease effect size with age. Difference in PRS effect size (log hazard ratio per SD of PRS, based on incident events over the next 10 years) between younger (40–49) and older (60–69) age-at-first-assessment groups. Standard PRSs are presented and evaluated in all UKB individuals with European ancestries, to maximise case numbers. Alzheimer’s disease, asthma, psoriasis, schizophrenia and type 1 diabetes are omitted, because they are primarily diagnosed outside the UKB age range. Bars indicate 95% CI. Refer to Fig 1 legend for disease abbreviations.

Cumulative incidence plots across multiple traits and ancestries (Fig 2 and S10, S11 Figs) indicate that people with high PRS are at elevated risk of disease throughout the lifecourse. This observation could be clinically relevant, given that a person’s PRS score is invariant with age and can be measured early in life, and that identifying high-risk individuals at younger ages is often challenging via other methods. In addition, there is evidence for age-dependent PRS performance in some diseases, with a tendency for greater discrimination at younger ages [44], and this could add further weight to the use of PRS for risk identification in younger people. We assessed this question systematically across the diseases in the PRS Release. Fig 7 shows that many of the diseases display evidence for larger PRS effect size (log hazard ratio per SD of PRS) in younger compared to older individuals in UKB (nine out of 22 diseases with nominal significance and 13 consistent with the null of no age effect). This is in line with earlier observations of declining genetic relative risk with increasing age [44].

**Fig 8:**
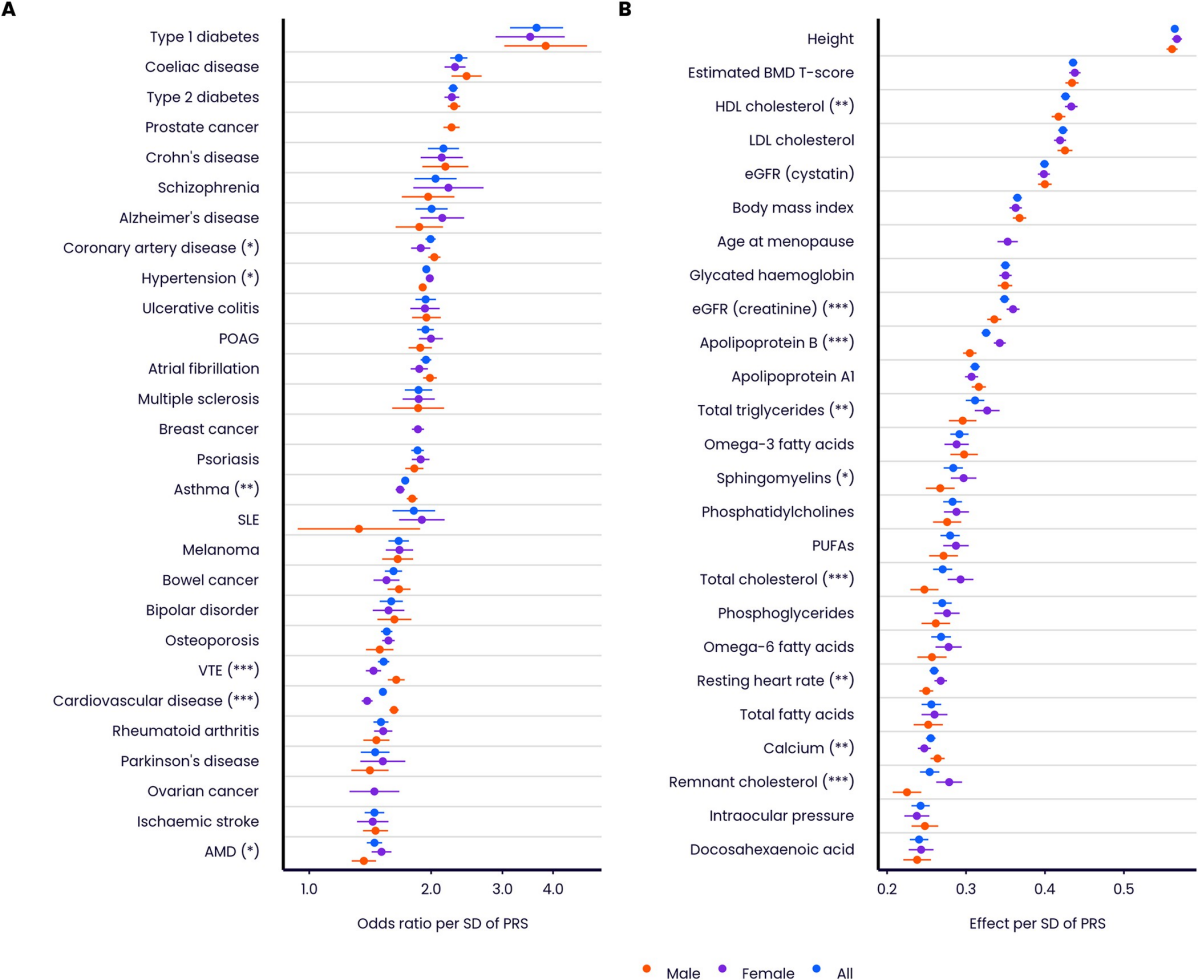
Change in PRS effect size with sex. Performance (odds ratio, or effect on sex-standardised quantitative trait, per SD of PRS, adjusting for age), measured in the independent UKB Testing Subgroup (European ancestries), of the Extended PRS set for disease traits (A) and quantitative traits (B), stratified by All (blue), Female (purple) and Male (orange). Odds ratios are shown on a log scale. Quantitative traits are standardised to zero mean and unit variance within each sex separately, and then combined for the ‘All’ analysis, generating a different effect size compared to Figs 3 and 4. Asterisks indicate two-tailed significance level for difference in performance effect size between females and males: * p<0.05, ** p<0.01, *** p<0.001. Refer to Fig 1 legend for disease and quantitative trait abbreviations.

The majority of the external training data used for the UKB PRS Release is in the form of GWAS summary statistics combined across sexes [20], and for this reason the PRSs have been trained on females and males together (apart from those for sex-specific traits). Nevertheless, we observe some sex-specific differences in Enhanced PRS performance (Fig 8, S8 Table). Significant differences (at the nominal 5% level) are seen for six disease traits (larger female effects for age-related macular degeneration and hypertension; larger male effects for asthma, cardiovascular disease, coronary artery disease and venous thromboembolic disease), and nine quantitative traits (larger female effects for apolipoprotein B, creatinine-based estimated glomerular filtration rate, HDL cholesterol, remnant cholesterol, resting heart rate, sphingomyelins, total cholesterol and total triglycerides; larger male effect for calcium).

Heritability is a determinant of PRS performance [14, 32], and our results are broadly concordant with previous studies of sex-specific heritability in UKB traits [45, 46]. Bernabeu *et al*. [45] report the same sex biases as we observe, with significant heritability differences for ischaemic heart diseases, hypertension and venous thromboembolic disease, and borderline significance for asthma (FDR-corrected p = 0.06). Age-related macular degeneration was not addressed by these authors. Flynn *et al*. [46] report concordant effects, with significant heritability differences, for apolipoprotein B, creatinine-based estimated glomerular filtration rate, HDL cholesterol and total cholesterol. Remnant cholesterol and resting heart rate were not addressed by these authors. Differences in trait processing may explain certain inconsistencies, in that Flynn *et al*. did not find significant heritability differences for calcium or total triglycerides, but instead found significant heritability differences for LDL cholesterol. Flynn *et al*. analysed covariate-corrected traits (correcting for age, genetic principal components, and several other factors), and they applied empirical corrections for the effect of statin use on LDL cholesterol, whereas we removed statin users completely for that analysis.

These sex-specific patterns may reflect differences in environmental heterogeneity between sexes, or may reflect true differences in genetic architecture. The latter would imply a benefit to sex-specific training for PRS algorithms, but there are conflicting reports on the relative importance of these two factors [45–47].

### Multivariate PRS properties

We assessed PRS correlations across the 53 diseases and quantitative traits in the UKB PRS Release. Correlations between PRS scores for the disease traits are generally low (S12 Fig, S9 and S10 Tables). The only disease-disease correlations greater than 0.5 are between closely connected diseases: coronary artery disease and cardiovascular disease (r = 0.80, Enhanced Set), hypertension and ischaemic stroke (r = 0.78, Enhanced Set) and Crohn’s disease and ulcerative colitis (r = 0.55, Standard/Enhanced Set). Correlations among PRSs for quantitative traits are stronger, with strong correlations in particular among the traits related to lipid biology, and with the strongest correlation between phosphatidylcholines and phosphoglycerides (r = 0.99, Enhanced Set). The only absolute correlations greater than 0.5 between a disease trait and a quantitative trait are those between primary open angle glaucoma and intraocular pressure, a known major risk factor (r = 0.62, Enhanced Set), and between osteoporosis and estimated bone mineral density, a diagnostic factor (r = -0.90, Enhanced Set). Comparing the Enhanced PRS for coronary artery disease to Enhanced PRSs for known risk factors, the correlation with the LDL cholesterol PRS is 0.22, while the correlation with the body mass index PRS is 0.16. Despite generally low correlations, clustering of traits by PRS scores generates relationships consistent with known biology (S12 Fig). For example, type 2 diabetes clusters with body mass index (r = 0.31, Enhanced Set) and glycated haemoglobin (r = 0.32, Enhanced Set).

The relatively low between-PRS correlations suggest that multi-PRS prediction models could be useful for analyses of general mortality. Following previous work [16, 48], we carried out separate training and testing of a stepwise regression of time from first assessment to death from any cause, using the Standard Set PRS scores for both diseases and quantitative traits as predictors. We used the same UKB Testing Subgroup for testing, and for training we used the same White British Unrelated subgroup that was used for GWAS training of the Enhanced PRS Set (see S1 File for details). For most traits, we found the expected 2:1 relationship between PRS effect size on participants’ own mortality compared to that of their parents (S13 Fig). PRS scores for common diseases including coronary artery disease (hazard ratio per SD = 1.06, 95% CI 1.04–1.08) and ischaemic stroke (hazard ratio per SD = 1.07, 95% CI 1.05–1.09) were significant determinants of participants’ all-cause mortality, as was the PRS for body mass index (hazard ratio per SD = 1.05, 95% CI 1.04–1.07). The PRS for HDL cholesterol (hazard ratio per SD = 0.97, 95% CI 0.95–0.98) was a notable protective factor. The model that included all the PRS risk factors in S13 Fig was a significantly better predictor of mortality in the UKB Testing Subgroup than a model including age-at-first-assessment and sex only, both for own mortality (change in Harrell’s C = 0.0043, 95% CI 0.0025–0.0061, p = 3.8x10^-6^) and for parental mortality (change in Harrell’s C = 0.0073, 95% CI 0.0067–0.0079, p = 9.2x10^-136^, where the model was adjusted for the participant’s age-at-first-assessment and for the parent’s sex). However, the amount of variation explained by the model was low (Royston’s [49] measure of explained variation = 1.5% for the PRS-only model on participants’ own mortality), suggesting the model is useful more for biological insight than direct prediction.

### Validation in the 100,000 Genomes Project

**Fig 9:**
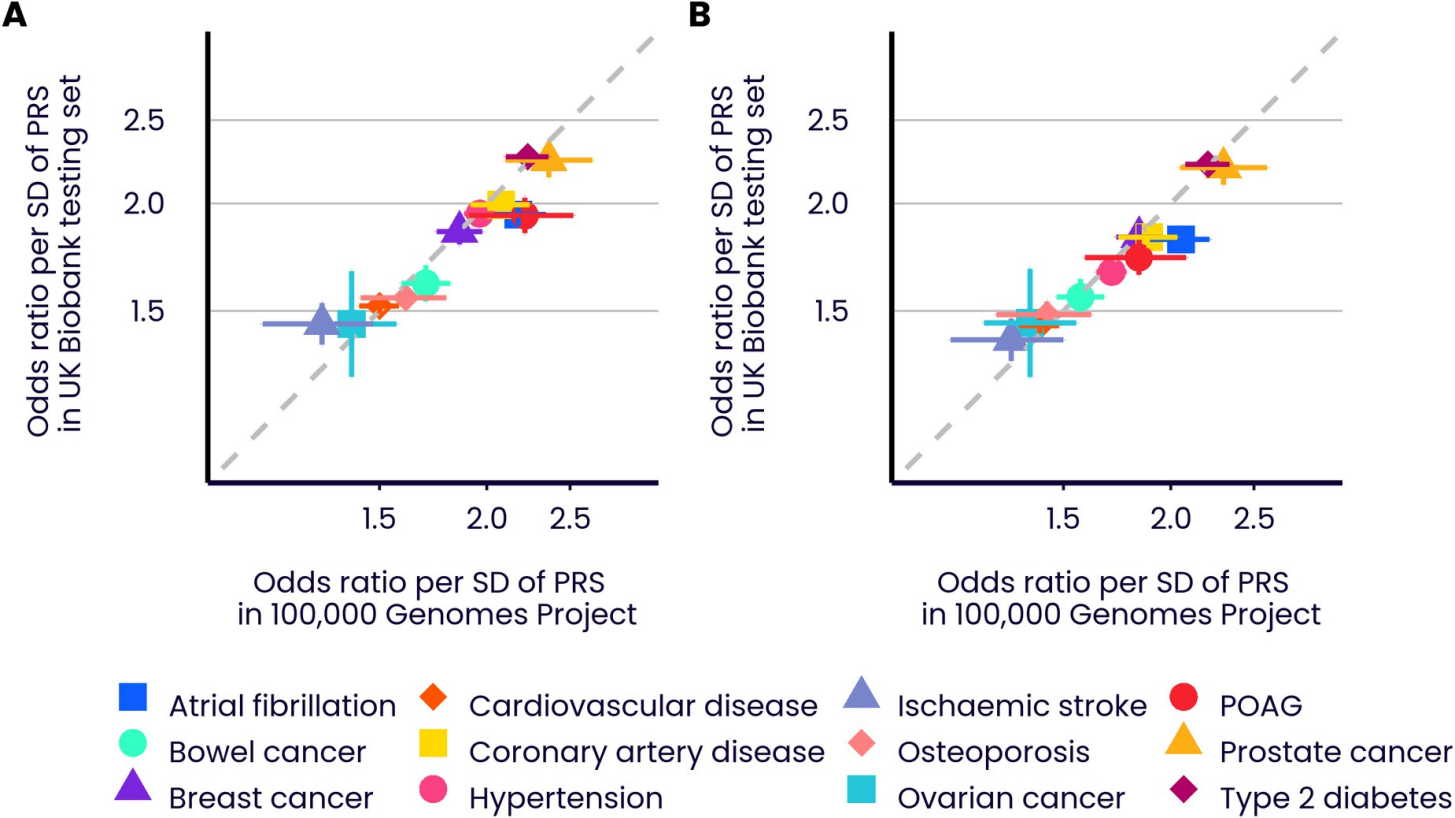
Comparative predictive performance in UK Biobank and 100,000 Genomes Project. Performance (OR per SD) across twelve diseases in the UKB Testing Subgroup and selected individuals with European ancestries from the 100,000 Genomes Project (selected to be free of rare genetic and comorbid disorders). A) Enhanced PRSs. B) Standard PRSs. Odds ratios are shown on a log scale. Coloured bars show the 95% CI of the OR per SD. Refer to Fig 1 legend for disease abbreviations.

The previous sections have validated the PRS scores as powerful predictors of disease and quantitative traits in UK Biobank. To further validate the PRS algorithms, and to guard against UKB specificity of results or overfitting, we examined their performance in the 100,000 Genomes Project [26, 27] (100KGP). This cohort is similar to UK Biobank in being UK-based (specifically, England-based), with linkage to the same UK electronic healthcare record system, but is different in being recruited either via genetic disorder probands or cancer diagnosis, and in being genetically assayed via whole-genome sequencing rather than array-based genotyping. We selected a subgroup of unrelated 100KGP individuals, excluding those with rare genetic or comorbid disorders (35,123, 3,262, 1,209, and 353 individuals with European, South Asian, African, and East Asian ancestries, respectively; see S1 File), and evaluated PRS scores for twelve diseases (which are available through application to Genomics England). Despite the differences in cohort characteristics, we found predictive performances to be similar (Fig 9; Pearson r (logOR per SD, Enhanced PRS, European ancestry) = 0.957; Pearson r (logOR per SD, Standard PRS, European ancestry) = 0.974; see S2 Table for all performance data). Reassuringly, the alignment in effect sizes holds for both the Extended and Standard PRS sets, suggesting that the use of UKB training data for the former set did not lead to cohort-specific performance improvements. The only disease with a significant difference in logOR per SD (at nominal 5% level) was atrial fibrillation, which had a higher European ancestry performance in 100KGP than in UKB for both the Enhanced PRS (OR per SD (100KGP) = 2.18 (95% CI 2.01–2.34); OR per SD (UKB) = 1.94 (95% CI 1.88–2.00)) and the Standard PRS (OR per SD (100KGP) = 2.06 (95% CI 1.89–2.22); OR per SD (UKB) of 1.82 (95% CI 1.76–1.87)).

## Discussion

The UK Biobank PRS Release has already been widely used by the research community. In this paper we have evaluated its performance in several respects, including performance comparisons with other PRS algorithms, and across ancestries, and shown the generalisability of the algorithm to another large UK-based resource, namely the 100,000 Genomes Project maintained by Genomics England.

Validating PRS performance, or comparing the performance of different PRS algorithms, is challenging, because performance is context specific. The primary requirement is for a ‘level playing field’, to correct for cohort-specific and design-specific factors such as phenotype definition and other cohort characteristics [14]. We have developed and made available a PRS evaluation tool to address this requirement. It enables robust and like-for-like comparisons of the predictive performance of different PRS scores in UKB. It should facilitate the ongoing development of PRSs by the research community.

Our comparisons show that both the Enhanced and Standard PRS scores released in the UK Biobank PRS Release are more powerful, in individuals with European and non-European ancestries, than almost all of those in a large comparator set of 76 previously released PRSs across these traits. The potential reasons for this increase in performance are varied, but difference in training sample sizes is one likely factor, especially for the Enhanced PRSs which leverage additional data from UK Biobank. The observation of similar performance in a separate UK cohort (the 100,000 Genomes Project) further validates the algorithms underlying the PRSs released in UKB. We note that making PRS scores available for a range of widely-used cohorts will become especially valuable as PRS algorithms themselves move beyond simple linear combinations of variant weights, and towards other algorithmic forms with pre- and post-processing steps [3].

The availability of powerful PRSs for 53 traits on the same large set of extensively characterised individuals has allowed a systematic study of PRS properties. PRS performance differs substantially across the diseases studied, presumably due, amongst other things, to differences in GWAS sample size [50] and genetic architecture across diseases (for example, ischemic stroke is a collection of multiple subtypes which have differing genetic risk factors [51], meaning that an algorithm that generates a single PRS for this compound phenotype will have reduced power). With a few unsurprising exceptions, within-individual pairwise correlations of PRS scores are low. The correlation between the Enhanced coronary artery disease (CAD) PRS and the Enhanced PRSs for known quantitative risk factors for CAD (e.g. LDL cholesterol: r = 0.22; body mass index: r = 0.16) are also not appreciable. Many PRSs show evidence for larger effect sizes for younger, compared to older, individuals in UKB. We also find sex-specific differences in the predictive power of some PRSs. Sex-specific differences in heritability would generate such differences, and this is broadly consistent with previous studies [45, 46]. More work is needed to explore the causes of these observed patterns.

One critical aspect is PRS performance in different ancestry groups [21, 22]. We developed our PRS evaluation tool with this in mind, maximising the representation of non-European ancestries in the Testing Subgroup of UKB and reporting ancestry-specific results for all analyses with sufficient case data. We confirmed and quantified the widely-observed diminution of performance across ancestries, with average decrease in disease OR per SD of 9.4%, 14.0%, and 27.5%, respectively for individuals with South Asian, East Asian, and African ancestries respectively, relative to the performance for individuals with European ancestries. Clearly there is an urgent need for additional GWAS data in individuals with non-European ancestries, through further studies, and where possible, release of summary statistics from existing studies, to improve PRS training data, and for improved PRS methodologies to further reduce performance differences across ancestries. We note that PRSs nonetheless have predictive power across all ancestries, and that the predictive power of the UKB Enhanced PRS in individuals with African ancestries for some diseases, such as type 2 diabetes or prostate cancer, with OR per SD of 1.48 (95% CI 1.40–1.56) and 1.56 (95% CI 1.38–1.76) respectively, are larger than those for individuals with European ancestries for other diseases, such as cardiovascular disease or age-related macular degeneration, with OR per SD of 1.52 (95% CI 1.49–1.55) and 1.45 (95% CI 1.39–1.51) respectively. Further, differences in baseline risk in different ancestries can mean that, notwithstanding diminished PRS performance, high-PRS individuals in a particular non-European ancestry group can be at higher levels of absolute risk than similarly high-PRS individuals with European ancestries (recall Fig 5, which shows much higher risk for type 2 diabetes for similar levels of PRS in individuals with South Asian compared to European ancestries in UKB). Discussion of the application of PRS in different groups should incorporate differences not only in disease specific performance, but also in baseline disease rates.

Many health systems currently have active programmes to identify carriers of rare, high-penetrance, mutations which increase risk for common diseases, such as familial hypercholesterolemia (FH) for CAD, mutations in *BRCA1*, *BRCA2*, *ATM*, *CHEK2*, and *PALB2* for breast cancer, and Lynch syndrome for bowel cancer. As others have noted [11, 12], it is now possible to identify a different set of individuals, with equivalent levels of average risk to that of rare mutation carriers, where the risk is also genetic, but driven by the cumulative impact of large numbers of common variants. Continuing improvement in PRS methodology and training data will further increase the proportion of individuals in the population at levels of risk which would attract attention in health systems if due to rare variants. For example, with the UKB Enhanced CAD PRS we have shown that the top 8% of individuals have a similar level of average CAD risk to FH mutation carriers (comparing individuals in UKB not on statins).

It seems untenable in the long term to offer interventions or enhanced screening to one group of individuals at high risk because of genetics but not to another, just because the variants contributing to risk are different. This supports the case for an equivalence-of-risk principle, in which risk-based screening guidelines developed for the management of high-risk variant carriers [33, 34] can be extended to cover individuals with equivalent risk based on their PRS. Further, the high-PRS individuals account for many fold more disease events [11, 12], an effect which will also increase as the power of PRSs continues to improve. In the FH example, the high PRS group is responsible for up to 30-fold more disease events compared to FH carriers. Increasing detection of FH carriers is, appropriately, a focus of many health systems (e.g. a key metric in the current 10-year plan for the UK NHS is to increase detection from current levels of 7% to at least 25%) [52]. These results suggest that a parallel approach to detecting high-risk individuals via PRS could have an even greater impact on disease prevention.

There are limitations to this study. Only the performance of PRS scores generated for UK Biobank and 100,000 Genomes Project are assessed, and expansion to other cohorts is limited by the proprietary nature of the PRS algorithms used to generate these scores. While the similarity in performance between these two cohorts is reassuring, further generalisability of performance is not guaranteed and may be limited by shared characteristics of the two cohorts, such as their shared UK origin.

The UK Biobank PRS Release provides well-validated PRS scores across multiple traits, and provides opportunities for subsequent research. We expect that they will evolve in time, and will be improved upon. We have provided a comparative evaluation tool in the expectation that better PRS scores will be developed, as data and methodologies improve. We anticipate the UK Biobank PRS Release will provide an ongoing platform of powerful polygenic risk scores, to enable continuing research and clinical model development.

## Supporting information

S1 File Supplementary methods and acknowledgements. (DOCX)

S1 Table Phenotype definitions for disease and quantitative traits included in the UKB PRS Release. (XLSX)

S2 Table Details of PRS evaluation cohorts. (XLSX)

S3 Table Performance of Standard, Enhanced and external comparator disease PRSs, across different ancestry groups and evaluation cohorts. (XLSX)

S4 Table Performance of Standard, Enhanced and external comparator quantitative trait PRSs, across different ancestry groups and evaluation cohorts. (XLSX)

S5 Table Comparison of performances of Standard, Enhanced and closest external comparator PRSs, evaluated in the UKB Testing Subgroup. (XLSX)

S6 Table Details of external comparator PRS algorithms. (XLSX)

S7 Table Numbers of UKB participants included in analyses comparing high PRS individuals with carriers of rare, disease-associated mutations. (XLSX)

S8 Table Performance of Enhanced PRS Set in UKB Testing Subgroup with European ancestries, stratified by participant sex. (XLSX)

S9 Table Pearson correlations between PRSs in the Standard PRS Set, estimated in the UKB Testing Subgroup with European ancestries. (XLSX)

S10 Table Pearson correlations between PRSs in the Enhanced PRS Set, estimated using the UKB Testing Subgroup with European ancestries. (XLSX)

S11 Table Mean and standard deviation of PRSs in the Testing Subgroup for all disease and quantitative traits in both the Standard and Enhanced PRS Sets, stratified by genetically inferred ancestries. (XLSX)

S1 Fig Predictive performance of the UK Biobank PRS Release (Standard Set) by ancestries. Performance (odds ratio, or effect on standardised quantitative trait, per SD of PRS, adjusting for age and sex), measured in the independent UKB Testing Subgroup, of the disease traits (**A**) and quantitative traits (**C**), stratified by genetically inferred ancestry. Results for non-European ancestries are shown if at least 100 cases are available for testing. Relative change in performance in non-European compared to European ancestries for disease traits (**B**) and quantitative traits (**D**). Odds ratios are shown on a log scale. Bars indicate 95% confidence intervals (CI). Refer to Fig 1 legend for disease and quantitative trait abbreviations.
(TIF)

S2 Fig Predictive performance (AUC) of the UK Biobank PRS Release disease traits by ancestries. Performance (area under the receiver operating characteristic (ROC) curve, or AUC), measured in the independent UKB Testing Subgroup, of the disease traits in the Standard (**A**) and Enhanced (**B**) PRS sets, stratified by genetically inferred ancestry. Results for non-European ancestries are shown if at least 100 cases are available for testing. Bars indicate 95% confidence intervals (CI). Refer to Fig 1 legend for disease abbreviations.
(TIF)

S3 Fig Comparison of the predictive performance of the Standard and Enhanced PRS sets. Performance (odds ratio, or effect on standardised quantitative trait, per SD of PRS, adjusting for age and sex), measured in the independent UKB Testing Subgroup, of the disease traits (**A, C, E, G**) and quantitative traits (**B, D, F, H**) in the Standard and Enhanced PRS sets in different ancestries. EUR = European ancestry group (**A, B**). EAS = East Asian ancestry group (**C, D**). SAS = South Asian ancestry group (**E, F**). AFR = Sub-Saharan African ancestry group (**G, H**). Bars indicate 95% confidence intervals (CI). Traits with highest and lowest Enhanced PRS performance are labelled. For trait codes see S1 Table.
(PNG)

S4 Fig Relationship between disease trait predictive performance and GWAS effective sample size across genetically inferred ancestry groups. **A** Relationship between the ancestry-specific odds ratio and effective sample size (across all training GWASs) for the Enhanced PRS Set [20]. **B** Relationship between relative change in odds ratio and relative change in effective sample size, comparing the Enhanced to the Standard PRS Set [20]. Effective sample size is defined as 4∑*_j_n_j_c_j_*(1−*c_j_*), where *n_j_* and *c_j_* are respectively the total sample size and the proportion of cases for the *j*th constituent GWAS for a given trait. Only those diseases with non-overlapping samples in the constituent GWASs are displayed. Odds ratios are shown on a log scale. Bars indicate 95% confidence intervals. Dashed lines indicate linear regression slopes, with p-values and asterisks indicating the significance of the slope (* p<0.05, ** p<0.01, *** p<0.001). Refer to Fig 1 legend for disease abbreviations.
(PNG)

S5 Fig Relationship between quantitative trait predictive performance and GWAS sample size across genetically inferred ancestry groups. Relationship between the ancestry-specific effect on standardised quantitative trait, per SD of PRS, and sample size (across all GWASs) for the Enhanced PRS Set [20]. Only those traits with non-overlapping samples in the constituent GWASs are displayed. Bars indicate 95% confidence intervals. Dashed lines indicate linear regression slopes, with p-values and asterisks indicating the significance of the slope (* p<0.05, ** p<0.01, *** p<0.001). Refer to Fig 1 legend for quantitative trait abbreviations.
(PNG)

S6 Fig Predictive performance of the UK Biobank PRS Standard Release against published comparator PRSs. Performance (odds ratio, or effect on standardised quantitative trait, per SD of PRS, adjusting for age and sex) in the independent UKB Testing Subgroup (European ancestries) of the Standard PRS sets for disease traits (**A**) and quantitative traits (**B**), for those traits for which there are published PRS algorithms (citations provided in S6 Table). Odds ratios are shown on a log scale. Bars indicate 95% confidence intervals. Asterisks indicate significance level for difference in performance between the Standard PRS and the nearest comparator PRS (5000 bootstraps): * p<0.05, ** p<0.01, *** p<0.001. Wheeler-E-A, Wheeler-E-E and Wheeler-E-EA refer respectively to the African, European and East Asian ancestry versions of the Wheeler 2017 PRSs for glycated haemoglobin using erythrocytic variants. Wheeler-G-A, Wheeler-G-E and Wheeler-G-EA refer respectively to the African, European and East Asian ancestry versions of the Wheeler 2017 PRSs for glycated haemoglobin using glycemic variants. Refer to Fig 1 legend for disease and quantitative trait abbreviations.
(TIF)

S7 Fig Comparison of training data effective sample sizes **(A)** and total samples (B) for disease and quantitative traits, among Comparator, Standard and Enhanced PRS. Total training sample sizes for the Enhanced PRS, Standard PRS, and the best-performing comparator PRS, for disease traits (**A**) and quantitative traits (**B**) [20]. For disease traits, the x-axis is the effective sample size, defined as 4 / ((1/n_0_) + (1/n_1_)), where n_0_ is the total number of controls, and n_1_ is the total number of cases. Where training data came from meta-analysis of multiple GWASs, the total numbers are used, as it was not always possible to obtain accurate per-GWAS numbers for the PGS Catalog PRSs. A dashed connecting line is used where the comparator PRS sample size is larger than the Enhanced PRS sample size. Only those traits for which at least one comparator PRS was available are displayed. Traits with overlapping samples in the Standard/Enhanced PRS training are excluded. In addition, ischaemic stroke is not shown, because the best comparator PRS (Abraham et al 2019, doi: 10.1038/s41467-019-13848-1) was trained using a complex combination of PRSs for 19 different diseases and quantitative traits; hypertension is also excluded because the only comparator PRS (Said et al 2018, doi: 10.1001/jamacardio.2018.1717) used a combination of disease and quantitative trait data. Refer to Fig 1 legend for disease and quantitative trait abbreviations.
(PDF)

S8 Fig Comparative cumulative incidence plots in high-risk breast cancer gene mutation carriers and high-PRS individuals of equivalent risk. Cumulative incidence of breast cancer for carriers of high-risk mutations in breast cancer associated genes (red), compared to individuals in the top fraction of the PRS distribution (blue) corresponding to equivalent risk, and the median 40–60% of the PRS (green). Carrier risks are evaluated in UKB women with European ancestries for whom exome sequencing data are available. PRS risks are evaluated in the UKB Testing Subgroup (European ancestries, female). **A**, Incidence of breast cancer in *BRCA1*+*BRCA2* loss-of-function variant carriers (0.4% of evaluation group) vs top 0.3% of breast cancer Enhanced PRS. **B**, Incidence of breast cancer in combined *BRCA1*+*BRCA2*+*ATM*+*CHEK2*+*PALB2* loss-of-function variant carriers (1.34% of evaluation group) vs top 3% of breast cancer Enhanced PRS. Sample size details are provided in S7 Table. Shaded areas indicate 95% CI.
(TIFF)

S9 Fig Effects of familial hypercholesterolemia (FH) gene mutations and PRS on risk of coronary artery disease (CAD), by age of diagnosis. The relative influence on CAD risk (hazard ratio) of FH carrier vs non-carrier status (pink), and of high vs median (40–60%) Enhanced CAD PRS status (blue). **A** Analyses for the top 19% of the Enhanced CAD PRS. Carrier risks are evaluated in UKB individuals with European ancestries for whom whole exome sequencing data were available; PRS risks are evaluated in the UKB Testing Subgroup (European ancestries). **B** Analyses for the top 8% of the Enhanced CAD PRS. Carrier and PRS risks are evaluated in their respective Panel **A** groups, additionally restricted to those with primary care data linkage and no recorded statin prescription prior to CAD event. Sample size details are provided in S7 Table. Bars represent 95% CI.
(TIFF)

S10 Fig Cumulative incidence plots by ancestries for the Standard PRS Set. Cumulative incidence plots are shown for each disease and each ancestry group in the UKB Testing Subgroup, provided more than 40 cases are available (the number of cases is printed otherwise), with separate curves for the highest 3% (red), lowest 3% (blue), and median 40–60% (green) of the PRS distribution. **A**. Alzheimer’s disease (AD). **B**. Atrial fibrillation (AF). **C**. Age-related macular degeneration (AMD). **D**. Asthma (AST). **E**. Breast cancer (BC), **F**. Bipolar disorder (BD), **G**. Coronary artery disease (CAD). **H**. Crohn’s disease (CD). **I**. Coeliac disease (CED). **J**. Bowel cancer (CRC). **K**. Cardiovascular disease (CVD), **L**. Epithelial ovarian cancer (EOC). **M**. Hypertension (HT). **N**. Ischaemic stroke (ISS). **O**. Melanoma (MEL). **P**. Multiple sclerosis (MS). **Q**. Osteoporosis (OP). **R**. Prostate cancer (PC). **S**. Parkinson’s disease (PD). **T**. Primary open angle glaucoma (POAG). **U**. Psoriasis (PSO). **V**. Rheumatoid arthritis (RA). **W**. Schizophrenia (SCZ). **X**. Systemic lupus erythematosus (SLE). **Y**. Type 1 diabetes (T1D). **Z**. Type 2 diabetes (T2D). **AA**. Ulcerative colitis (UC). **AB**. Venous thromboembolic disease (VTE). EUR = European ancestry group. EAS = East Asian ancestry group. SAS = South Asian ancestry group. AFR = Sub-Saharan African ancestry group. Shaded areas indicate 95% CI.
(PDF)

S11 Fig Cumulative incidence plots by ancestries for the Enhanced PRS Set. Cumulative incidence plots are shown for each disease and each ancestry group in the UKB Testing Subgroup, provided more than 40 cases are available (the number of cases is printed otherwise), with separate curves for the highest 3% (red), lowest 3% (blue), and median 40–60% (green) of the PRS distribution. **A**. Alzheimer’s disease (AD). **B**. Atrial fibrillation (AF). **C**. Age-related macular degeneration (AMD). **D**. Asthma (AST). **E**. Breast cancer (BC), **F**. Bipolar disorder (BD), **G**. Coronary artery disease (CAD). **H**. Crohn’s disease (CD). **I**. Coeliac disease (CED). **J**. Bowel cancer (CRC). **K**. Cardiovascular disease (CVD), **L**. Epithelial ovarian cancer (EOC). **M**. Hypertension (HT). **N**. Ischaemic stroke (ISS). **O**. Melanoma (MEL). **P**. Multiple sclerosis (MS). **Q**. Osteoporosis (OP). **R**. Prostate cancer (PC). **S**. Parkinson’s disease (PD). **T**. Primary open angle glaucoma (POAG). **U**. Psoriasis (PSO). **V**. Rheumatoid arthritis (RA). **W**. Schizophrenia (SCZ). **X**. Systemic lupus erythematosus (SLE). **Y**. Type 1 diabetes (T1D). **Z**. Type 2 diabetes (T2D). **AA**. Ulcerative colitis (UC). **AB**. Venous thromboembolic disease (VTE). EUR = European ancestry group. EAS = East Asian ancestry group. SAS = South Asian ancestry group. AFR = Sub-Saharan African ancestry group. Shaded areas indicate 95% CI.
(PDF)

S12 Fig Heatmaps of correlations among PRS scores. Correlations (calculated from individuals with European ancestries in the UKB Testing Subgroup) among diseases and quantitative traits for the Standard Set (**A**) and Enhanced Set (**B**), ordered according to a hierarchical clustering dendrogram (complete linkage on Euclidean distance, see hclust() function in R). See S1 Table for trait code mappings.
(PDF)

S13 Fig Hazard ratios from multivariate PRS modelling of all-cause mortality in UKB participants and their parents. Traits are shown if selected both by stepwise regression of participant’s time-to-death from first assessment and also by stepwise regression of their parents’ age at death (maternal and paternal data entered as separate observations). Hazard ratios shown on a log scale. See S1 Table for trait code mappings. Dashed line shows the expected parent:offspring log(hazard ratio) ratio of 1:2. A natural explanation for the larger than expected effect of CAD and ISS PRSs on parental mortality is that these diseases were bigger killers in the past, and so made up a larger proportion of all-cause mortality.
(PNG)
