# Supplementary material for: A systematic evaluation of the performance and properties of the UK Biobank Polygenic Risk Score (PRS) Release
Source: PLoS One. 2024 Sep 18;19(9):e0307270. doi: 10.1371/journal.pone.0307270 (PMC11410272; doi:10.1371/journal.pone.0307270)
Supplement: S1 File — (DOCX) [file pone.0307270.s001.docx]

# A systematic evaluation of the performance and properties of the UK Biobank Polygenic Risk Score (PRS) Release - Supplementary Information

# Data and code availability

The UK Biobank PRS Release is available via application to the UK Biobank’s Research Access Platform (Category 300, <https://biobank.ndph.ox.ac.uk/ukb/label.cgi?id=300>). PRS scores calculated in the 100,000 Genomics Project are available via application to the Genomics England Research Environment. The Evaluation Tool is available as a command line tool within the Research Access Platform. Source code for the Evaluation Tool is available at <https://github.com/Genomicsplc/ukb-pret>.

# Supplementary methods

## UK Biobank

The UK Biobank (UKB) is a UK based prospective cohort of ~500,000 individuals, recruited via postal invitation to 22 sites in the UK, aged 40-69 when recruited between 2006 and 2010 [[1,2]](https://www.zotero.org/google-docs/?90gmbb). Genotype data were generated using a custom Axiom genotyping array assaying 825,927 genetic variants, followed by genome-wide imputation. Phenotype information was obtained using a combination of self-reported information, including basic demographics, diet, and exercise habits; extensive physical and cognitive measurements; and other sources of health-related information such as medical records, death records and cancer registers which allow follow-up over the course of the participants’ lives. Additional biomarker and imaging data are also available. See Bycroft et al [[2]](https://www.zotero.org/google-docs/?FIR8No) for further details.

At the time of recruitment, all UK Biobank participants were provided with an information leaflet and were given the opportunity to ask questions about the project. They consented to the statement “I give permission for access to my medical and other health-related records, and for long-term storage and use of this and other information about me, for health-related research purposes (even after my incapacity or death)” by tapping “I agree” on a touch-screen monitor, followed by a recording of their signature using a stylus on an electronic signature pad. A recruitment staff member determined whether the participant had the mental capacity to provide informed consent, and participants were told they could withdraw their consent at any time (see https://www.ukbiobank.ac.uk/explore-your-participation/basis-of-your-participation). Our research project (project application number 9659) was approved by the UK Biobank according to their established access procedures [[3]](https://www.zotero.org/google-docs/?EbacpV), and legal and ethical approval is covered by the Research Tissue Bank approval obtained from the UK Biobank’s governing Research Ethics Committee (REC 16/NW/0274), as recommended by the National Research Ethics Service.

PRS scores for the Standard PRS Set, built using GWAS datasets external to UK Biobank, have been made available for all 486,087 UKB individuals (after removal of exclusions and withdrawals); PRS scores for the Enhanced PRS Set, built using GWAS datasets both internal and external to UK Biobank, have been made available for all 148,936 UKB individuals that were not used as part of GWAS training [[4]](https://www.zotero.org/google-docs/?r082Bf). GWAS training for the Enhanced PRS Set was performed in the White British Unrelated (WBU) subgroup, comprising 337,151 UKB individuals defined as the intersection of two sample groups created by Bycroft et al [[2]](https://www.zotero.org/google-docs/?vkqOdc): the ‘White British ancestry’ group (UKB Data Field 22006) and the ‘used in genetic principal components’ group (UKB Data Field 22020), the latter being high quality samples that were filtered to avoid closely related individuals.

To create the UKB Testing Subgroup, the group of non-WBU individuals was filtered further to ensure no relatives of 2nd degree relatedness or above with the WBU sample were included (KING [[5]](https://www.zotero.org/google-docs/?5FmLYY) kinship coefficient threshold = 2^-3.5^ = 0.0884). The remaining individuals (the Testing Subgroup) were used for evaluation of both the Standard and Enhanced PRS Sets. This subgroup includes 97,608, 9,542, 9,476 and 2,864 individuals with European, South Asian, African, and East Asian ancestries, respectively (see the “Genetic ancestry inference” section below for details of the ancestry estimation methods). There were too few individuals with Native/Indigenous American ancestries (N=389) to be included for evaluation. Values of mean(PRS) and sd(PRS) for each trait in each ancestry are provided in S11 Table.

Disease and quantitative trait variables within UK Biobank were created from a combination of Hospital Episode Statistics, Cancer Registry reports (where applicable), primary care records (for type 1 and type 2 diabetes) and self-report responses. Exclusion criteria resulted in some phenotypes being set to ‘missing’ for some individuals, for example, low density lipoprotein cholesterol levels were set to missing for individuals on statin medication. For details of phenotype definitions, see S1 Table. Various *ad hoc* QC checks were carried out for errors and inconsistencies in UK Biobank data fields; these indicated only a small fraction of remaining individuals are affected. Additional filters and case censoring were applied for PRS performance evaluations and analyses of PRS properties (for details, see ‘PRS performance evaluation’ section).

## Genetic ancestry inference

Genome-wide genetic data for UK Biobank participants were used to estimate the proportion of each genome that could be ascribed to each one of five high-level ancestry groupings: Sub-Saharan African (AFR), Native/Indigenous American (AMR), East Asian (EAS), European (EUR), and South Asian (SAS). We note that these proportions are more accurately described as “ancestry-like” genetic similarity relationships [[6]](https://www.zotero.org/google-docs/?txFjxq). To obtain these estimates, Principal Component Analysis (PCA) was first applied to a subset of common SNP genotypes in the 1000 Genomes reference dataset using standard methods [[7]](https://www.zotero.org/google-docs/?i2CIa3). To select SNPs for PCA, HapMap3 variants were intersected with a collection of well-imputed variants across UK Biobank, GSA, and OMNI arrays. SNPs were excluded if they had low call-rate (< 0.05) or had MAF<0.02 in 1000 Genomes. SNPs surviving exclusions were then LD-pruned (using plink --indep 50 5 2) to an approximately independent set of around 185,000 SNPs across the autosomes. For PCA, the genotypes were mean-centred, but not standardised.

Centroid coordinates for ancestry groups in PC space (defined from the first four PC axes) were obtained from those 1000 Genomes Project [[8]](https://www.zotero.org/google-docs/?tW8xwk) individuals belonging to population codes as follows (excluding populations that were found empirically to be highly admixed): AFR (ESN, GWD, LWK, MSL, YRI); AMR (MXL, PEL); EAS (CDX, CHB, CHS, JPT, KHV); EUR (CEU, FIN, GBR, IBS, TSI); SAS (BEB, GIH, ITU, PJL, STU). Genotype data from individuals outside the 1000 Genomes cohort were then projected onto the same PC axes to obtain their PC scores. The relationship between each individual’s vector of PC scores and each centroid was determined via a softmax transformation (base=exp(3)) applied to the cosine similarity of the two vectors, to obtain the estimated ancestry proportions. Individuals were assigned the superpopulation hard-call for which they presented the highest ancestry proportion, such that no individual was labelled as admixed.

## Comparator PRS Set

The PGS Catalog database (<https://www.pgscatalog.org/>) was searched (30th November 2021) to identify comparator PRS algorithms for any of the traits of interest (S6 Table). PRS algorithms were excluded if they had used any UKB GWAS data in the training stage (algorithms based on non-UKB GWAS data which had only used UKB data for the training of hyperparameters were not excluded). PRS algorithms were also excluded if they were designed for specific subtypes of the disease or trait, or were superseded by a more recent algorithm from the same research group, or were duplicates. Where the same research group had uploaded multiple algorithms for the same trait to the PGS Catalog, each using different statistical methodologies or different parameters, we selected the one which the authors reported as having the best performance. Where several groups had reported PRS algorithms for a given trait which were all simple lists of GWAS-significant SNPs reported in the literature, we selected the most recent algorithm. We included algorithms which appeared to be similar to other algorithms, but which had been specifically optimised for non-European ethnicities or ancestries. Additional PRS algorithms were identified by regular monitoring of relevant publications, pre-print websites, social media and by conference attendance.

For Crohn’s disease and ulcerative colitis, because a well-powered comparator PRS was not available for these diseases specifically, a comparator PRS for inflammatory bowel disease was used (which is the union of these two diseases). For cardiovascular disease, because a well-powered comparator PRS was not available for this disease specifically, comparator PRSs for coronary artery disease were used (which is a subcomponent of cardiovascular disease).

Comparator algorithms were excluded at the QC stage if there was insufficient SNP information to allow the algorithm to be implemented, or if >10% of the SNPs in the algorithm were not present in the imputed UKB dataset. Post-processing steps (PRS centering and standardisation) were applied to comparator PRS in an equivalent way to the UK Biobank PRS Release sets.

## PRS performance evaluation

All three PRS sets - the Standard Set, the Enhanced Set, and the Comparator PRS Set - were evaluated in the same multi-ancestry Testing Subgroup in UKB (see above) and in the 100,000 Genomes Project cohort (see below) (S2 Table).

Additional filtering and case censoring was performed on UKB phenotype data prior to PRS performance evaluations and analyses of PRS properties. Individuals with conflicting case status vs incident status were excluded. For melanoma and for breast, prostate and colorectal cancers, individuals were excluded from analyses if a self-reported cancer was not also present in either the Cancer Registry or Hospital Episode Statistic (HES) linkage data. Unless censored by death, a formal withdrawal from the project, or by a hard cutoff date of 1 March 2020, linkage to national HES data was assumed complete for all individuals up to 10 years from first assessment (12 years for age related macular degeneration), but then taken as censored after that time. All case events that occurred more than 10 years after first assessment (12 years for age related macular degeneration), after age 80 years, or after the hard cutoff date of 1 March 2020, were treated as controls. In addition to the above filters, cases with a missing date of diagnosis were excluded from all analyses that involved survival modelling.

Performance evaluation in a given ancestry group required a minimum of 100 cases in that ancestry group for disease traits, and 100 samples in that ancestry group for quantitative traits. The AMR ancestry group was excluded for all traits due to low sample size. For evaluation in the 100,000 Genomes Project, a minimum of 75 cases was required. For an overview of sample sizes in the evaluation cohorts, see S2 Table.

PRS performance was evaluated based on several metrics (main text and S3-S4 Tables). For all disease traits, odds ratios (from logistic regression on prevalent plus incident data) are reported per standard deviation (SD) of PRS. We also report the odds ratios for individuals in the top 3%, 5% and 10% of the PRS distribution against the rest, but we note that this top-*x*%-vs-the-rest metric does not scale monotonically with *x*, which makes it an unsatisfactory performance metric that we provide only to allow comparison with other publications where it is used. Age at recruitment and sex were added as covariates in the logistic regression where possible, noting that sex could not be added as a covariate for analyses within a single sex. For disease traits, the hazard ratios per SD (from Cox regression on prevalent plus incident data, with age and sex as covariates where possible) are reported, and also the hazard ratios for individuals in the top 3%, 5% and 10% against the median 40-60% of the PRS distribution. For quantitative traits, the regression coefficients (from linear regression, with age and sex as covariates where possible) on the standardised trait scale are also reported. For disease traits, the area under the receiver operating characteristic curve (AUC) is also reported. Standard errors for AUC were found using an implementation of deLong’s algorithm [[9]](https://www.zotero.org/google-docs/?YsQncO). For quantitative traits, the variance explained (r^2^) of the trait by the PRS (from linear regression without covariates) is also reported. Standard errors for r^2^ were found by Fisher’s z-transformation. Standard errors for relative changes in odds ratios, AUC, and r^2^, correcting for correlations induced by calculating PRSs in the same individuals, were found using a stratified (within-case/within-control) bootstrapping procedure.

## Analyses of PRS properties

The pattern of age-specific risks associated with different PRS groups was displayed using “pure risk” cumulative incidence plots (one minus the Kaplan-Meier estimate of the probability of remaining disease-free) [[10]](https://www.zotero.org/google-docs/?2PWNAk).

Whole exome sequencing data were available for 189,954 UKB participants with European ancestries. Carriers of deleterious mutations in either a breast cancer (BC) risk gene (loss-of-function mutations in *ATM*, *BRCA1*, *BRCA2* or *PALB2,* with classification of a mutation as loss-of-function based on a “high-confidence” scoring using the LOFTEE software [[11]](https://www.zotero.org/google-docs/?kcbfI3)*,* or the *CHEK2* 1100delC mutation) or a familial hypercholesterolemia (FH) risk gene (pathogenic or likely-pathogenic variants in *APOB*, *APOE*, *LDLR* or *PCSK9*, as classified by using the American College of Medical Genetics and Genomics (ACMG) guidelines [[12]](https://www.zotero.org/google-docs/?LIEMgn), or the p.Leu167del in *APOE* as previously published [[13]](https://www.zotero.org/google-docs/?YVnRV9), and extending the list provided by Fahed et al [[14]](https://www.zotero.org/google-docs/?068wBp) by moving from previous 50k UKB exome dataset to the newer 200k UKB exome dataset) were identified. BC and coronary artery disease (CAD) hazard ratios for carriers compared to non-carriers were calculated. BC and CAD hazard ratios were also estimated in the UKB Testing Subgroup (European ancestries), using the upper fraction of the Enhanced PRS distribution compared to the 40-60^th^ percentiles, and the upper fraction for which the hazard ratio empirically matched the carrier hazard ratio was found. For the BC analysis, follow-up was additionally censored at age of bilateral mastectomy (extracted from HES records). The CAD analyses were repeated in the subset of UKB participants for whom primary care prescribing data were available and who did not have a reported prescription for statin medication (other than prescriptions which began after the CAD diagnosis). Statin prescriptions were extracted from the UKB primary care data linkage dataset, using read_v2 codes bxd*, bxe*, bxg*, bxi*, bxj*, bxk* and bxl*, BNF codes 02.12.02.00.00 and 02.12.04.00.00, or via text searches for “atorvastatin”, “cerivastatin”, “ezetimibe”, “fluvastatin”, “pravastatin”, “rosuvastatin”, “simvastatin” and associated brand names (the LDL-lowering drug ezetimibe was included in the list, although in practice ezetimibe prescriptions were almost always seen in individuals also receiving a statin prescription). After excluding statin users, 164 of the 656 FH mutation carriers and 30,271 of 82,335 individuals in the UKB Testing Subgroup with European ancestries were included in the evaluation analyses. Sample numbers are provided in S7 Table.

Age-specific hazard ratios were calculated by splitting the age-at-first-assessment into 10-year age bins, and then using Cox regression (adjusted for sex, where appropriate) to estimate an incident hazard ratio within each age bin, allowing a maximum of 10 years of follow-up.

Sex-specific PRS effect sizes for quantitative traits were calculated from linear regression (adjusted for age) applied to traits that were standardised to zero mean and unit variance within each sex separately.

Forwards-backwards stepwise regression was used to find the best-fitting linear combination of PRS scores for predicting all-cause mortality. The WBU subset of UKB was used to train a Cox proportional-hazards model, with years from first assessment until participant’s death or censoring as the time variable, searching over all 39 Standard PRS scores (28 diseases plus 11 quantitative traits). Model selection was based on the Akaike information criterion, with significance threshold 0.05. A second forwards-backwards stepwise regression was then applied to parental mortality data. Data for the mother and father of each participant (excluding those who reported that they had been adopted as a child) were taken as separate observations, using their offspring’s PRS scores as predictors. For each parent, follow-up was from their birth until either their age at their offspring’s UKB assessment or their age at death, as reported by the participant at their first UKB assessment. Ideally, parental follow-up would begin at the parent’s age when the participant was born, to avoid immortal time bias [[15]](https://www.zotero.org/google-docs/?sTXiIa), but this age is not available for parents who died before UKB assessment. We therefore assumed a minimal effect of immortal time bias on parental outcomes, and this assumption is borne out by the observed 2:1 ratio between PRS effect sizes in the “own mortality” and “parents’ mortality” analyses (S13 Fig). Traits which were selected by both the “own mortality” and “parents’ mortality” stepwise regression analyses were then entered into a final training step, in which the PRS scores for these traits were entered as covariates, together with participants’ age at first recruitment and sex (of the participant, or of the mother or father, as applicable) as additional covariates, into separate “own mortality” and “parents’ mortality” Cox proportional-hazards models. Coefficients from these final models were then fixed and evaluated in the remaining UKB Testing Subgroup. Differences in Harrell’s C statistic, in models with and without PRS covariates, were tested via a z-test on the difference, using an estimated variance that accounts for covariance in C statistics and derived from an infinitesimal jackknife estimator [[16]](https://www.zotero.org/google-docs/?kEjby4), and 95% confidence intervals for the difference in C-statistics were estimated using 1,000 bootstrap samples.

## 100,000 Genomes Project

The 100,00 Genomes Project (100KGP), run by Genomics England, consists of more than 100,000 whole-genomes sequences, with electronic health record data, from ~85,000 NHS patients in England affected by a rare disease or cancer, as well as the parents of some of the rare disease patients [[17]](https://www.zotero.org/google-docs/?6azY54). Recruitment of participants to the 100,000 Genomes Project was completed in 2018. All participants gave informed consent.

Around 1.7% of the 100KGP cohort are also participants in UKB, but the conditions of use preclude any attempt to identify the overlapping samples. Assuming that the overlapping samples are randomly distributed, this would suggest that ~1.2% of the 100KGP cohort could have been included in the GWAS training data for the Enhanced PRS models (but there would be no overlap with the training data for the Standard PRS models).

For our PRS performance evaluations, rare disease affected participants were excluded and from related pairs (up to 3rd degree), one randomly selected individual was included (KING[[5]](https://www.zotero.org/google-docs/?VWanyX) threshold = 0.0442), resulting in a sample of 40,001 for analysis (35,123, 3,262, 1,209, 353 and 54 individuals with European, South Asian, African, East Asian and Native/Indigenous American ancestries, respectively). Germline Whole Genome Sequence (WGS) data were filtered to the GWAS analysis variant list. To fill in gaps in the variant list, genotypes were phased (Eagle v2.4.1 [[18]](https://www.zotero.org/google-docs/?7LSotv)) and imputed (Minimac3 [[19]](https://www.zotero.org/google-docs/?LfjnPw)) using the 1000 Genomes Project reference panel. This set of common variant genotypes has been made available as a shared resource in the Genomics England research environment.

Hospital Episode Statistics (HES) and cancer registry data were used to identify disease cases and controls using ICD-10 codes (see S1 Table). For cancer traits, individuals that received a differing cancer diagnosis prior to, or contemporaneously to, the main diagnosis were removed to ensure the cancer diagnosis was not a result of comorbid metastasis. Controls were selected exclusively from the participant pool of the unaffected rare disease relatives. For non-cancer traits, individuals that received a cancer diagnosis prior to the main diagnosis were removed to avoid disease development due to cancer treatment effects. Controls were selected from both rare disease arm (unaffected relatives) and cancer arm. Twelve disease traits were evaluated (for sample size information, see S2 Table).

To enable fair PRS performance comparisons of 100KGP with UKB, software was imported into the 100KGP research environment to apply the same approach to genetic ancestry estimation, PRS calculation and ancestry-based centering and variance normalisation.

## Polygenic Risk Score Reporting Standards (Wand et al 2021)

Wand et al [[20]](https://www.zotero.org/google-docs/?j8k9SP) (their Table 1) provide recommendations on the reporting of PRS development and evaluation studies. Here, we provide notes on the correspondence between these recommendations and our study, using paragraph headings corresponding to Table 1 of Wand et al.

Background. Our study is a validation study performed on the previously released UK Biobank PRS Release (for 53 disease and quantitative traits), as well as on a set of PRSs calculated using the same PRS algorithms on a subset of traits in the 100,000 Genomes Project (for 12 diseases). The scores are available to UK Biobank and 100,000 Genomes Project researchers via the usual application processes. The risk models (PRS algorithms) used to generate these scores were constructed for the purpose of academic research within UK Biobank and the 100,000 Genomes Project. These research uses include biological, epidemiological and modelling investigations. The modelling investigations may include the modelling of clinical interventions.

Study population and data. The study design and recruitment, participant demographics and clinical characteristics, ancestry, genetic data, non-genetic variables, outcome of interest and treatment missing data within UK Biobank and 100,000 Genomes Project are described in other sections of the Supplementary Information, and in additional references provided herein.

**Risk model development and application**. This is a PRS validation study only. See Thompson et al [[4]](https://www.zotero.org/google-docs/?ACn47F) for additional details on risk model development.

**Risk model evaluation**. We provide details of PRS distributions in S11 Table. Metrics for risk model predictive ability and discrimination are provided in various tables and figures, and described elsewhere in the Supplementary Information. This study evaluated standardised PRS scores and did not evaluate any integrated risk or absolute risk scores, thus no calibration metrics are presented. Performance metrics are only presented in validation samples, in order to avoid inflation arising from overfitting. Subgroup analyses, together with subgroup counts, are provided by genetic ancestry in S4 and S4 Tables, and by sex in S8 Table.

**Limitations and clinical implications**. The incremental performance of the PRS scores is assessed by comparison with published PRSs (S3-S5 Tables). Limitations and generalizability are addressed in the Discussion. The PRS scores in UK Biobank and 100,000 Genomes Project are intended for research only. Clinical implications are discussed in the main text.

**Data transparency and availability**. The PRS scores and performance evaluation software are available to UK Biobank and 100,000 Genomes Project researchers via the usual application processes. The PRS algorithms used to generate the PRS scores are proprietary. In consequence, the PRS scores are provided as-is, and the application of the PRS algorithms to other cohorts and datasets is controlled by Genomics. The aim of these PRS releases is to provide researchers with high performance PRS scores in UK Biobank and 100,000 Genomes Project for use in their research. To date (June 2024), since the UK Biobank PRS Release was first made available in June 2022, more than 60 publications have made use of the UK Biobank PRS Release. The aim of this study is to provide researchers with performance information in support of such use.

# Supplementary references

[1. Sudlow C, Gallacher J, Allen N, Beral V, Burton P, Danesh J, et al. UK Biobank: an open access resource for identifying the causes of a wide range of complex diseases of middle and old age. PLoS Med. 2015;12: e1001779. doi:10.1371/journal.pmed.1001779](https://www.zotero.org/google-docs/?rL1vSA)

[2. Bycroft C, Freeman C, Petkova D, Band G, Elliott LT, Sharp K, et al. The UK Biobank resource with deep phenotyping and genomic data. Nature. 2018;562: 203–209. doi:10.1038/s41586-018-0579-z](https://www.zotero.org/google-docs/?rL1vSA)

[3. UK Biobank Coordinating Centre. ACCESS PROCEDURES: Application and review procedures for access to the UK Biobank Resource. 2011.](https://www.zotero.org/google-docs/?rL1vSA)

[4. Thompson DJ, Wells D, Selzam S, Peneva I, Moore R, Sharp K, et al. UK Biobank release and systematic evaluation of optimised polygenic risk scores for 53 diseases and quantitative traits. medRxiv; 2022. doi:10.1101/2022.06.16.22276246](https://www.zotero.org/google-docs/?rL1vSA)

[5. Manichaikul A, Mychaleckyj JC, Rich SS, Daly K, Sale M, Chen W-M. Robust relationship inference in genome-wide association studies. Bioinformatics. 2010;26: 2867–2873. doi:10.1093/bioinformatics/btq559](https://www.zotero.org/google-docs/?rL1vSA)

[6. Mathieson I, Scally A. What is ancestry? PLoS Genet. 2020;16: e1008624. doi:10.1371/journal.pgen.1008624](https://www.zotero.org/google-docs/?rL1vSA)

[7. Price AL, Patterson NJ, Plenge RM, Weinblatt ME, Shadick NA, Reich D. Principal components analysis corrects for stratification in genome-wide association studies. Nat Genet. 2006;38: 904–909. doi:10.1038/ng1847](https://www.zotero.org/google-docs/?rL1vSA)

[8. The 1000 Genomes Project Consortium, Auton A, Abecasis GR, Altshuler DM, Durbin RM, Abecasis GR, et al. A global reference for human genetic variation. Nature. 2015;526: 68–74. doi:10.1038/nature15393](https://www.zotero.org/google-docs/?rL1vSA)

[9. Sun X, Xu W. Fast Implementation of DeLong’s Algorithm for Comparing the Areas Under Correlated Receiver Operating Characteristic Curves. IEEE Signal Process Lett. 2014;21: 1389–1393. doi:10.1109/LSP.2014.2337313](https://www.zotero.org/google-docs/?rL1vSA)

[10. Gail MH. Twenty-five Years of Breast Cancer Risk Models and Their Applications. JNCI J Natl Cancer Inst. 2015;107: djv042. doi:10.1093/jnci/djv042](https://www.zotero.org/google-docs/?rL1vSA)

[11. Karczewski KJ, Francioli LC, Tiao G, Cummings BB, Alföldi J, Wang Q, et al. The mutational constraint spectrum quantified from variation in 141,456 humans. Nature. 2020;581: 434–443. doi:10.1038/s41586-020-2308-7](https://www.zotero.org/google-docs/?rL1vSA)

[12. Richards S, Aziz N, Bale S, Bick D, Das S, Gastier-Foster J, et al. Standards and guidelines for the interpretation of sequence variants: a joint consensus recommendation of the American College of Medical Genetics and Genomics and the Association for Molecular Pathology. Genet Med. 2015;17: 405–423. doi:10.1038/gim.2015.30](https://www.zotero.org/google-docs/?rL1vSA)

[13. Marduel M, Ouguerram K, Serre V, Bonnefont-Rousselot D, Marques-Pinheiro A, Erik Berge K, et al. Description of a Large Family with Autosomal Dominant Hypercholesterolemia Associated with the APOE p.Leu167del Mutation. Hum Mutat. 2013;34: 83–87. doi:10.1002/humu.22215](https://www.zotero.org/google-docs/?rL1vSA)

[14. Fahed AC, Wang M, Homburger JR, Patel AP, Bick AG, Neben CL, et al. Polygenic background modifies penetrance of monogenic variants for tier 1 genomic conditions. Nat Commun. 2020;11: 3635. doi:10.1038/s41467-020-17374-3](https://www.zotero.org/google-docs/?rL1vSA)

[15. Lévesque LE, Hanley JA, Kezouh A, Suissa S. Problem of immortal time bias in cohort studies: example using statins for preventing progression of diabetes. BMJ. 2010;340: b5087. doi:10.1136/bmj.b5087](https://www.zotero.org/google-docs/?rL1vSA)

[16. Therneau T, Atkinson E. Concordance (R survival package vignette). 2020.](https://www.zotero.org/google-docs/?rL1vSA)

[17. Genomics England. The 100,000 Genomes Project protocol. 2017. Available: https://figshare.com/articles/journal_contribution/GenomicEnglandProtocol_pdf/4530893/4](https://www.zotero.org/google-docs/?rL1vSA)

[18. Loh P-R, Danecek P, Palamara PF, Fuchsberger C, A Reshef Y, K Finucane H, et al. Reference-based phasing using the Haplotype Reference Consortium panel. Nat Genet. 2016;48: 1443–1448. doi:10.1038/ng.3679](https://www.zotero.org/google-docs/?rL1vSA)

[19. Das S, Forer L, Schönherr S, Sidore C, Locke AE, Kwong A, et al. Next-generation genotype imputation service and methods. Nat Genet. 2016;48: 1284–1287. doi:10.1038/ng.3656](https://www.zotero.org/google-docs/?rL1vSA)

[20. Wand H, Lambert SA, Tamburro C, Iacocca MA, O’Sullivan JW, Sillari C, et al. Improving reporting standards for polygenic scores in risk prediction studies. Nature. 2021;591: 211–219. doi:10.1038/s41586-021-03243-6](https://www.zotero.org/google-docs/?rL1vSA)
