# Supplementary figures and images for: A systematic evaluation of the performance and properties of the UK Biobank Polygenic Risk Score (PRS) Release
Source: PLoS One. 2024 Sep 18;19(9):e0307270. doi: 10.1371/journal.pone.0307270 (PMC11410272; doi:10.1371/journal.pone.0307270)

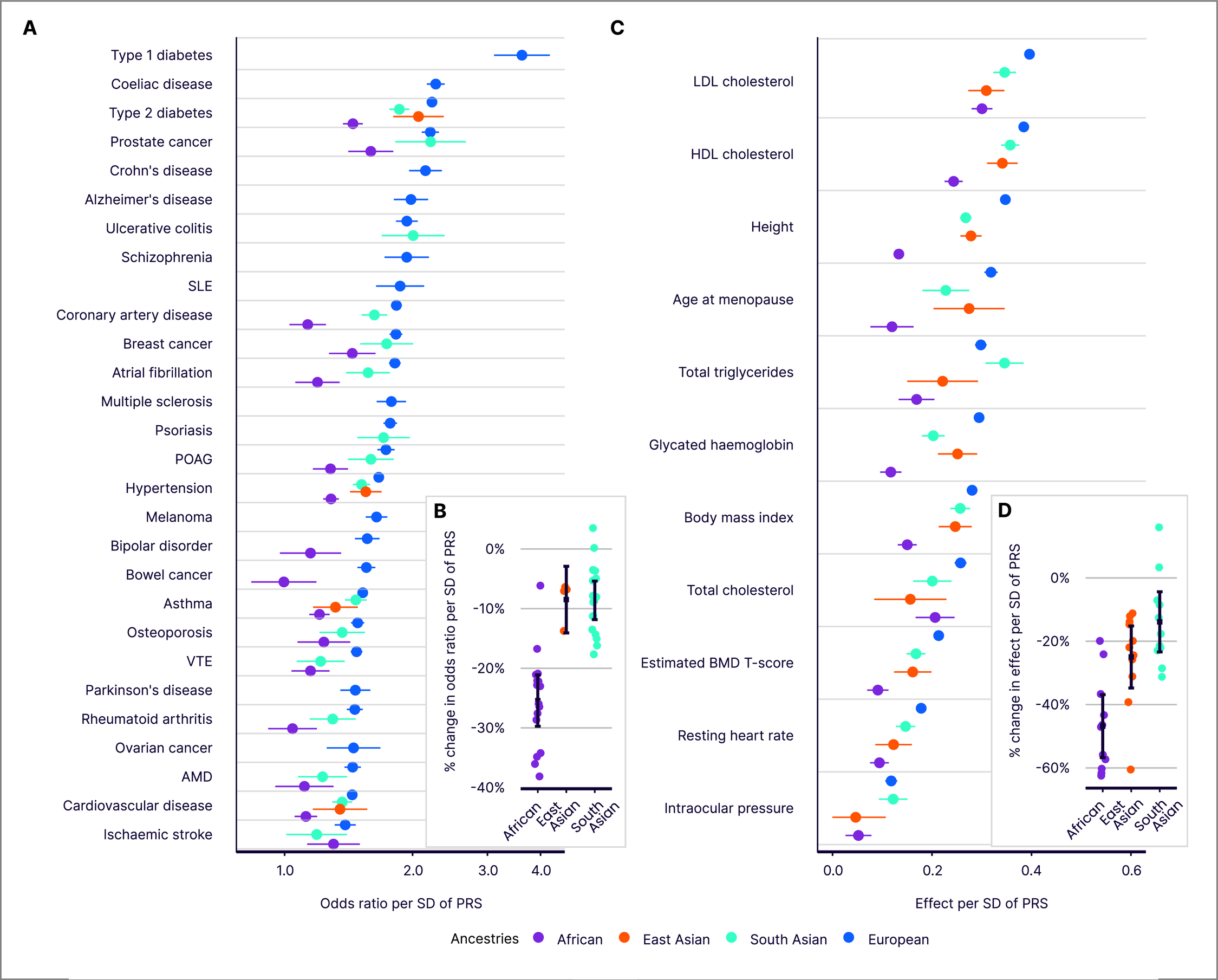

Supplement: S1 Fig — Performance (odds ratio, or effect on standardised quantitative trait, per SD of PRS, adjusting for age and sex), measured in the independent UKB Testing Subgroup, of the disease traits (A) and quantitative traits (C), stratified by genetically inferred ancestry. Results for non-European ancestries are shown if at least 100 cases are available for testing. Relative change in performance in non-European compared to European ancestries for disease traits (B) and quantitative traits (D). Odds ratios are shown on a log scale. Bars indicate 95% confidence intervals (CI). Refer to Fig 1 legend for disease and quantitative trait abbreviations. (TIF) [file pone.0307270.s013.tif]

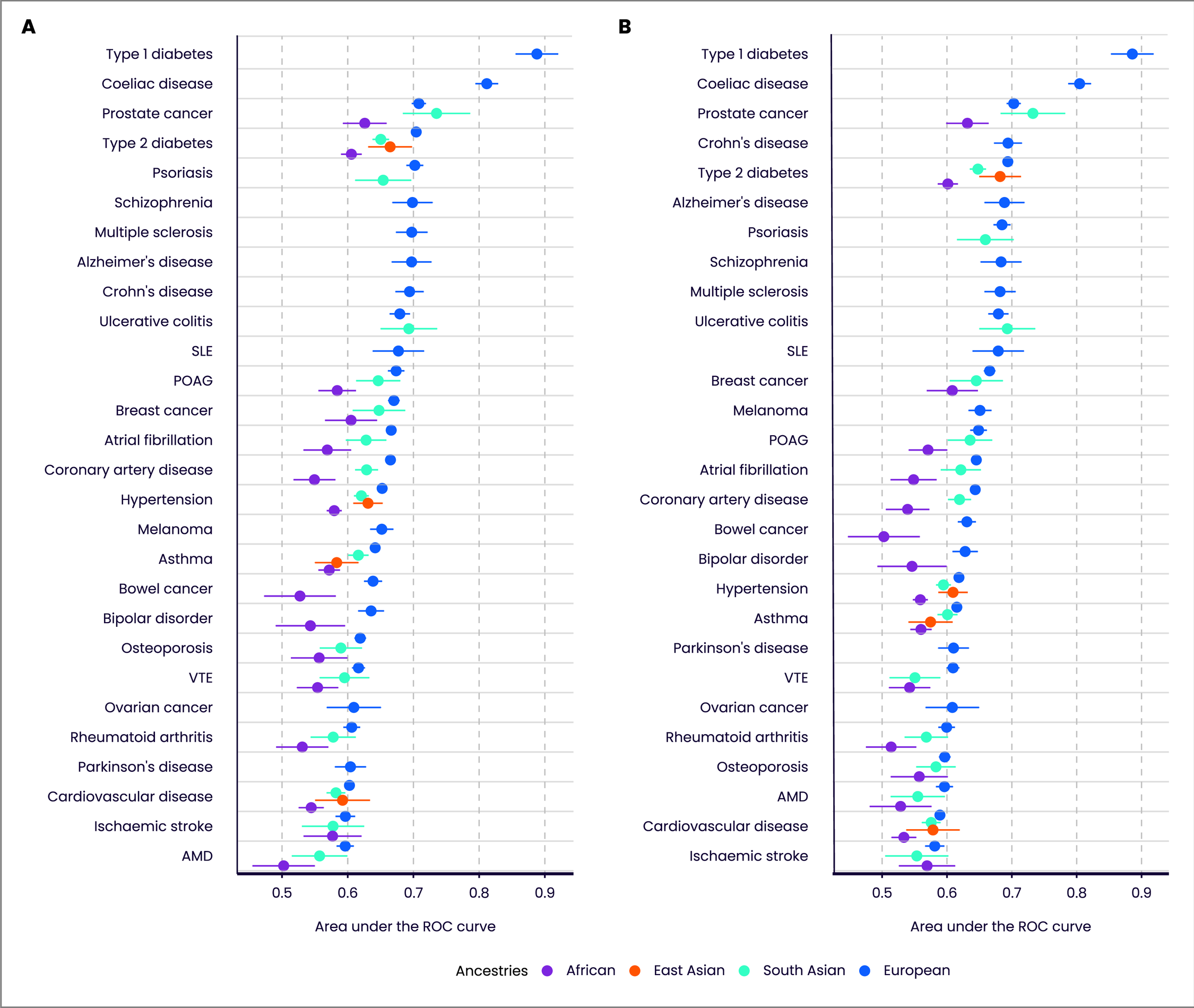

Supplement: S2 Fig — Performance (area under the receiver operating characteristic (ROC) curve, or AUC), measured in the independent UKB Testing Subgroup, of the disease traits in the Standard (A) and Enhanced (B) PRS sets, stratified by genetically inferred ancestry. Results for non-European ancestries are shown if at least 100 cases are available for testing. Bars indicate 95% confidence intervals (CI). Refer to Fig 1 legend for disease abbreviations. (TIF) [file pone.0307270.s014.tif]

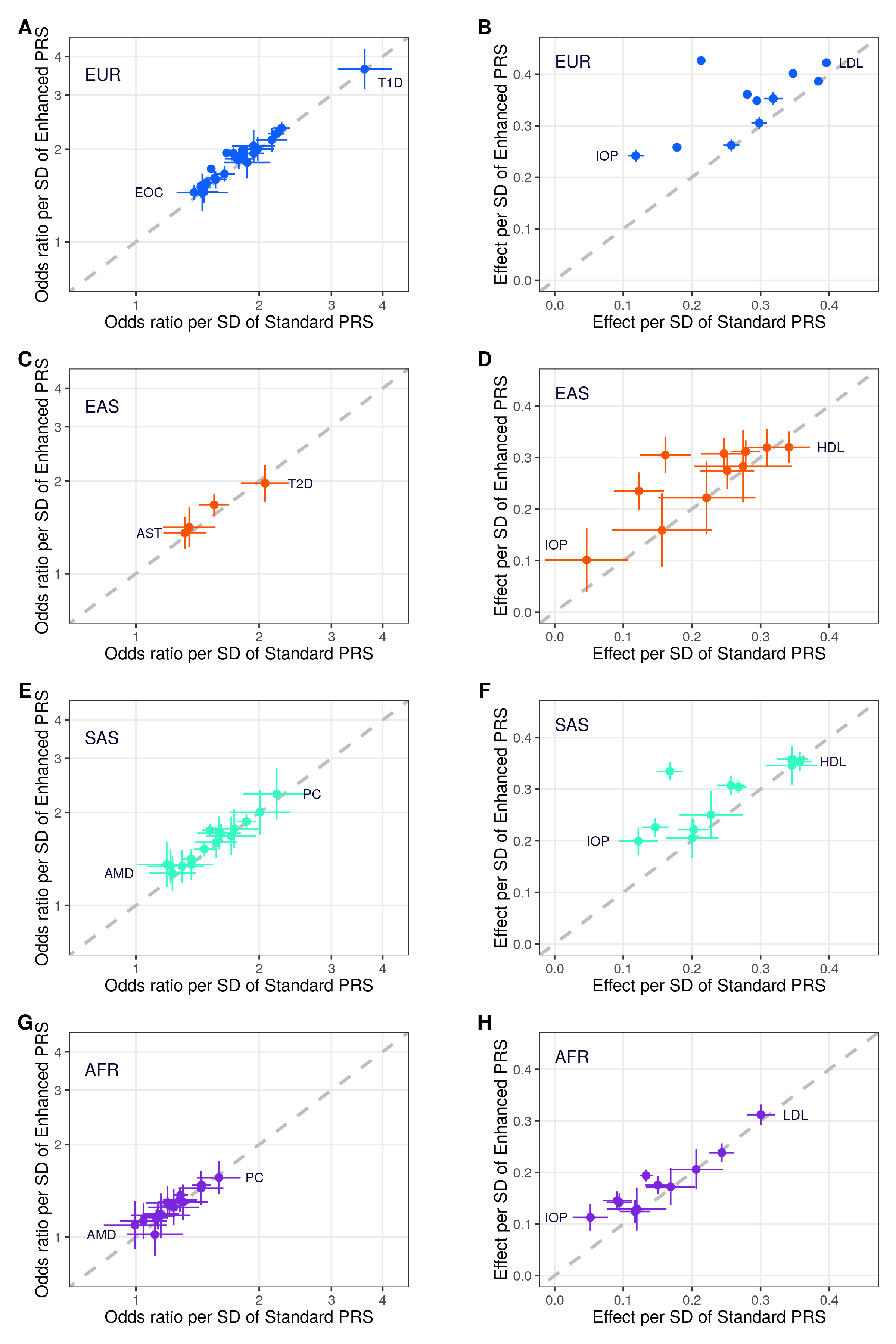

Supplement: S3 Fig — Performance (odds ratio, or effect on standardised quantitative trait, per SD of PRS, adjusting for age and sex), measured in the independent UKB Testing Subgroup, of the disease traits (A, C, E, G) and quantitative traits (B, D, F, H) in the Standard and Enhanced PRS sets in different ancestries. EUR = European ancestry group (A, B). EAS = East Asian ancestry group (C, D). SAS = South Asian ancestry group (E, F). AFR = Sub-Saharan African ancestry group (G, H). Bars indicate 95% confidence intervals (CI). Traits with highest and lowest Enhanced PRS performance are labelled. For trait codes see S1 Table. (PNG) [file pone.0307270.s015.png]

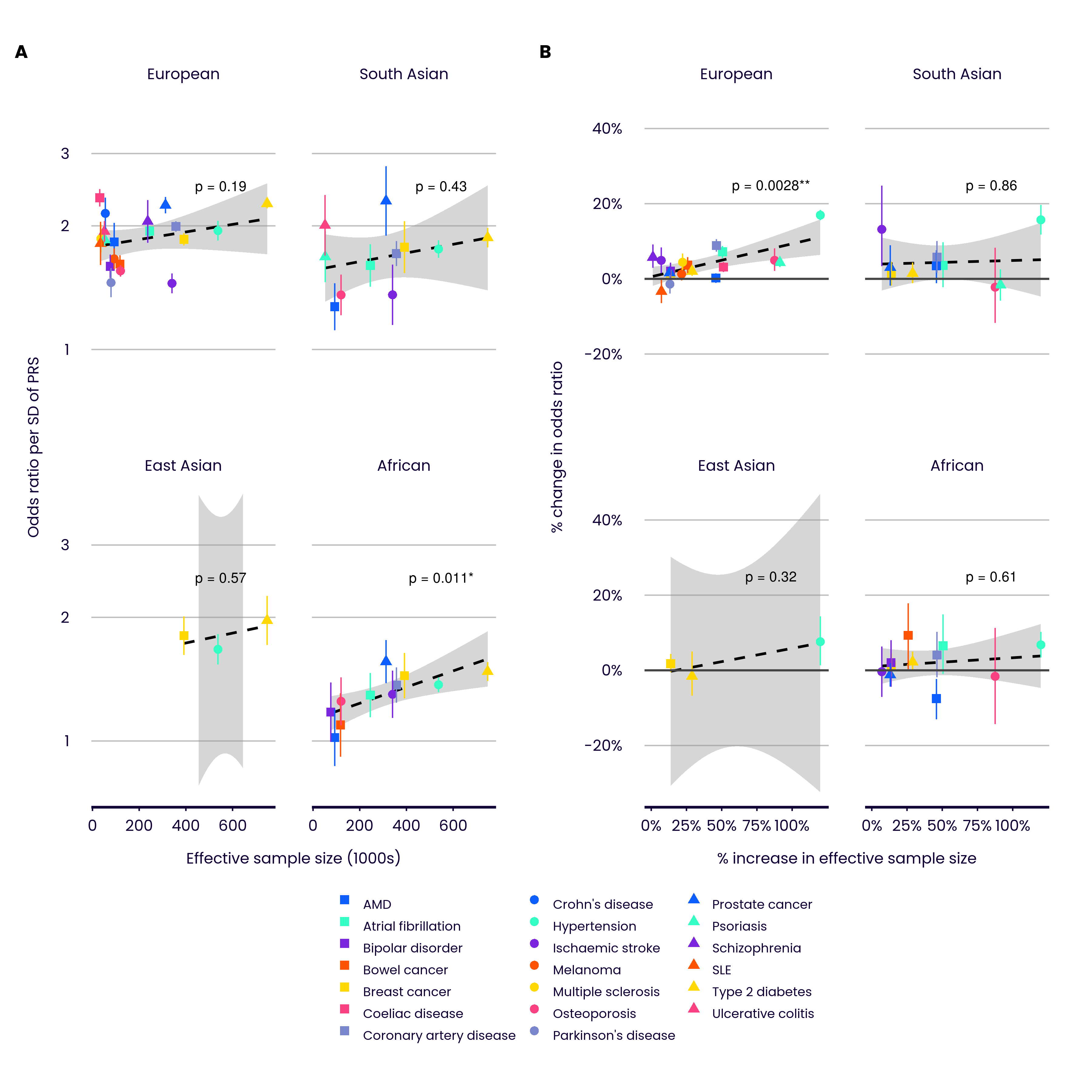

Supplement: S4 Fig — A Relationship between the ancestry-specific odds ratio and effective sample size (across all training GWASs) for the Enhanced PRS Set [20]. B Relationship between relative change in odds ratio and relative change in effective sample size, comparing the Enhanced to the Standard PRS Set [20]. Effective sample size is defined as 4∑jnjcj(1−cj), where nj and cj are respectively the total sample size and the proportion of cases for the jth constituent GWAS for a given trait. Only those diseases with non-overlapping samples in the constituent GWASs are displayed. Odds ratios are shown on a log scale. Bars indicate 95% confidence intervals. Dashed lines indicate linear regression slopes, with p-values and asterisks indicating the significance of the slope (* p<0.05, ** p<0.01, *** p<0.001). Refer to Fig 1 legend for disease abbreviations. (PNG) [file pone.0307270.s016.png]

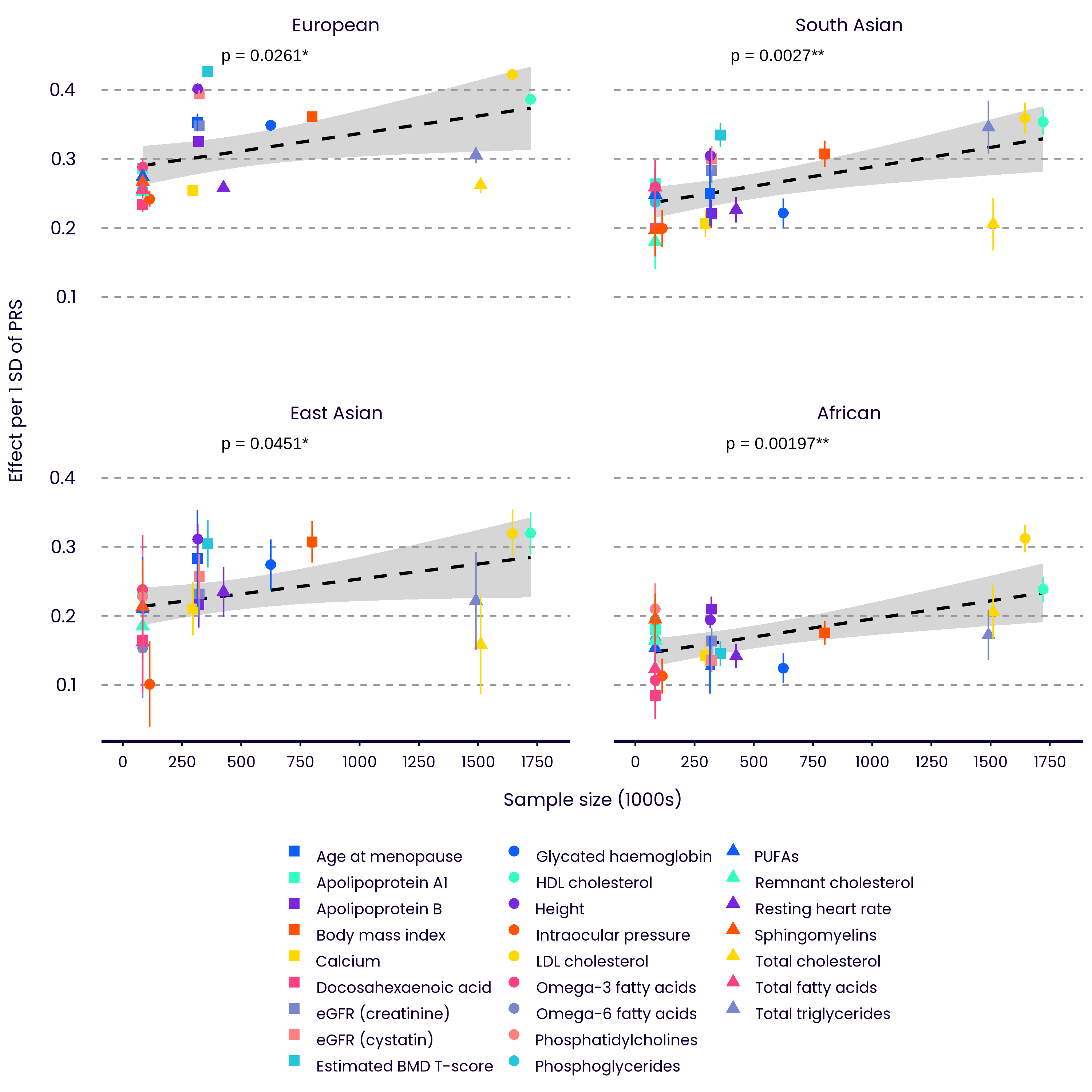

Supplement: S5 Fig — Relationship between the ancestry-specific effect on standardised quantitative trait, per SD of PRS, and sample size (across all GWASs) for the Enhanced PRS Set [20]. Only those traits with non-overlapping samples in the constituent GWASs are displayed. Bars indicate 95% confidence intervals. Dashed lines indicate linear regression slopes, with p-values and asterisks indicating the significance of the slope (* p<0.05, ** p<0.01, *** p<0.001). Refer to Fig 1 legend for quantitative trait abbreviations. (PNG) [file pone.0307270.s017.png]

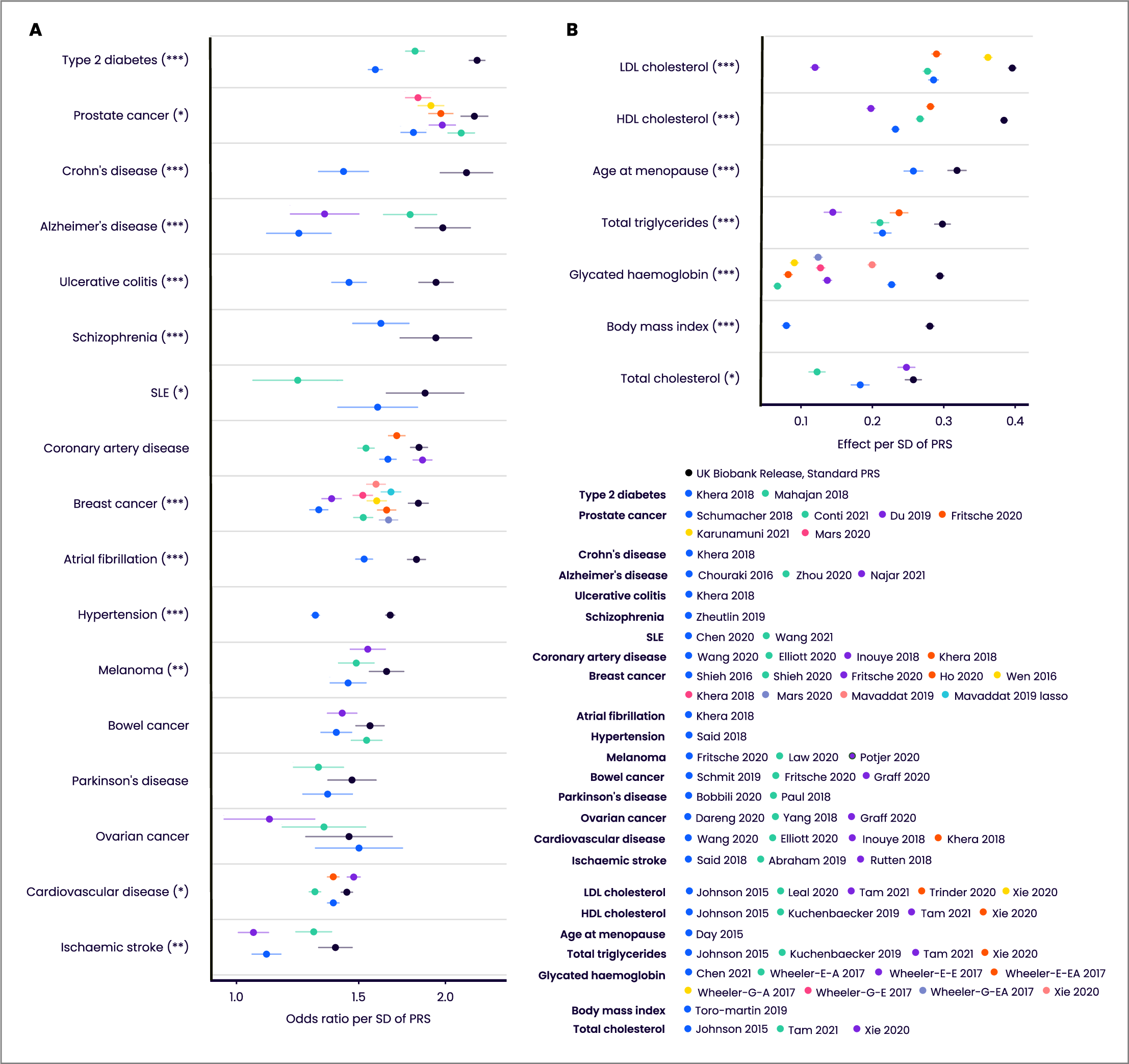

Supplement: S6 Fig — Performance (odds ratio, or effect on standardised quantitative trait, per SD of PRS, adjusting for age and sex) in the independent UKB Testing Subgroup (European ancestries) of the Standard PRS sets for disease traits (A) and quantitative traits (B), for those traits for which there are published PRS algorithms (citations provided in S6 Table). Odds ratios are shown on a log scale. Bars indicate 95% confidence intervals. Asterisks indicate significance level for difference in performance between the Standard PRS and the nearest comparator PRS (5000 bootstraps): * p<0.05, ** p<0.01, *** p<0.001. Wheeler-E-A, Wheeler-E-E and Wheeler-E-EA refer respectively to the African, European and East Asian ancestry versions of the Wheeler 2017 PRSs for glycated haemoglobin using erythrocytic variants. Wheeler-G-A, Wheeler-G-E and Wheeler-G-EA refer respectively to the African, European and East Asian ancestry versions of the Wheeler 2017 PRSs for glycated haemoglobin using glycemic variants. Refer to Fig 1 legend for disease and quantitative trait abbreviations. (TIF) [file pone.0307270.s018.tif]

A

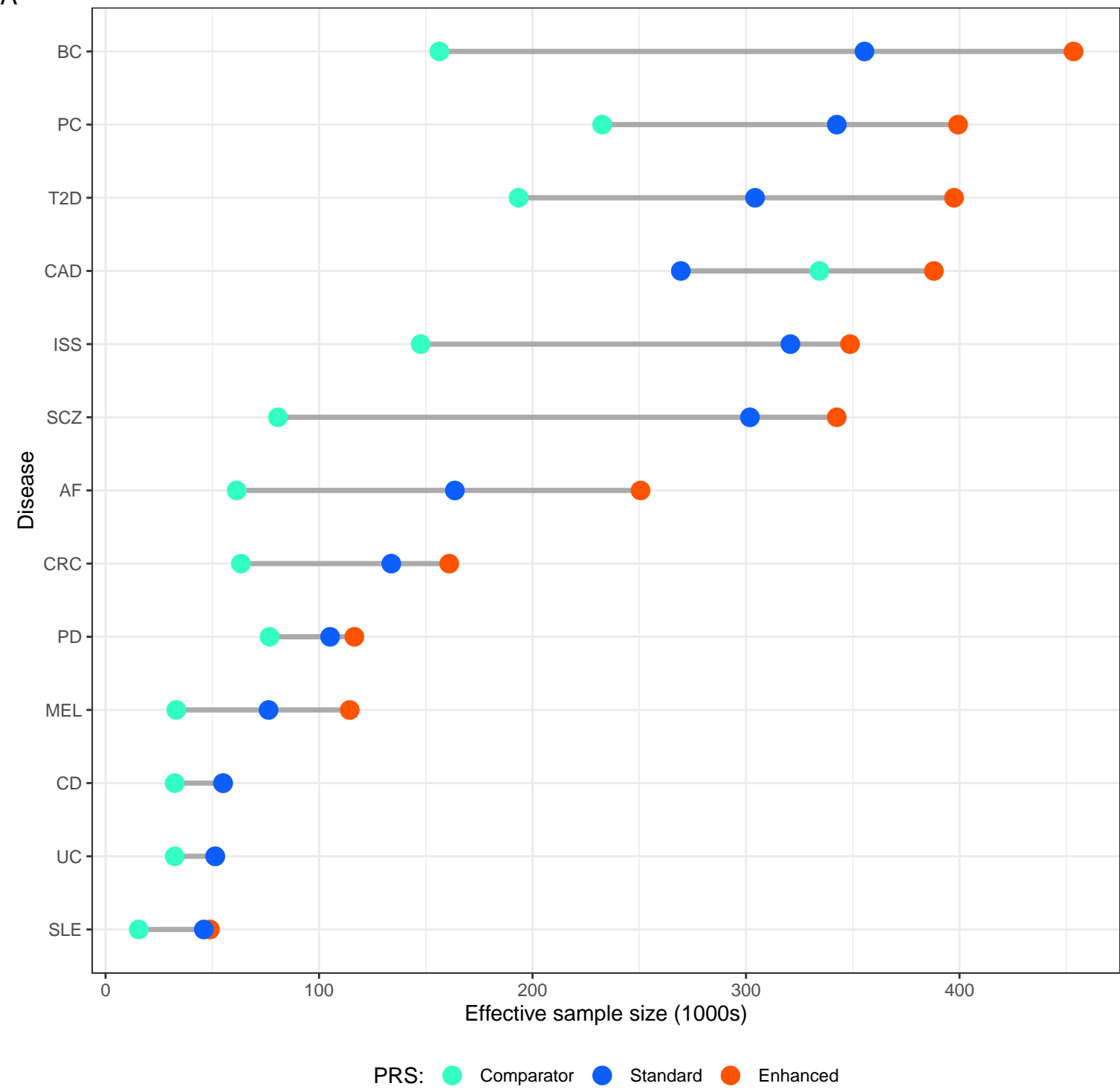

B

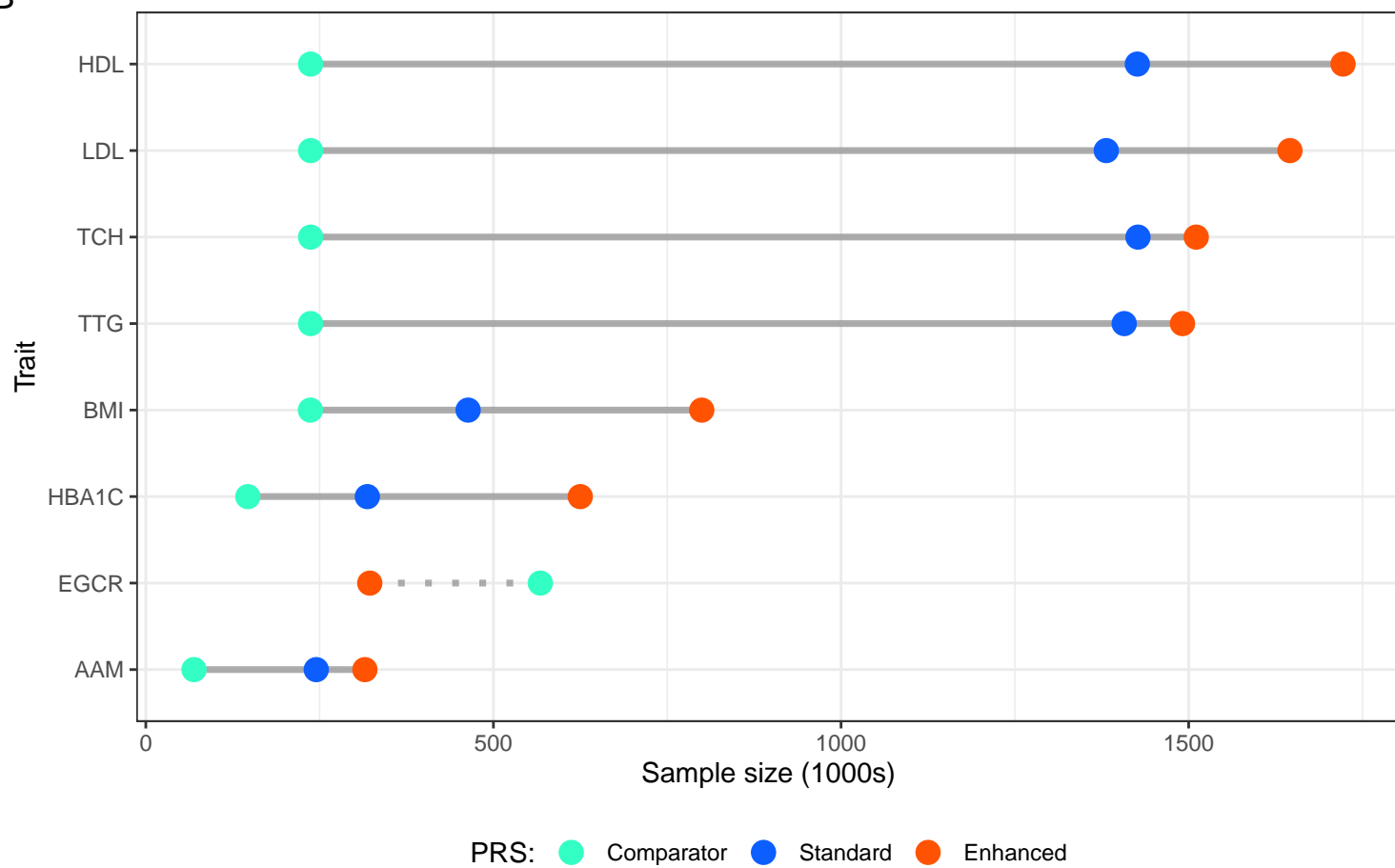

Supplement: S7 Fig — Comparison of training data effective sample sizes (A) and total samples (B) for disease and quantitative traits, among Comparator, Standard and Enhanced PRS. Total training sample sizes for the Enhanced PRS, Standard PRS, and the best-performing comparator PRS, for disease traits (A) and quantitative traits (B) [20]. For disease traits, the x-axis is the effective sample size, defined as 4 / ((1/n0) + (1/n1)), where n0 is the total number of controls, and n1 is the total number of cases. Where training data came from meta-analysis of multiple GWASs, the total numbers are used, as it was not always possible to obtain accurate per-GWAS numbers for the PGS Catalog PRSs. A dashed connecting line is used where the comparator PRS sample size is larger than the Enhanced PRS sample size. Only those traits for which at least one comparator PRS was available are displayed. Traits with overlapping samples in the Standard/Enhanced PRS training are excluded. In addition, ischaemic stroke is not shown, because the best comparator PRS (Abraham et al 2019, doi: 10.1038/s41467-019-13848-1) was trained using a complex combination of PRSs for 19 different diseases and quantitative traits; hypertension is also excluded because the only comparator PRS (Said et al 2018, doi: 10.1001/jamacardio.2018.1717) used a combination of disease and quantitative trait data. Refer to Fig 1 legend for disease and quantitative trait abbreviations. (PDF) [file pone.0307270.s019.pdf]

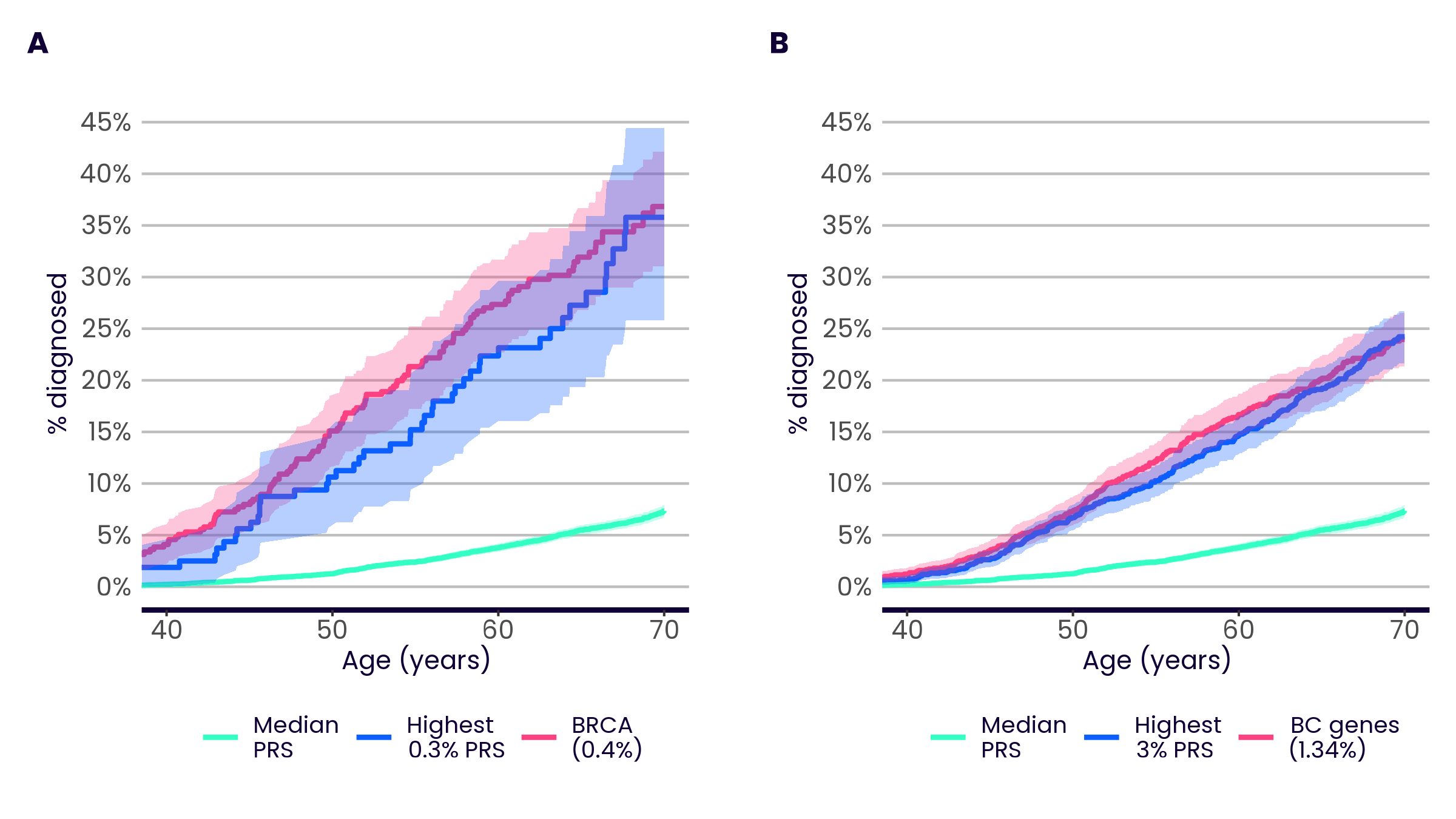

Supplement: S8 Fig — Cumulative incidence of breast cancer for carriers of high-risk mutations in breast cancer associated genes (red), compared to individuals in the top fraction of the PRS distribution (blue) corresponding to equivalent risk, and the median 40–60% of the PRS (green). Carrier risks are evaluated in UKB women with European ancestries for whom exome sequencing data are available. PRS risks are evaluated in the UKB Testing Subgroup (European ancestries, female). A, Incidence of breast cancer in BRCA1+BRCA2 loss-of-function variant carriers (0.4% of evaluation group) vs top 0.3% of breast cancer Enhanced PRS. B, Incidence of breast cancer in combined BRCA1+BRCA2+ATM+CHEK2+PALB2 loss-of-function variant carriers (1.34% of evaluation group) vs top 3% of breast cancer Enhanced PRS. Sample size details are provided in S7 Table. Shaded areas indicate 95% CI. (TIFF) [file pone.0307270.s020.tiff]

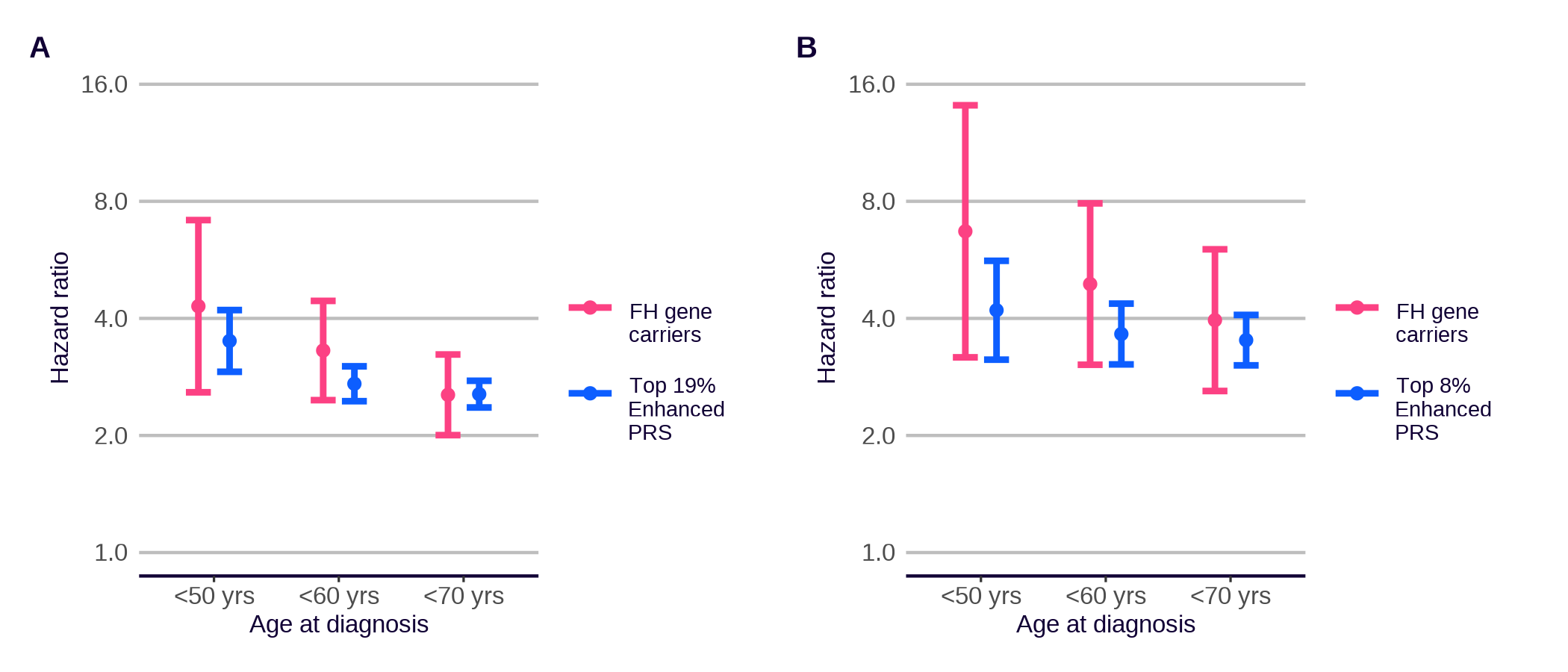

Supplement: S9 Fig — The relative influence on CAD risk (hazard ratio) of FH carrier vs non-carrier status (pink), and of high vs median (40–60%) Enhanced CAD PRS status (blue). A Analyses for the top 19% of the Enhanced CAD PRS. Carrier risks are evaluated in UKB individuals with European ancestries for whom whole exome sequencing data were available; PRS risks are evaluated in the UKB Testing Subgroup (European ancestries). B Analyses for the top 8% of the Enhanced CAD PRS. Carrier and PRS risks are evaluated in their respective Panel A groups, additionally restricted to those with primary care data linkage and no recorded statin prescription prior to CAD event. Sample size details are provided in S7 Table. Bars represent 95% CI. (TIFF) [file pone.0307270.s021.tiff]

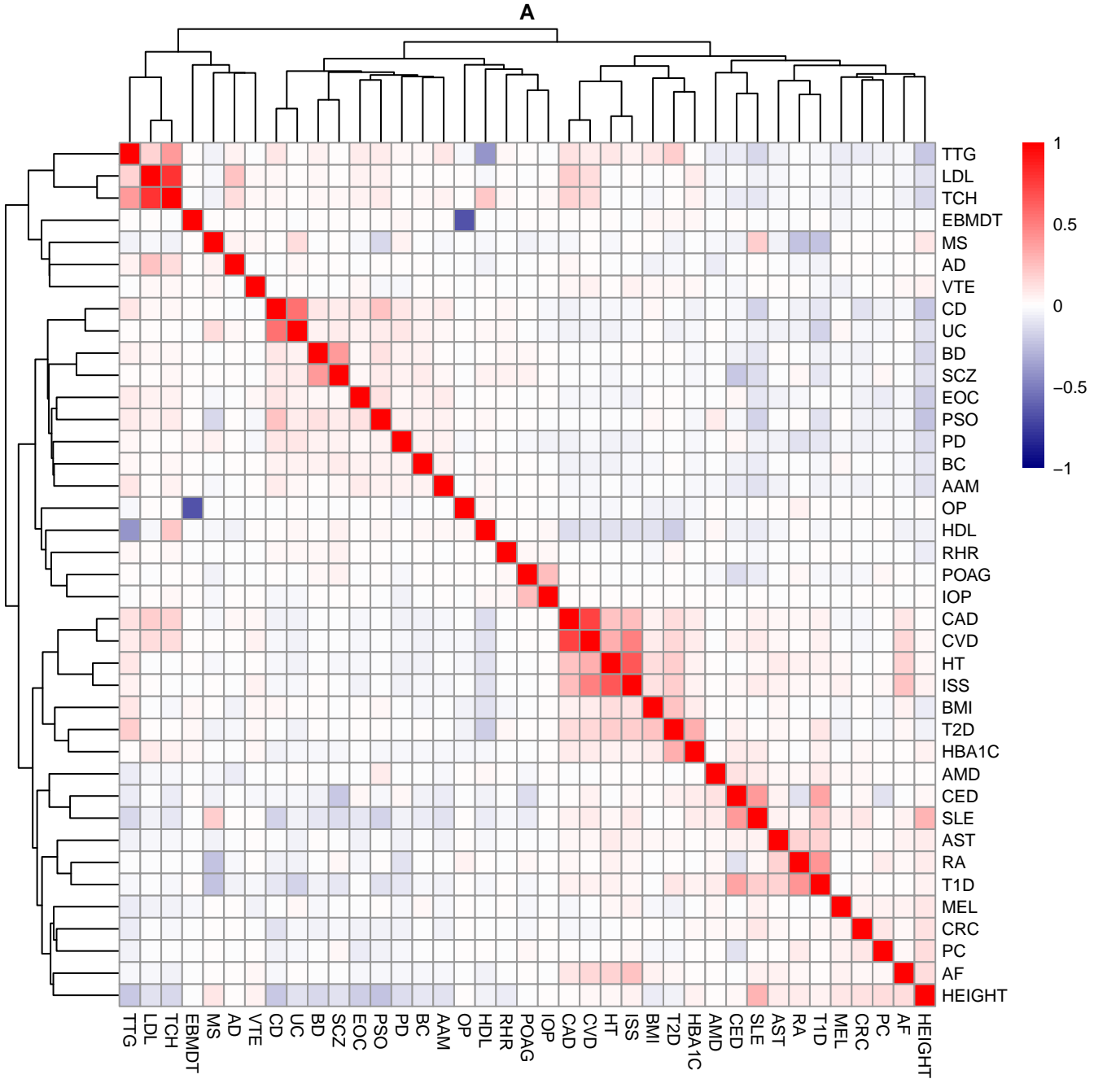

**B**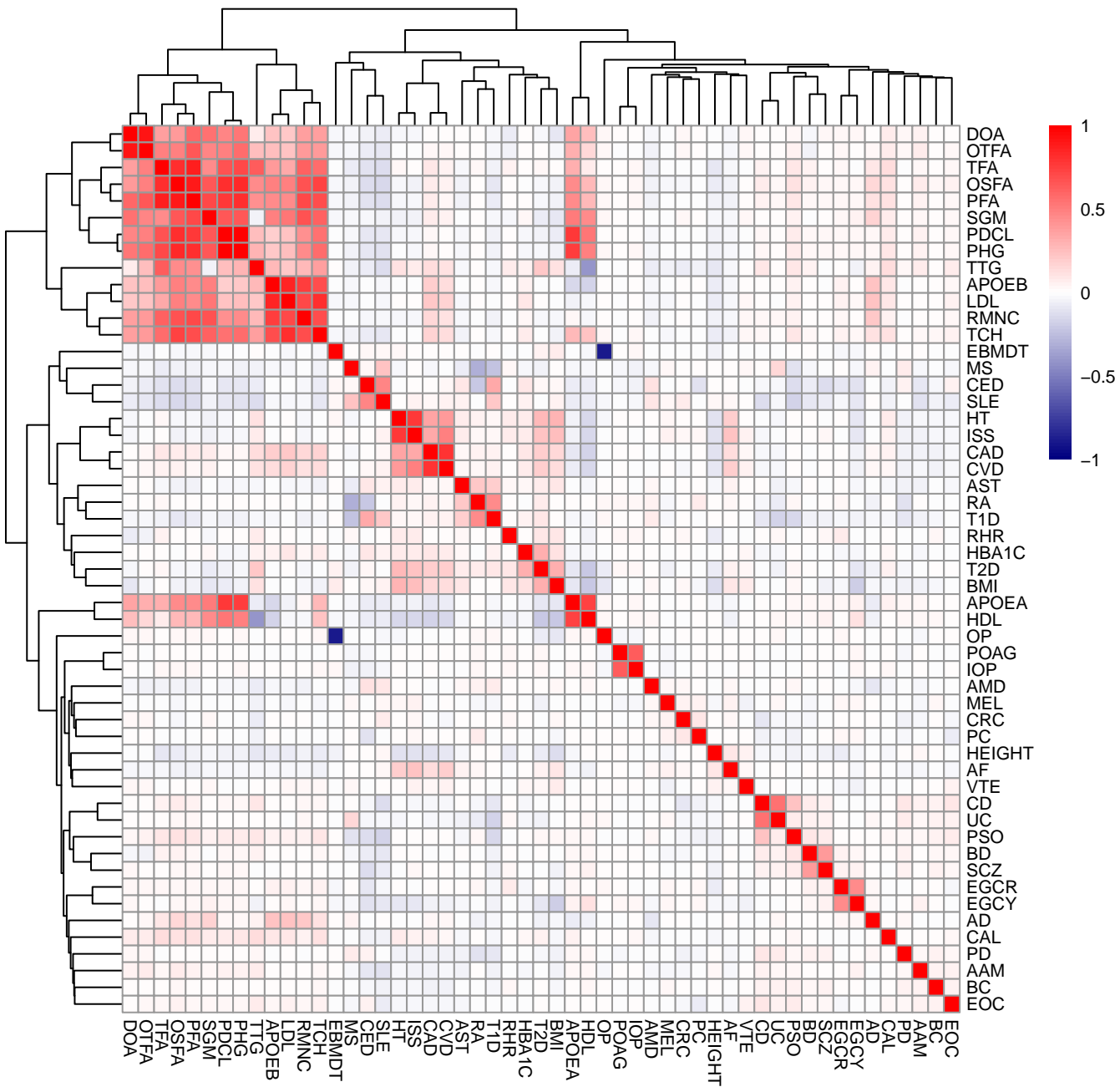

Supplement: S12 Fig — Correlations (calculated from individuals with European ancestries in the UKB Testing Subgroup) among diseases and quantitative traits for the Standard Set (A) and Enhanced Set (B), ordered according to a hierarchical clustering dendrogram (complete linkage on Euclidean distance, see hclust() function in R). See S1 Table for trait code mappings. (PDF) [file pone.0307270.s024.pdf]

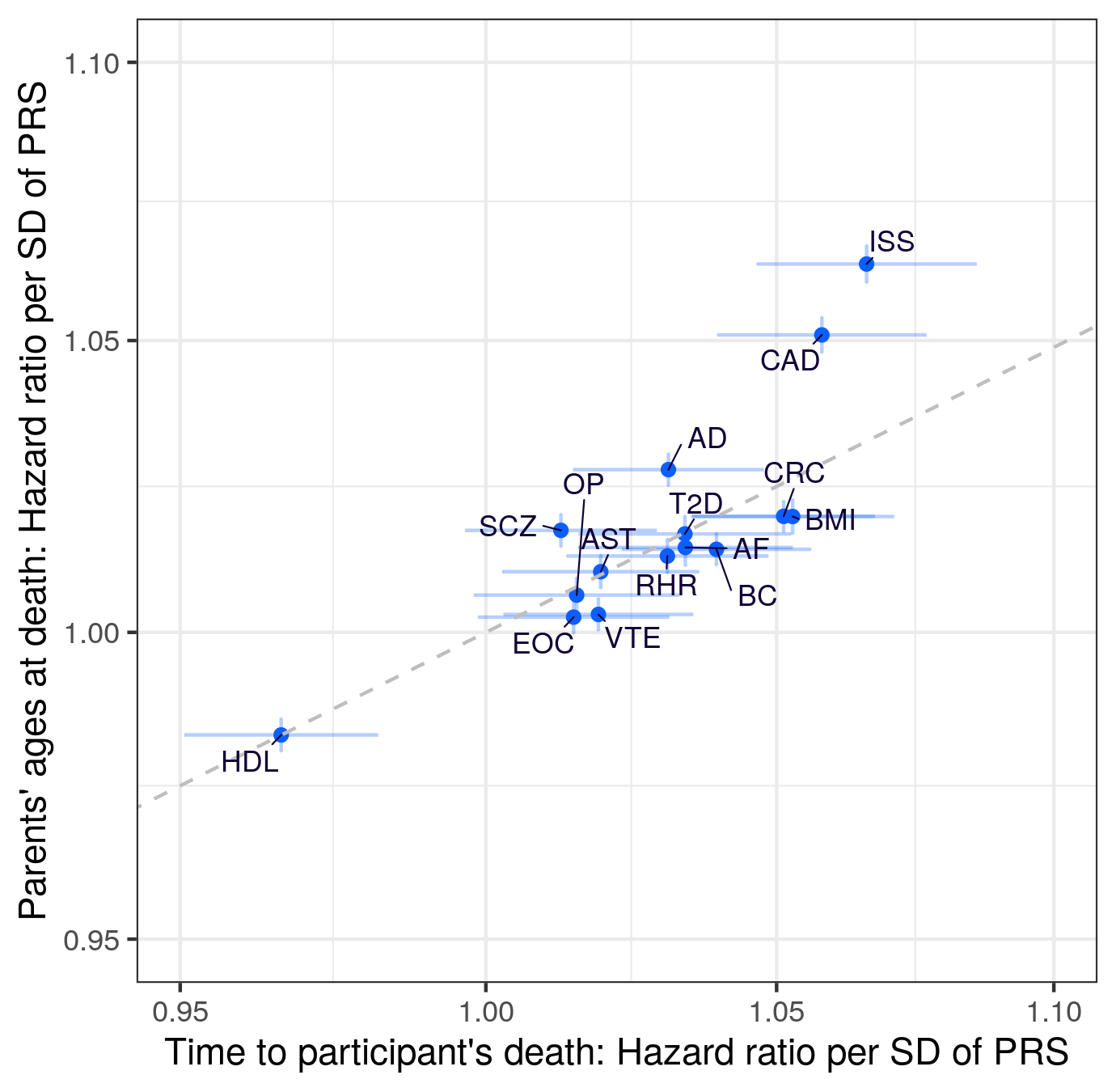

Supplement: S13 Fig — Traits are shown if selected both by stepwise regression of participant’s time-to-death from first assessment and also by stepwise regression of their parents’ age at death (maternal and paternal data entered as separate observations). Hazard ratios shown on a log scale. See S1 Table for trait code mappings. Dashed line shows the expected parent:offspring log(hazard ratio) ratio of 1:2. A natural explanation for the larger than expected effect of CAD and ISS PRSs on parental mortality is that these diseases were bigger killers in the past, and so made up a larger proportion of all-cause mortality. (PNG) [file pone.0307270.s025.png]
