# Supplementary material for: A systematic evaluation of the performance and properties of the UK Biobank Polygenic Risk Score (PRS) Release
Source: PLoS One. 2024 Sep 18;19(9):e0307270. doi: 10.1371/journal.pone.0307270 (PMC11410272; doi:10.1371/journal.pone.0307270)
Supplement: S10 Fig — Cumulative incidence plots are shown for each disease and each ancestry group in the UKB Testing Subgroup, provided more than 40 cases are available (the number of cases is printed otherwise), with separate curves for the highest 3% (red), lowest 3% (blue), and median 40–60% (green) of the PRS distribution. A. Alzheimer’s disease (AD). B. Atrial fibrillation (AF). C. Age-related macular degeneration (AMD). D. Asthma (AST). E. Breast cancer (BC), F. Bipolar disorder (BD), G. Coronary artery disease (CAD). H. Crohn’s disease (CD). I. Coeliac disease (CED). J. Bowel cancer (CRC). K. Cardiovascular disease (CVD), L. Epithelial ovarian cancer (EOC). M. Hypertension (HT). N. Ischaemic stroke (ISS). O. Melanoma (MEL). P. Multiple sclerosis (MS). Q. Osteoporosis (OP). R. Prostate cancer (PC). S. Parkinson’s disease (PD). T. Primary open angle glaucoma (POAG). U. Psoriasis (PSO). V. Rheumatoid arthritis (RA). W. Schizophrenia (SCZ). X. Systemic lupus erythematosus (SLE). Y. Type 1 diabetes (T1D). Z. Type 2 diabetes (T2D). AA. Ulcerative colitis (UC). AB. Venous thromboembolic disease (VTE). EUR = European ancestry group. EAS = East Asian ancestry group. SAS = South Asian ancestry group. AFR = Sub-Saharan African ancestry group. Shaded areas indicate 95% CI. (PDF) [file pone.0307270.s022.pdf]

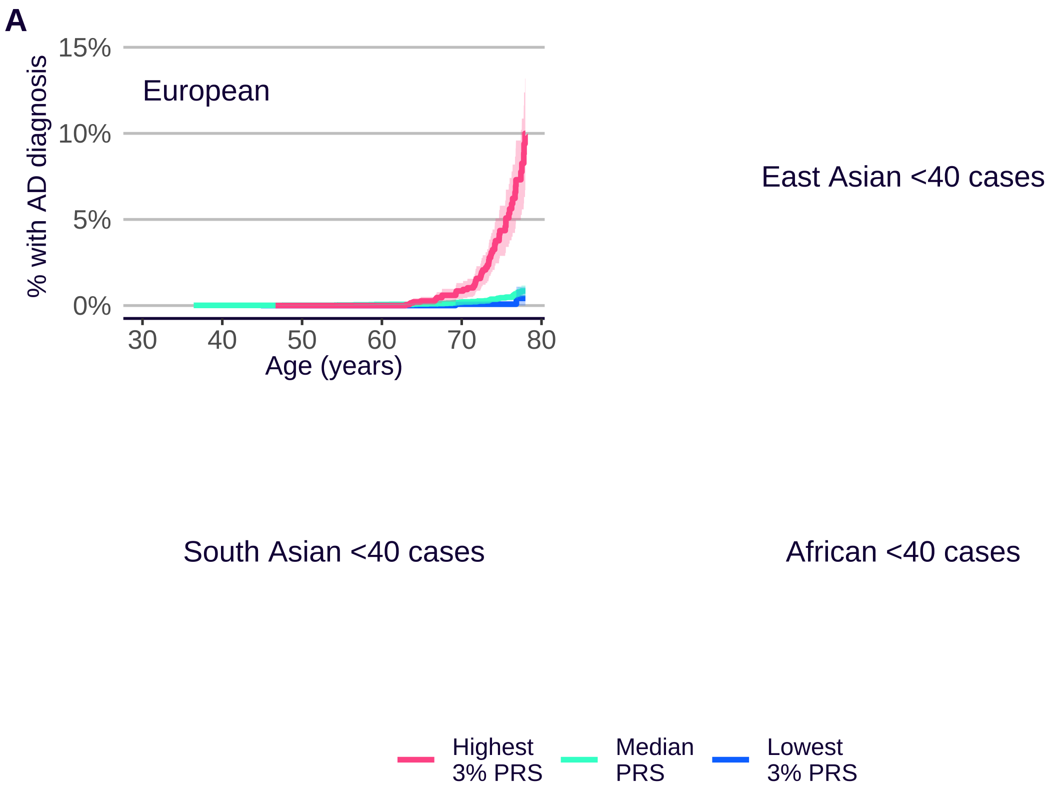
A
European
15%
10%
5%
0%
% with AD diagnosis
30
40
50
60
70
80
Age (years)
East Asian <40 cases
South Asian <40 cases
African <40 cases
Highest 3% PRS
Median PRS
Lowest 3% PRS

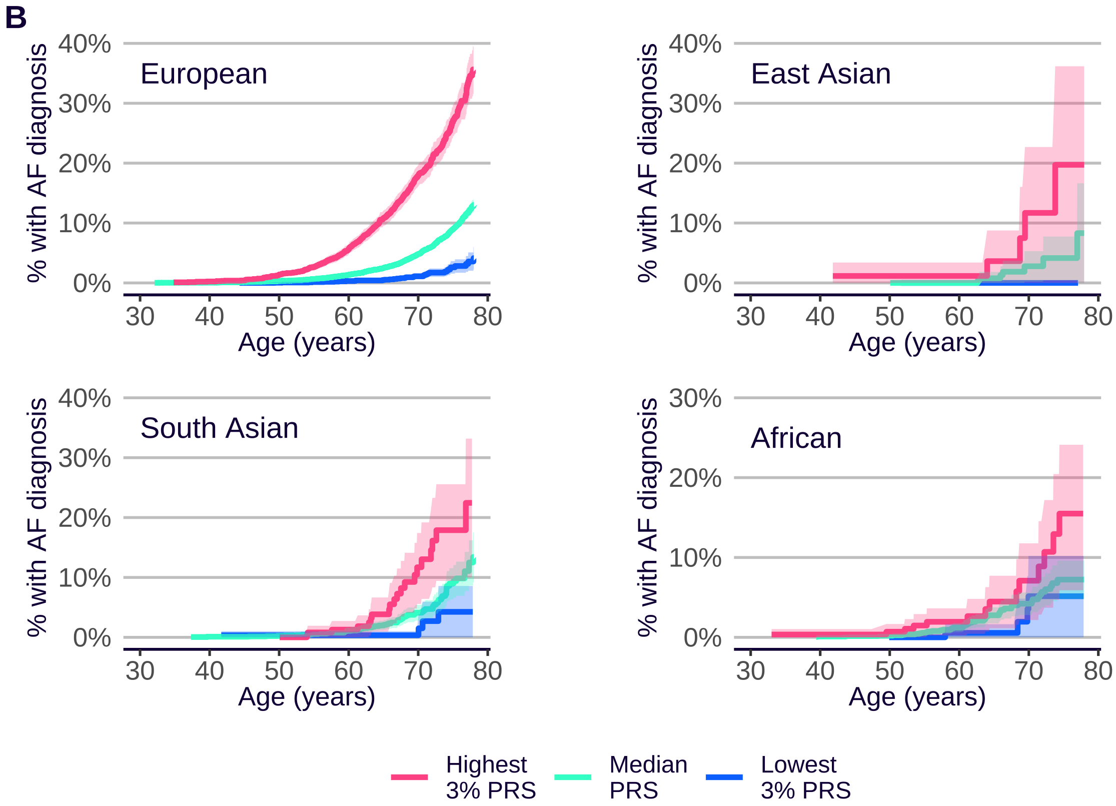
B
European
East Asian
South Asian
African
% with AF diagnosis
0%
10%
20%
30%
40%
Age (years)
30
40
50
60
70
80
Highest 3% PRS
Median PRS
Lowest 3% PRS

C

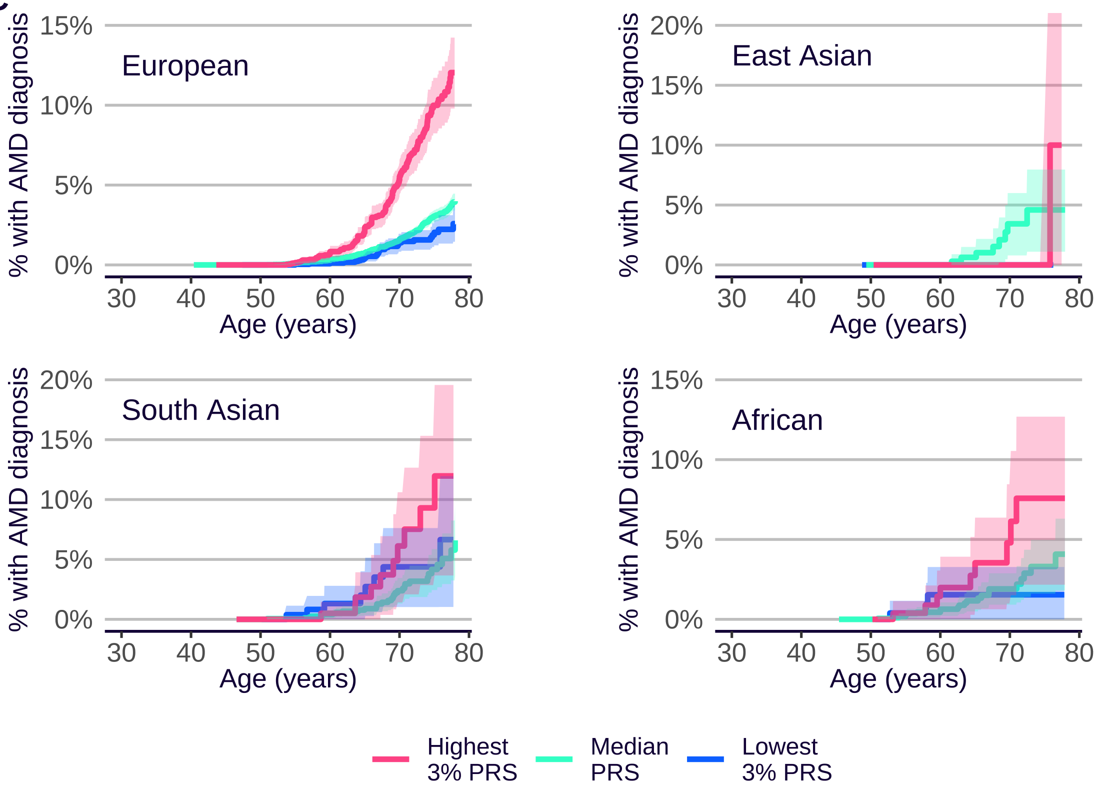
European
East Asian
South Asian
African
% with AMD diagnosis
Age (years)
0%
5%
10%
15%
20%
30
40
50
60
70
80
Highest 3% PRS
Median PRS
Lowest 3% PRS

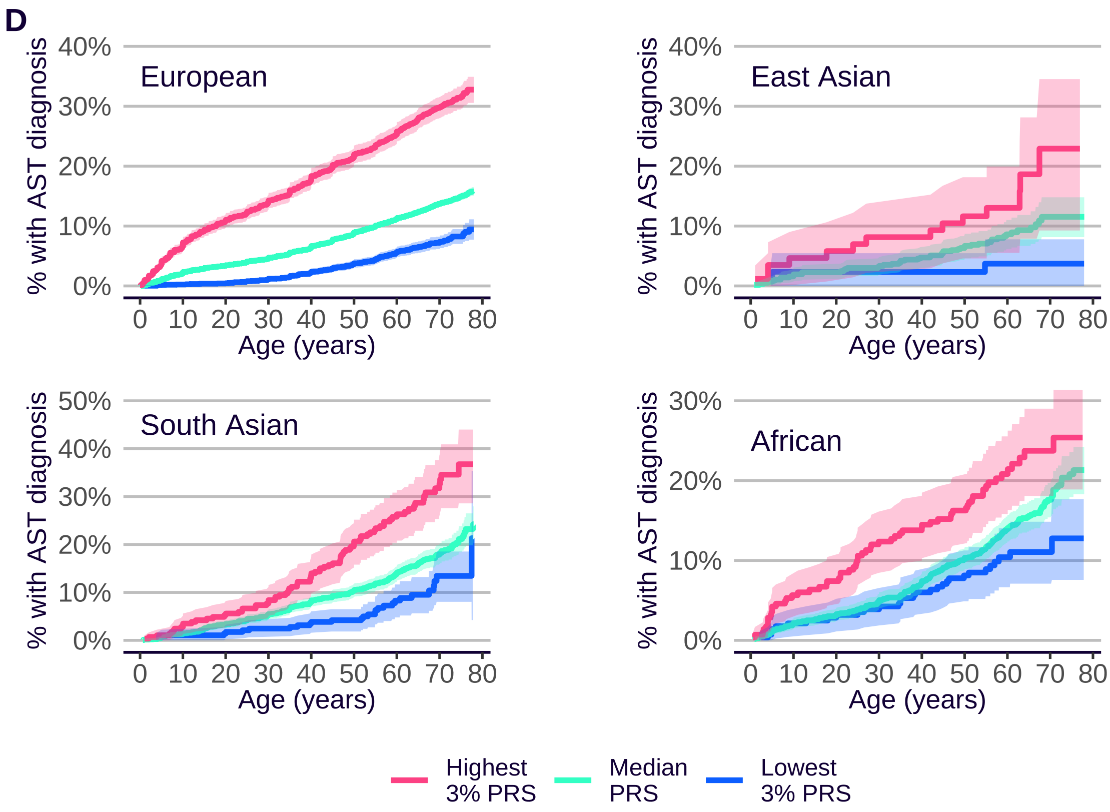

D
European
East Asian
South Asian
African
% with AST diagnosis
Age (years)
0%
10%
20%
30%
40%
50%
0
10
20
30
40
50
60
70
80
Highest 3% PRS
Median PRS
Lowest 3% PRS

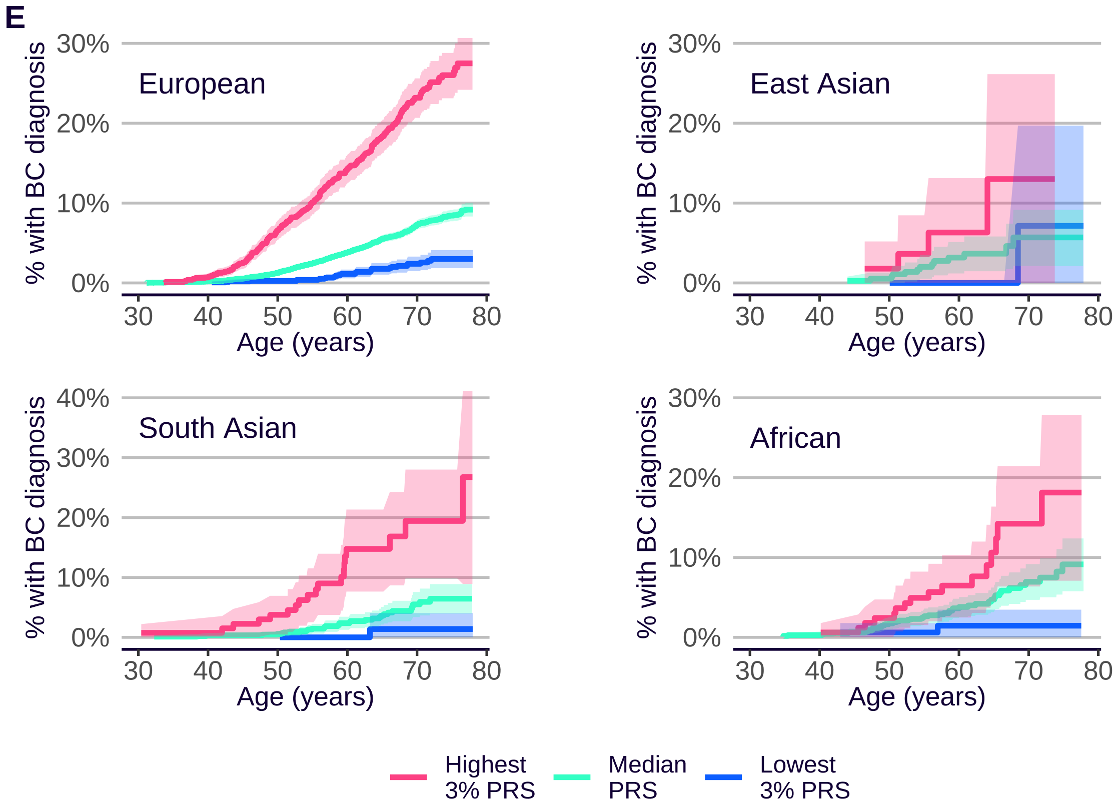
E
European
East Asian
South Asian
African
% with BC diagnosis
Age (years)
0%
10%
20%
30%
40%
30
40
50
60
70
80
Highest 3% PRS
Median PRS
Lowest 3% PRS

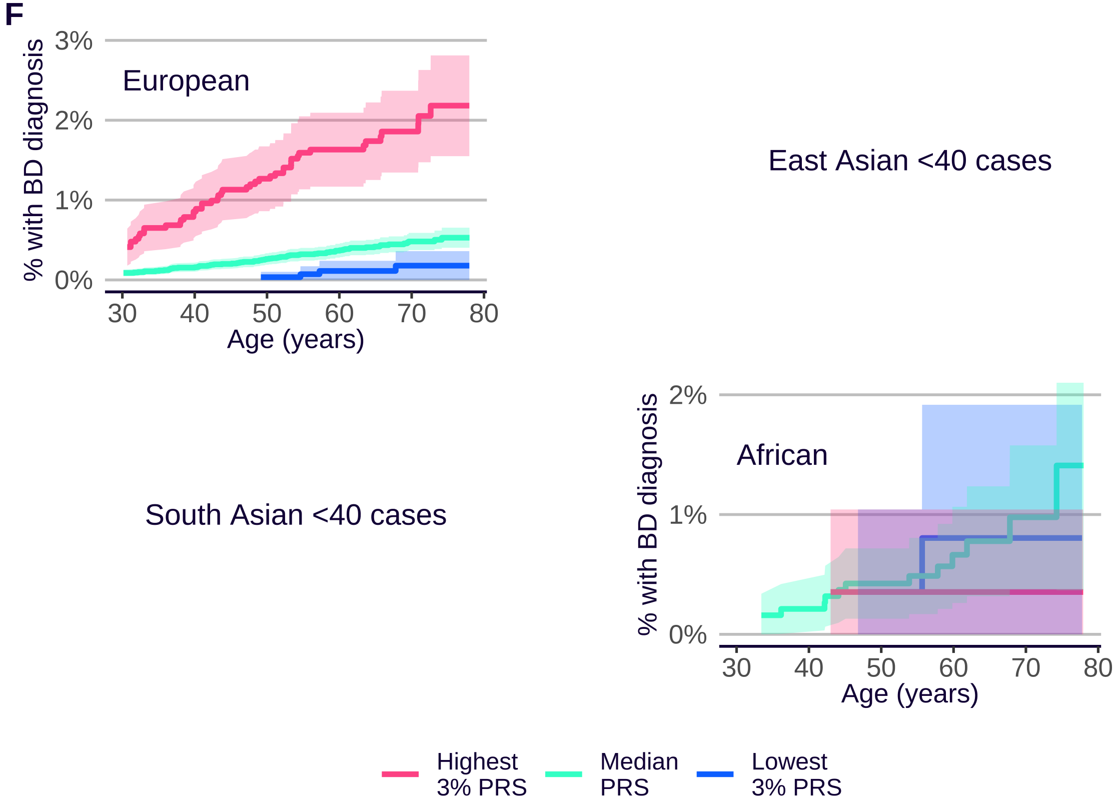
F
European
3%
2%
1%
0%
% with BD diagnosis
30
40
50
60
70
80
Age (years)
East Asian <40 cases
South Asian <40 cases
African
2%
1%
0%
% with BD diagnosis
30
40
50
60
70
80
Age (years)
Highest 3% PRS
Median PRS
Lowest 3% PRS

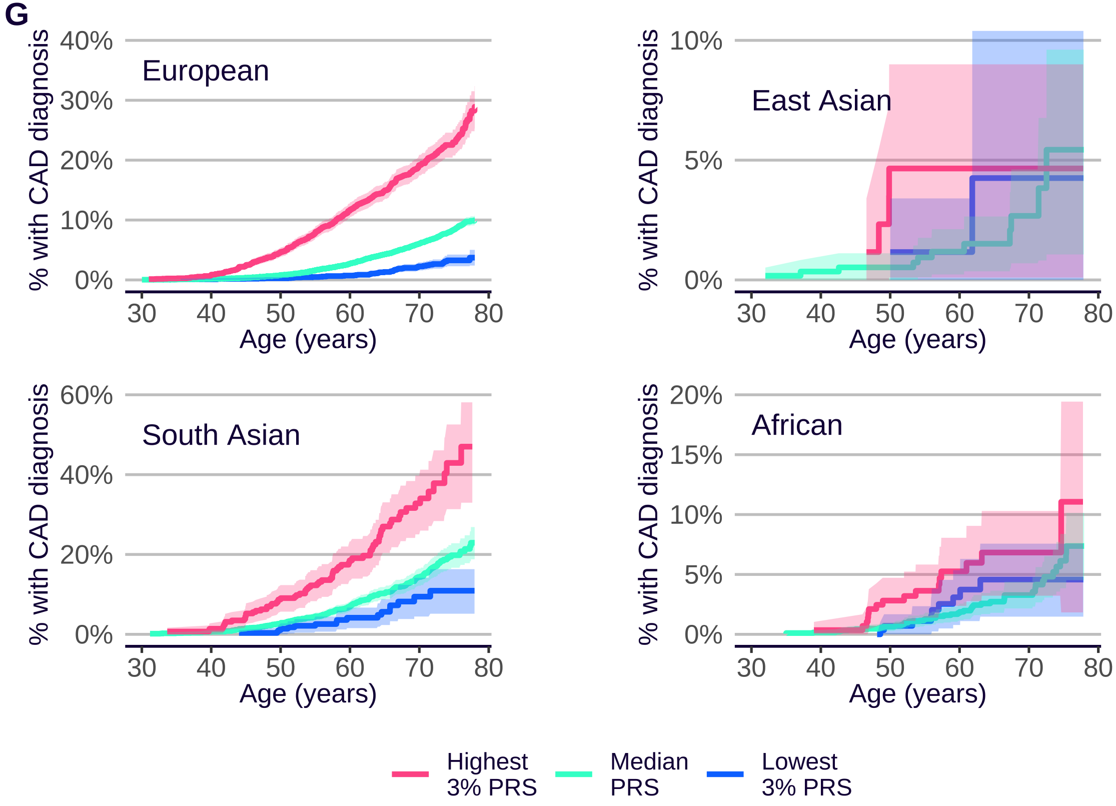
G
European
East Asian
South Asian
African
% with CAD diagnosis
Age (years)
0%
10%
20%
30%
40%
5%
15%
60%
30
40
50
60
70
80
Highest 3% PRS
Median PRS
Lowest 3% PRS

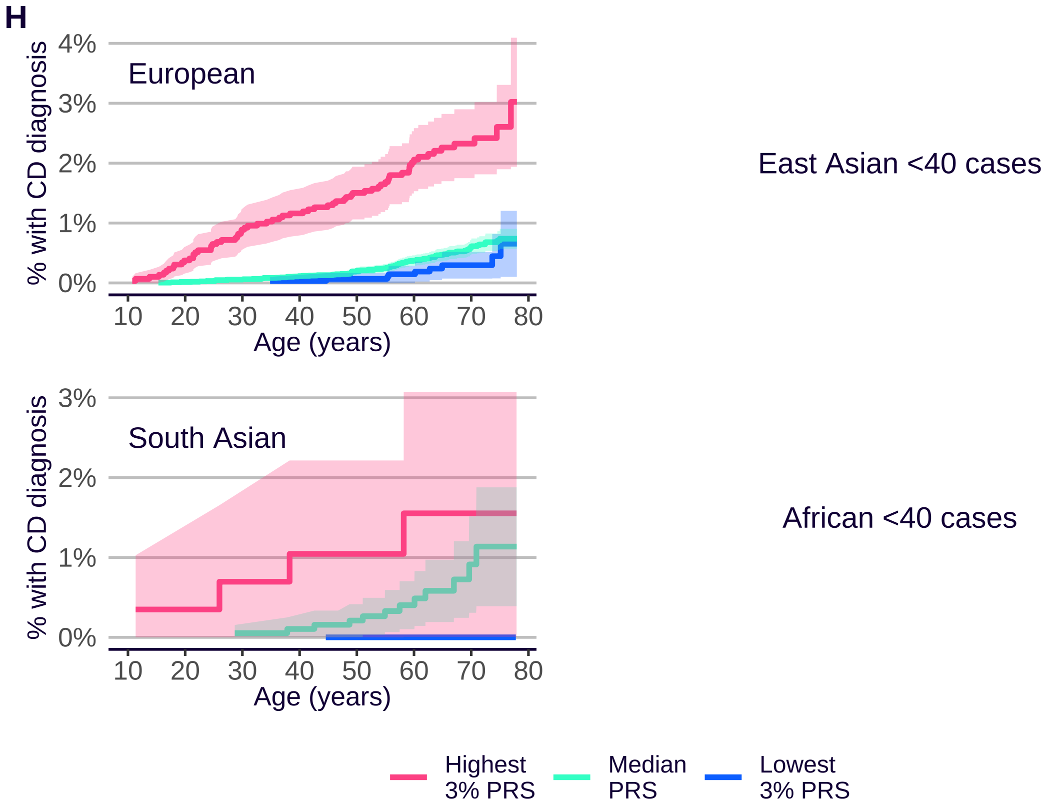
H
European
South Asian
East Asian <40 cases
African <40 cases
% with CD diagnosis
0%
1%
2%
3%
4%
Age (years)
10
20
30
40
50
60
70
80
Highest 3% PRS
Median PRS
Lowest 3% PRS

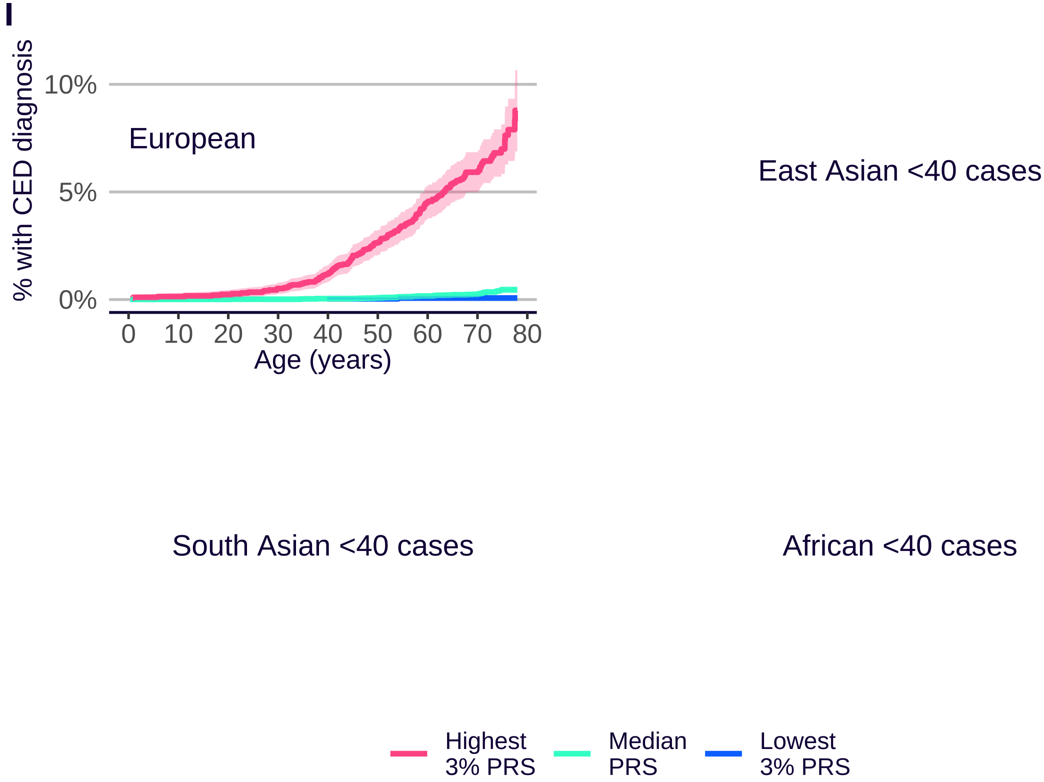
I
% with CED diagnosis
10%
5%
0%
European
0
10
20
30
40
50
60
70
80
Age (years)
East Asian <40 cases
South Asian <40 cases
African <40 cases
Highest 3% PRS
Median PRS
Lowest 3% PRS

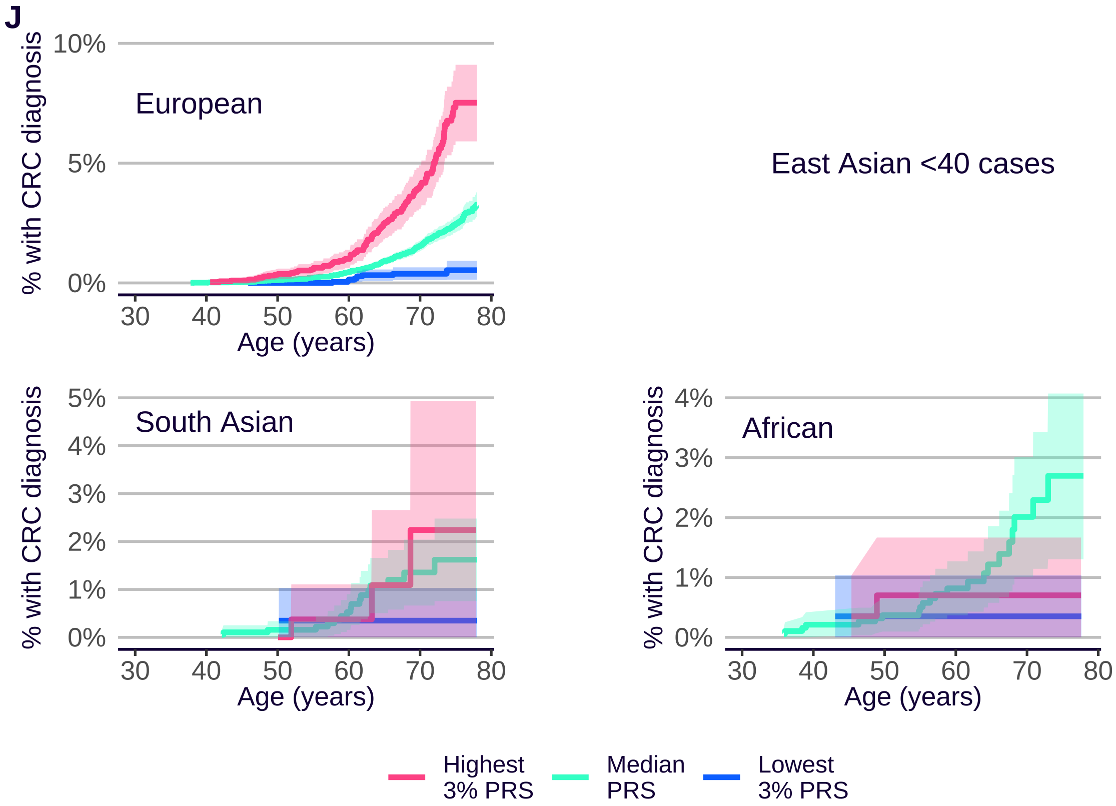
J
European
East Asian <40 cases
South Asian
African
% with CRC diagnosis
Age (years)
10%
5%
0%
4%
3%
2%
1%
30
40
50
60
70
80
Highest 3% PRS
Median PRS
Lowest 3% PRS

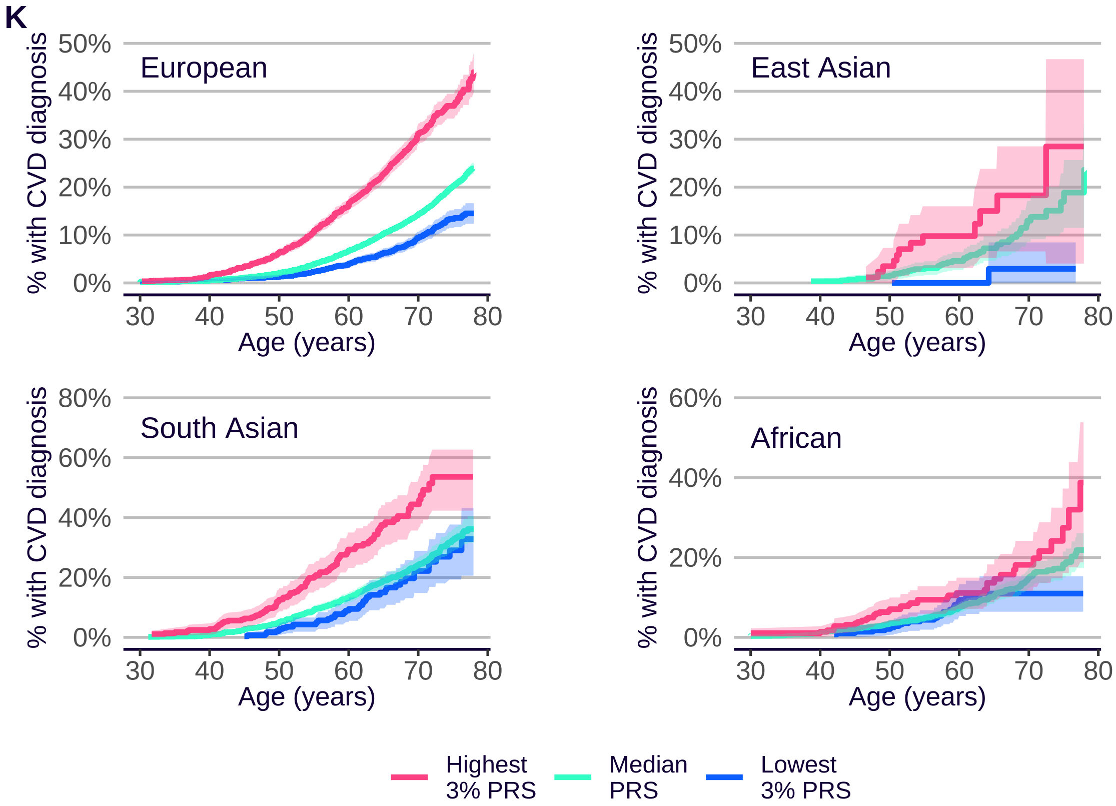

K
European
East Asian
South Asian
African
% with CVD diagnosis
0%
10%
20%
30%
40%
50%
60%
80%
30
40
50
60
70
80
Age (years)
Highest 3% PRS
Median PRS
Lowest 3% PRS

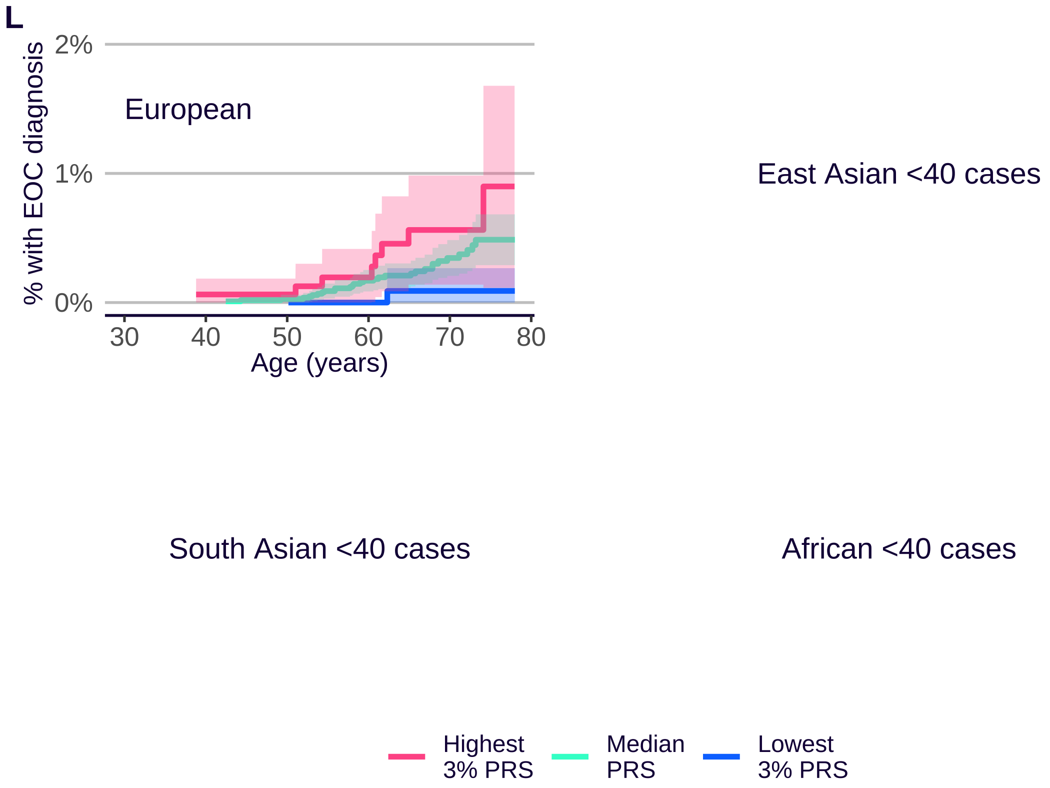
L
% with EOC diagnosis
2%
1%
0%
European
30
40
50
60
70
80
Age (years)
East Asian <40 cases
South Asian <40 cases
African <40 cases
Highest
3% PRS
Median
PRS
Lowest
3% PRS

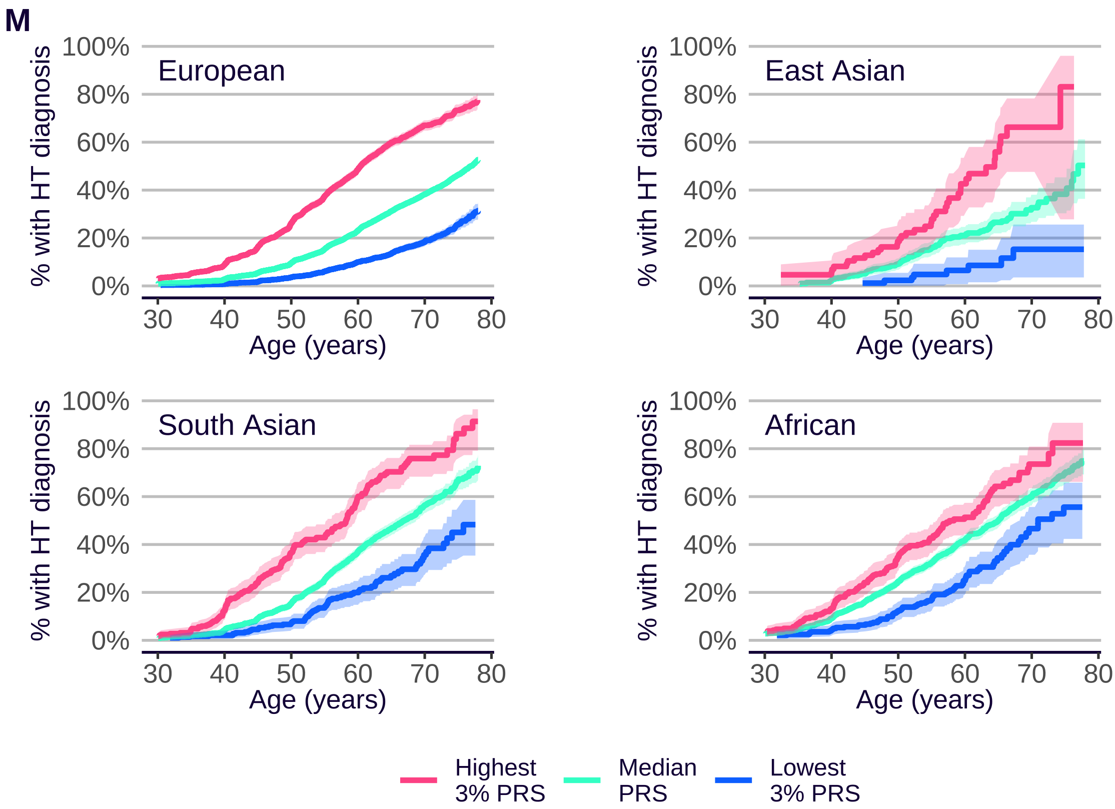

M
European
East Asian
South Asian
African
% with HT diagnosis
Age (years)
100%
80%
60%
40%
20%
0%
30
40
50
60
70
80
Highest
3% PRS
Median
PRS
Lowest
3% PRS

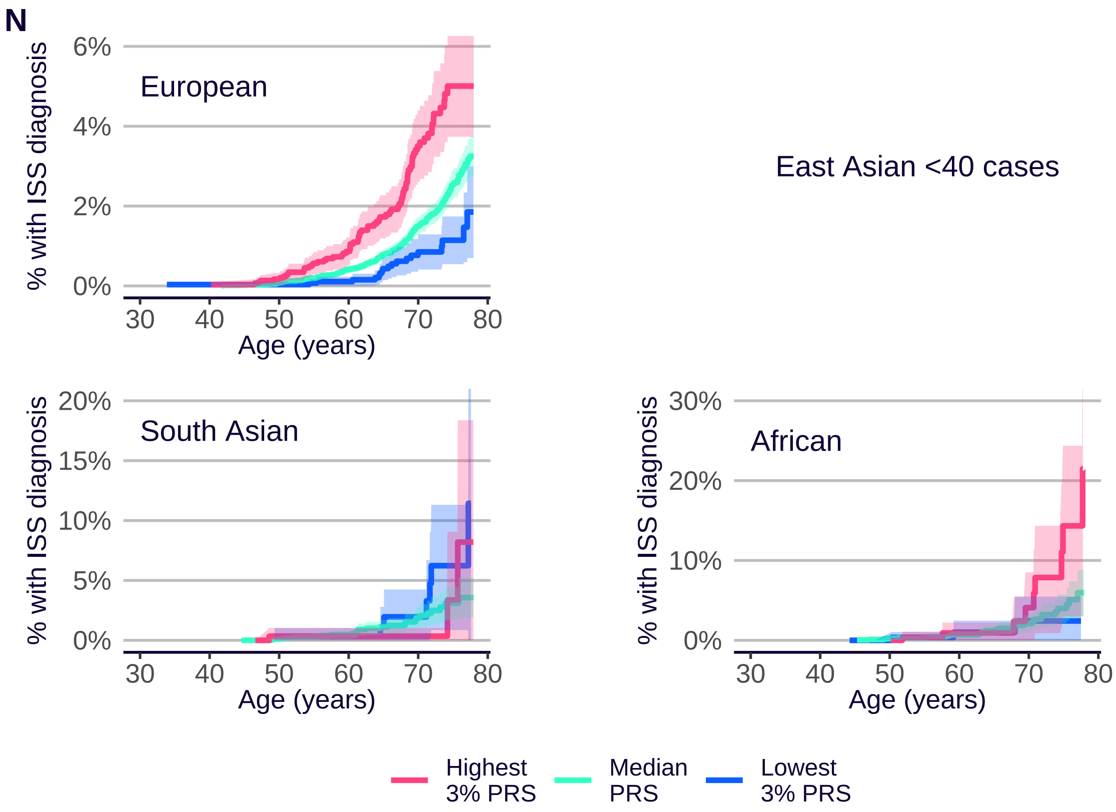
N
European
% with ISS diagnosis
6%
4%
2%
0%
30 40 50 60 70 80
Age (years)
East Asian <40 cases
South Asian
20%
15%
10%
5%
0%
African
30%
20%
10%
0%
Highest 3% PRS
Median PRS
Lowest 3% PRS

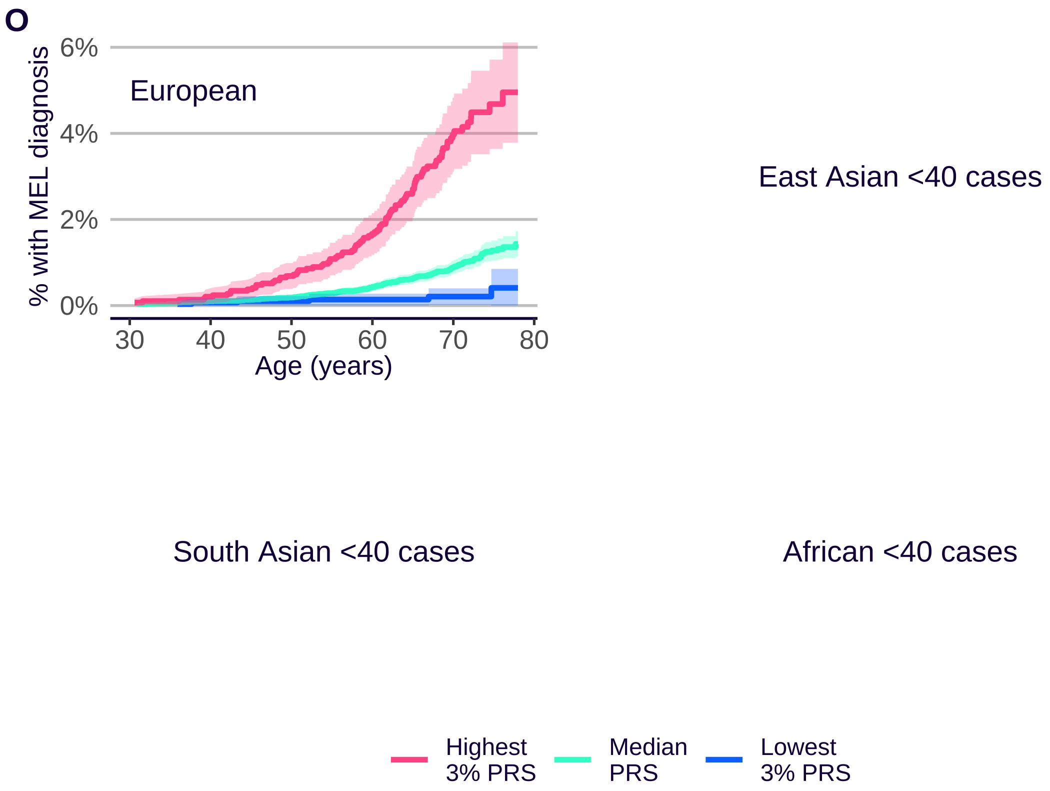
O
European
% with MEL diagnosis
6%
4%
2%
0%
30
40
50
60
70
80
Age (years)
East Asian <40 cases
South Asian <40 cases
African <40 cases
Highest 3% PRS
Median PRS
Lowest 3% PRS

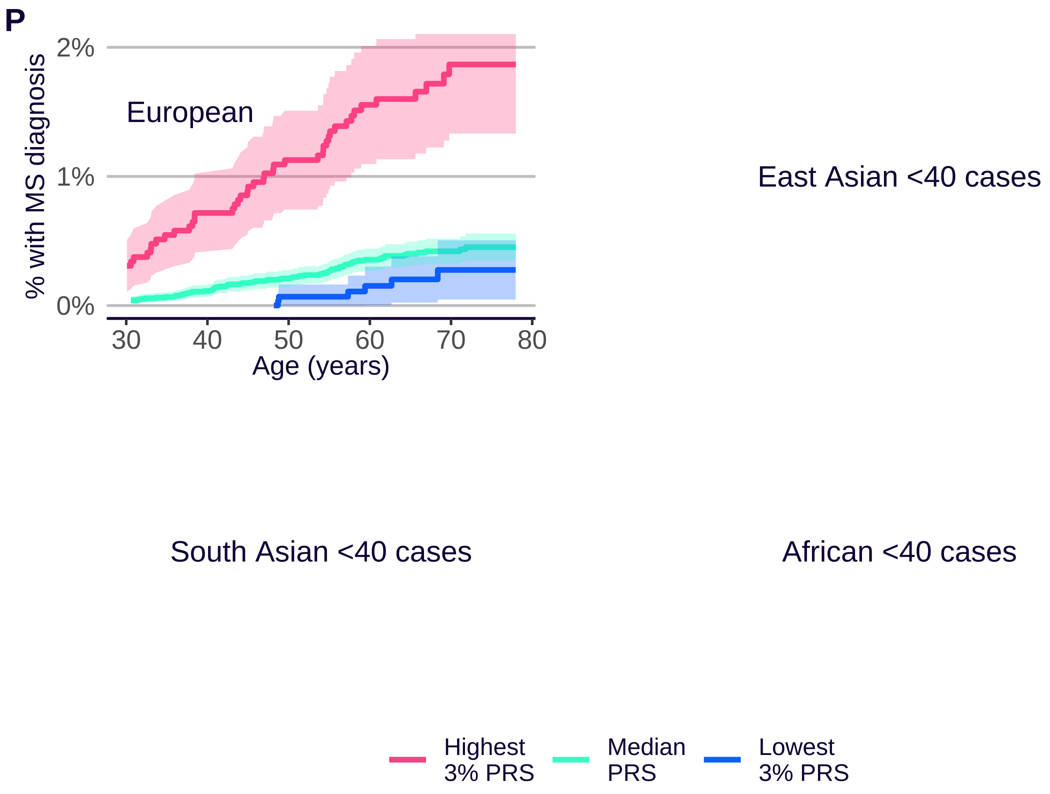
P
2%
1%
0%
% with MS diagnosis
European
30
40
50
60
70
80
Age (years)
East Asian <40 cases
South Asian <40 cases
African <40 cases
Highest 3% PRS
Median PRS
Lowest 3% PRS

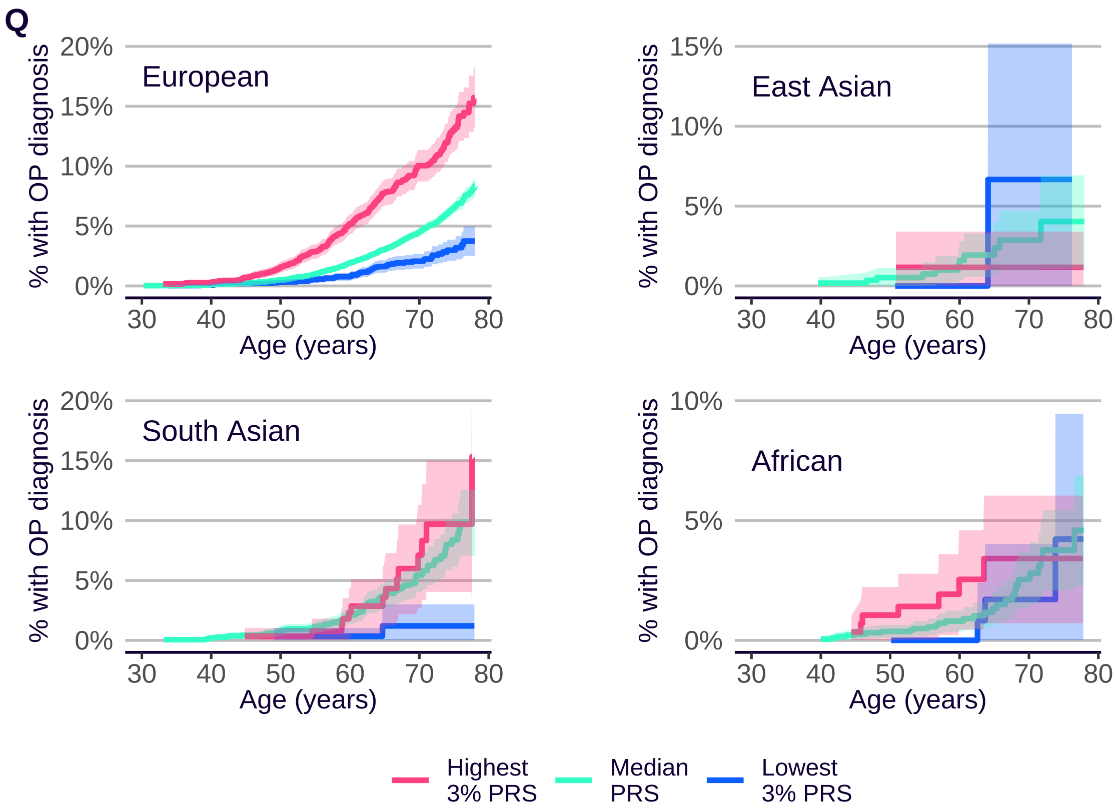

Q
European
East Asian
South Asian
African
% with OP diagnosis
Age (years)
0%
5%
10%
15%
20%
30
40
50
60
70
80
Highest 3% PRS
Median PRS
Lowest 3% PRS

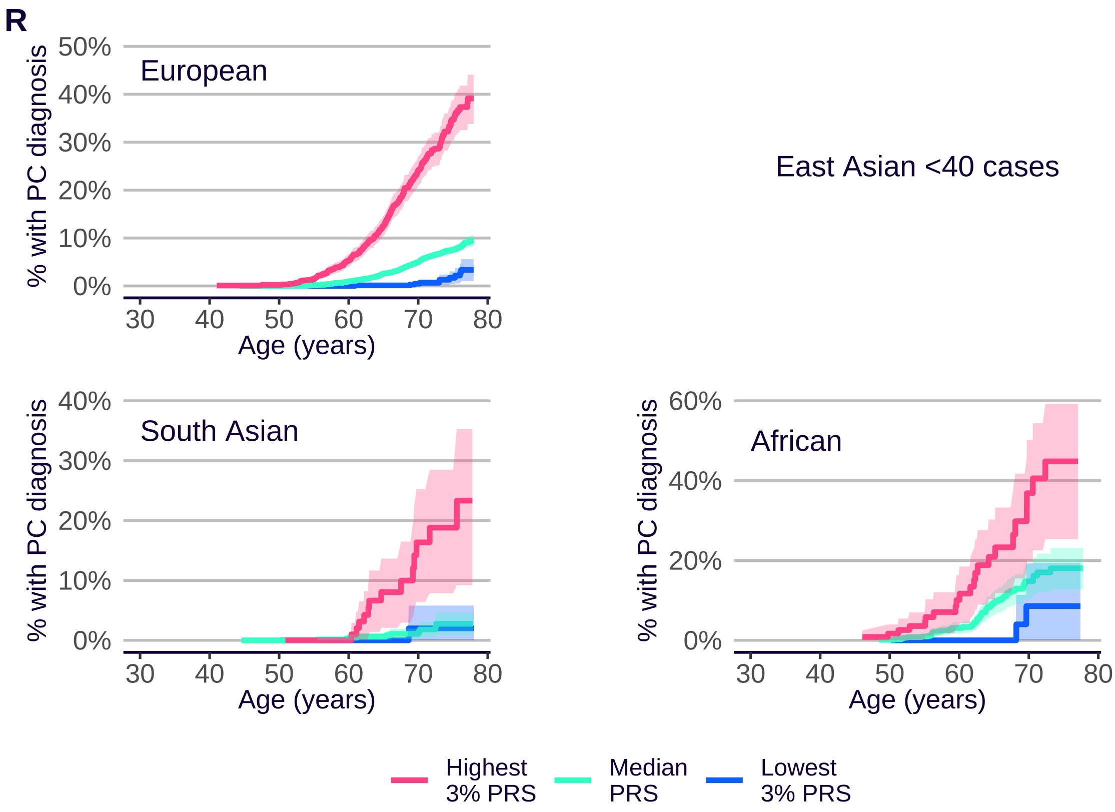
R
European
East Asian <40 cases
South Asian
African
% with PC diagnosis
0%
10%
20%
30%
40%
50%
60%
30
40
50
60
70
80
Age (years)
Highest 3% PRS
Median PRS
Lowest 3% PRS

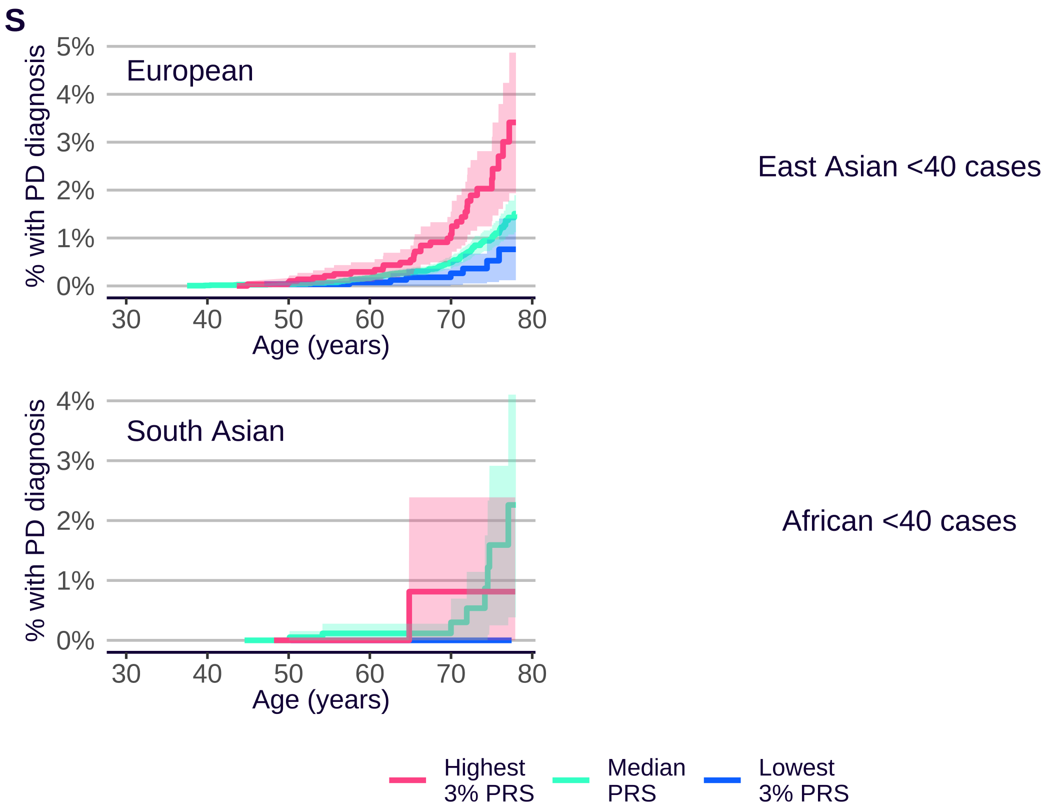

S
European
5%
4%
3%
2%
1%
0%
% with PD diagnosis
30
40
50
60
70
80
Age (years)
East Asian <40 cases
South Asian
4%
3%
2%
1%
0%
% with PD diagnosis
30
40
50
60
70
80
Age (years)
African <40 cases
Highest 3% PRS
Median PRS
Lowest 3% PRS

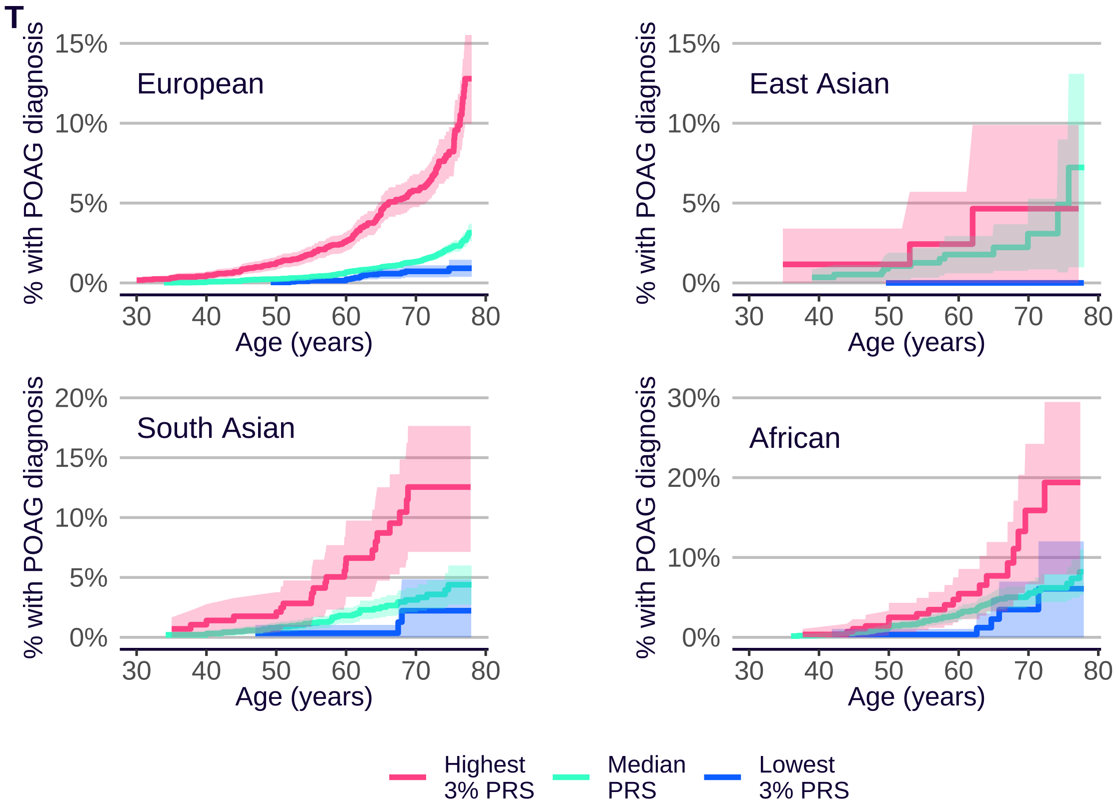
T
European
East Asian
South Asian
African
% with POAG diagnosis
Age (years)
0%
5%
10%
15%
20%
30%
30
40
50
60
70
80
Highest 3% PRS
Median PRS
Lowest 3% PRS

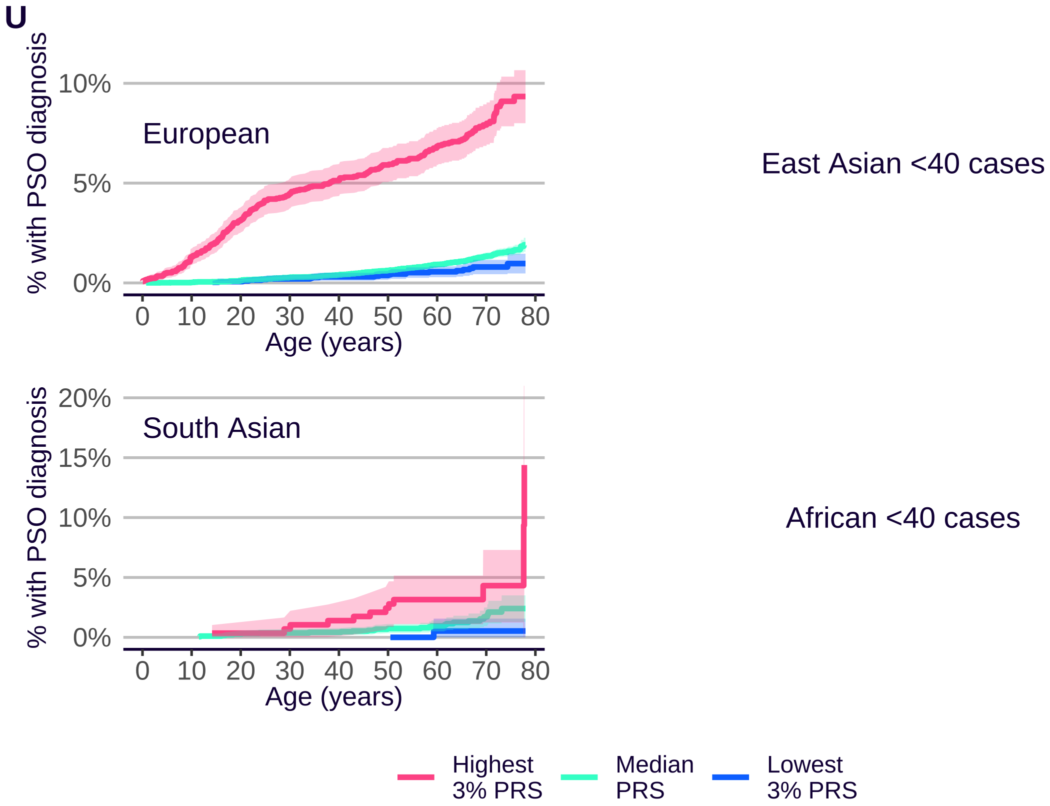
U
European
% with PSO diagnosis
10%
5%
0%
0 10 20 30 40 50 60 70 80
Age (years)
East Asian <40 cases
South Asian
% with PSO diagnosis
20%
15%
10%
5%
0%
0 10 20 30 40 50 60 70 80
Age (years)
African <40 cases
Highest 3% PRS
Median PRS
Lowest 3% PRS

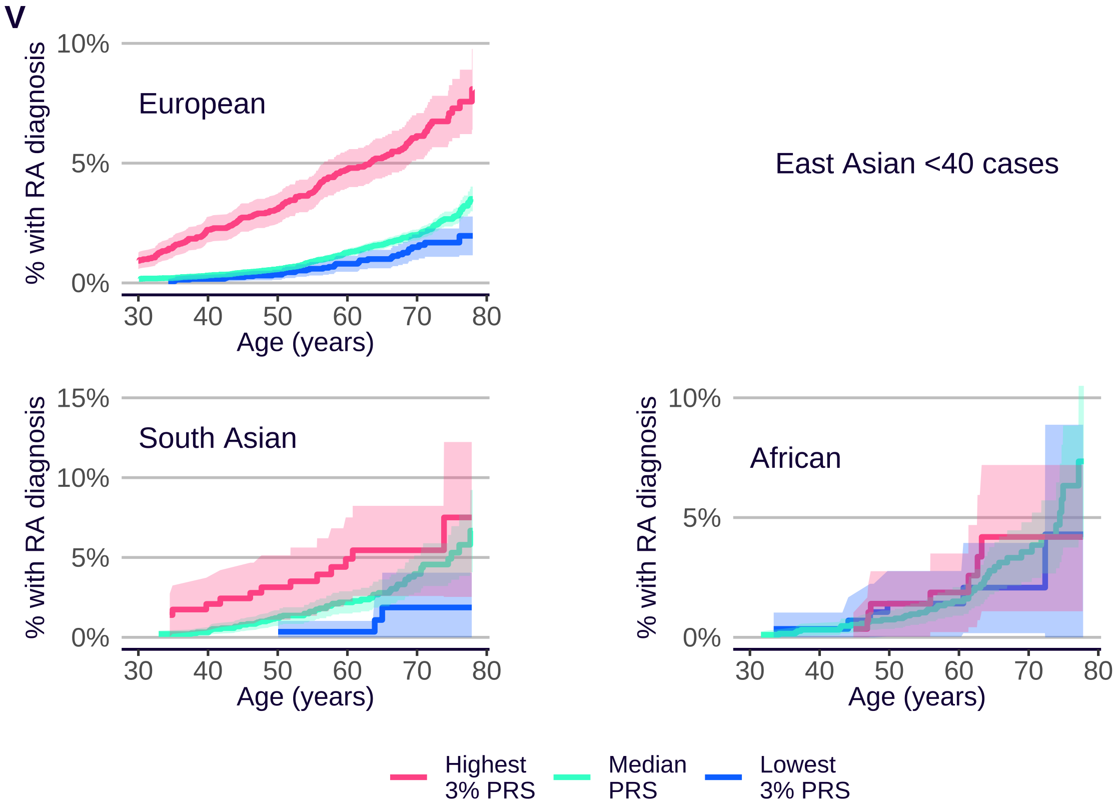
V
European
East Asian <40 cases
South Asian
African
% with RA diagnosis
10%
5%
0%
15%
30
40
50
60
70
80
Age (years)
Highest 3% PRS
Median PRS
Lowest 3% PRS

W

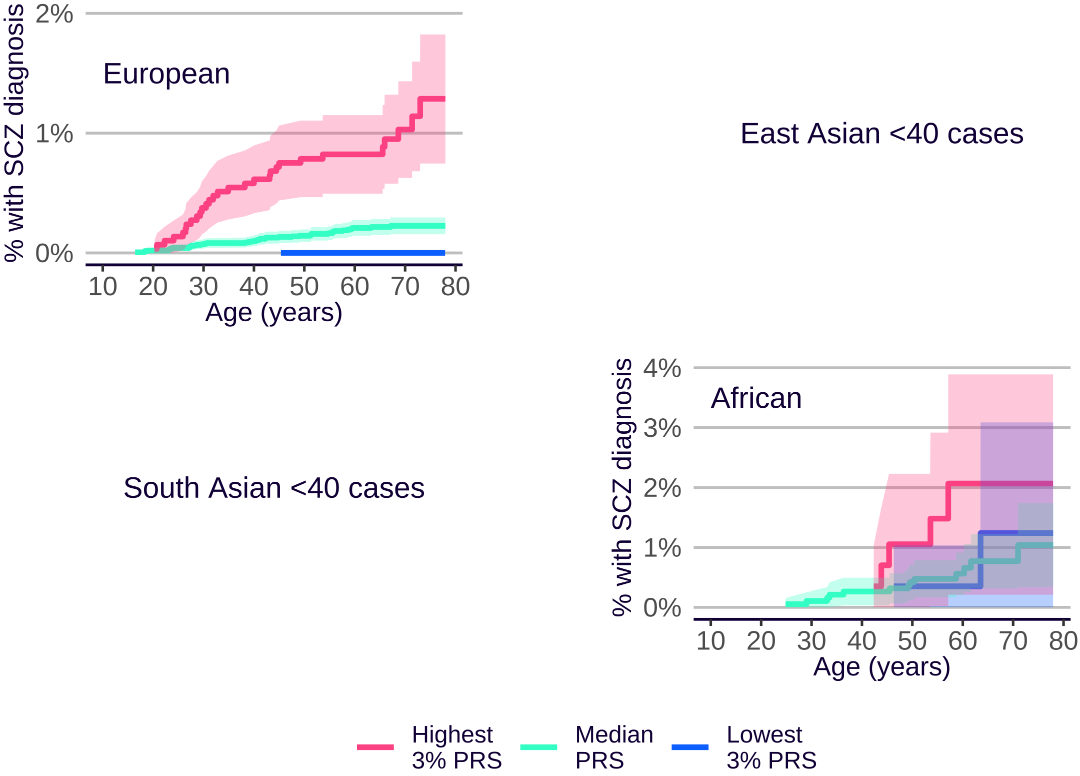

% with SCZ diagnosis
2%
1%
0%
European
10 20 30 40 50 60 70 80
Age (years)
East Asian <40 cases
South Asian <40 cases
% with SCZ diagnosis
4%
3%
2%
1%
0%
African
10 20 30 40 50 60 70 80
Age (years)
Highest 3% PRS
Median PRS
Lowest 3% PRS

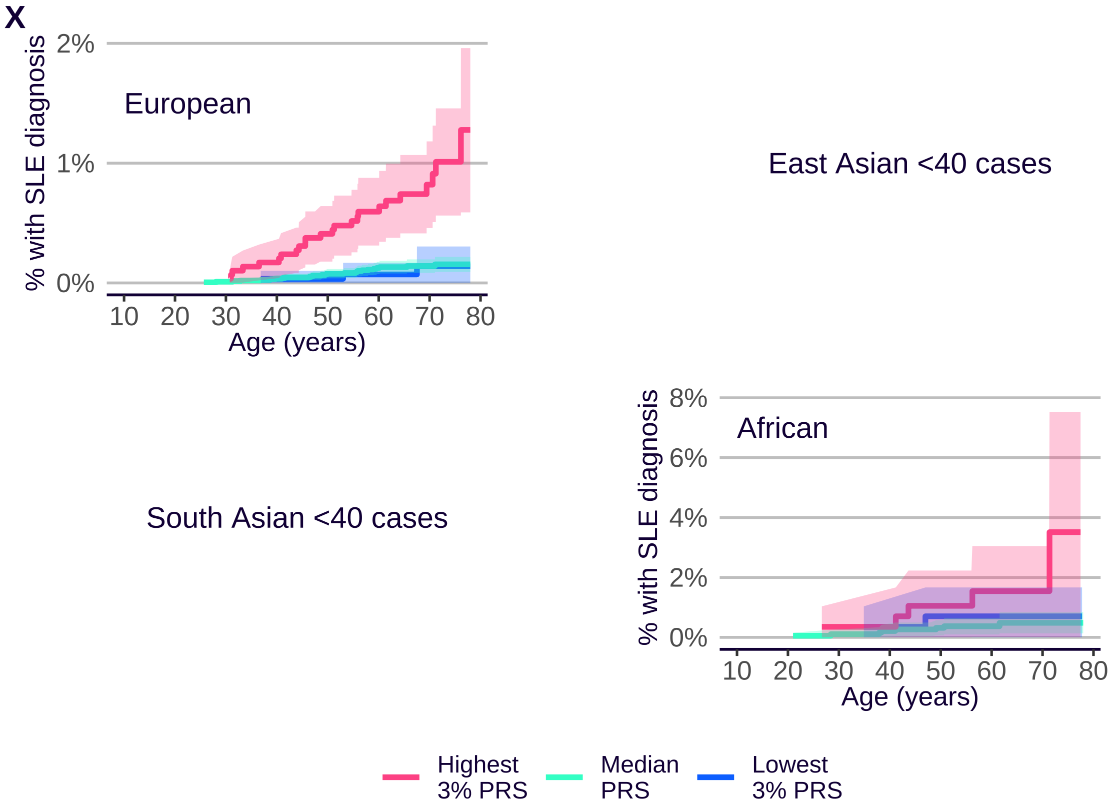

X
European
% with SLE diagnosis
2%
1%
0%
10 20 30 40 50 60 70 80
Age (years)
East Asian <40 cases
South Asian <40 cases
African
8%
6%
4%
2%
0%
Highest 3% PRS
Median PRS
Lowest 3% PRS

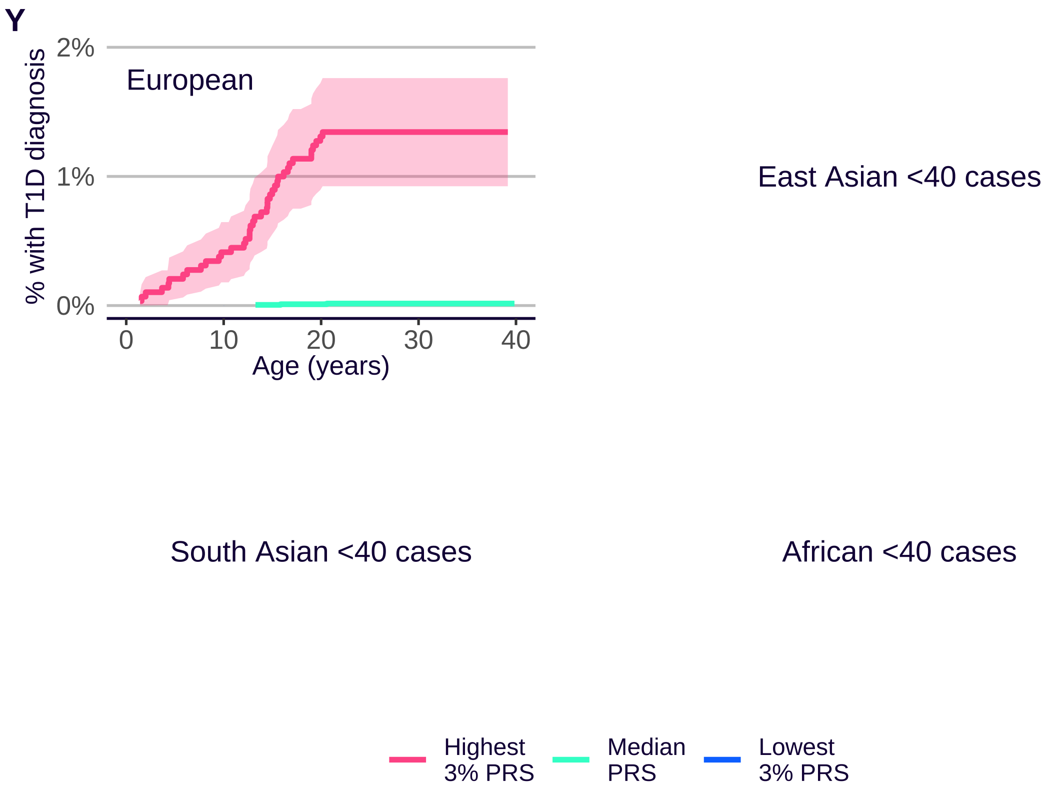

Y
% with T1D diagnosis
2%
1%
0%
European
0
10
20
30
40
Age (years)
East Asian <40 cases
South Asian <40 cases
African <40 cases
Highest 3% PRS
Median PRS
Lowest 3% PRS

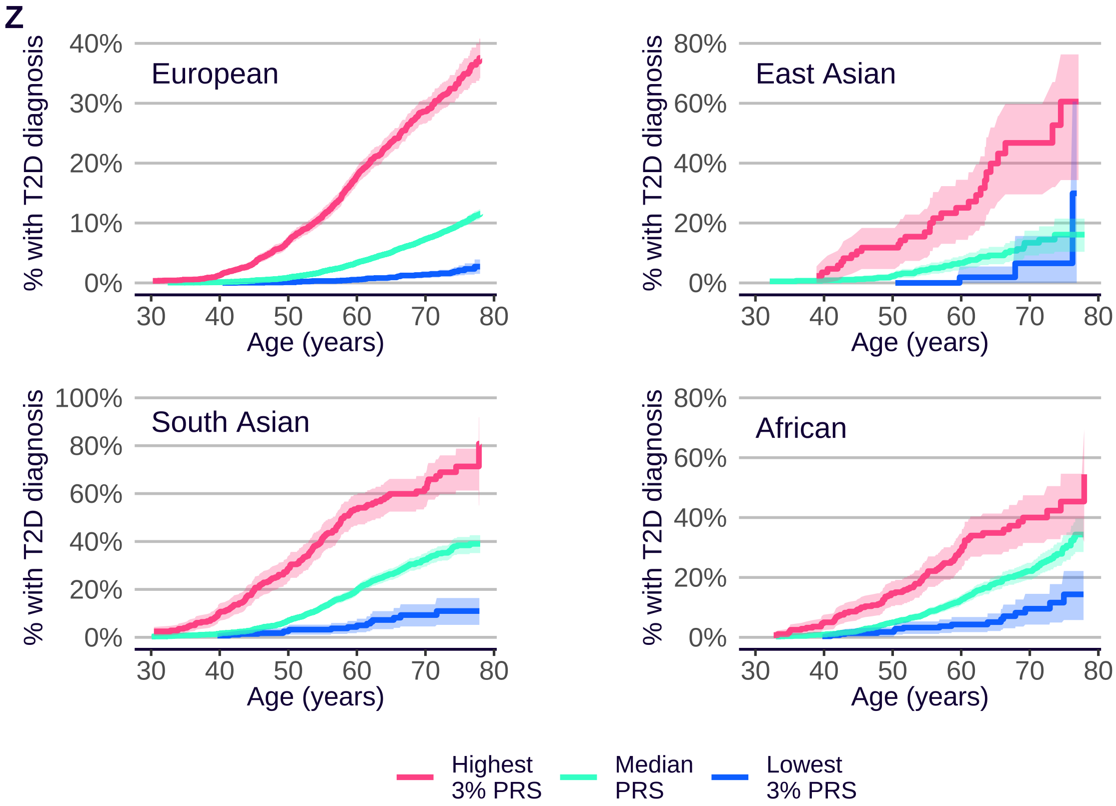

Z
European
East Asian
South Asian
African
% with T2D diagnosis
Age (years)
0%
10%
20%
30%
40%
60%
80%
100%
30
40
50
60
70
80
Highest 3% PRS
Median PRS
Lowest 3% PRS

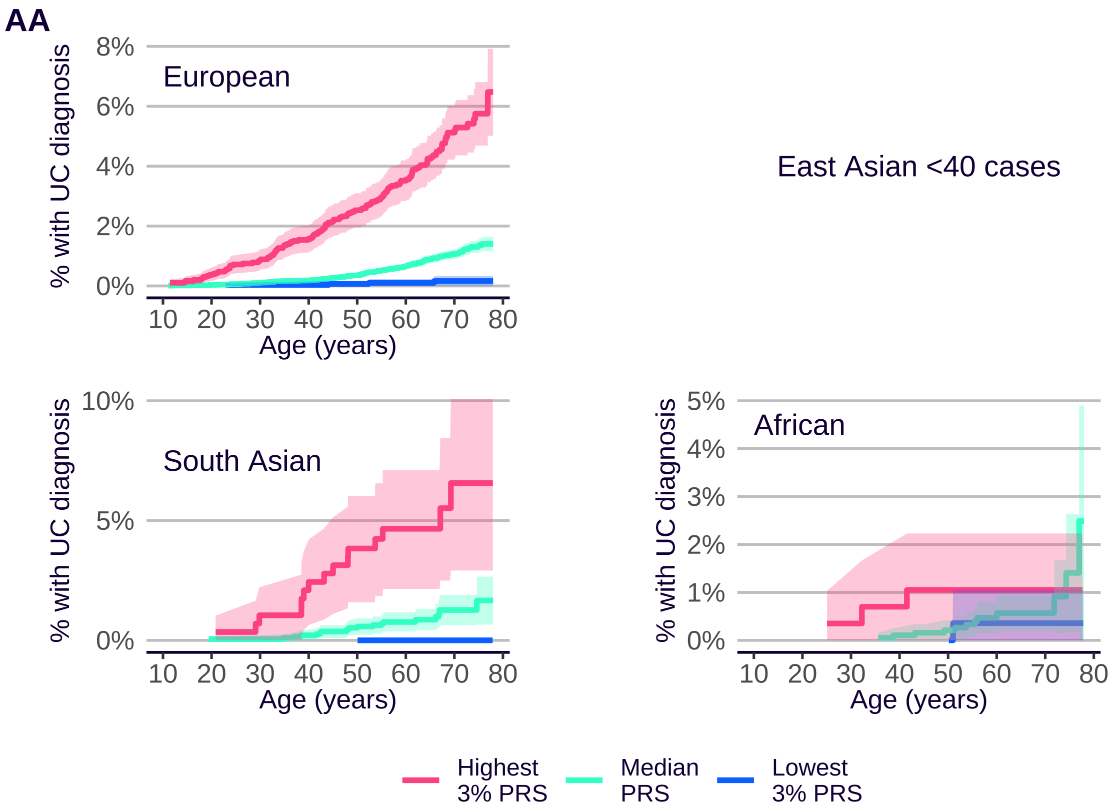

AA
European
East Asian <40 cases
South Asian
African
% with UC diagnosis
Age (years)
0%
2%
4%
6%
8%
5%
10%
1%
3%
10
20
30
40
50
60
70
80
Highest 3% PRS
Median PRS
Lowest 3% PRS

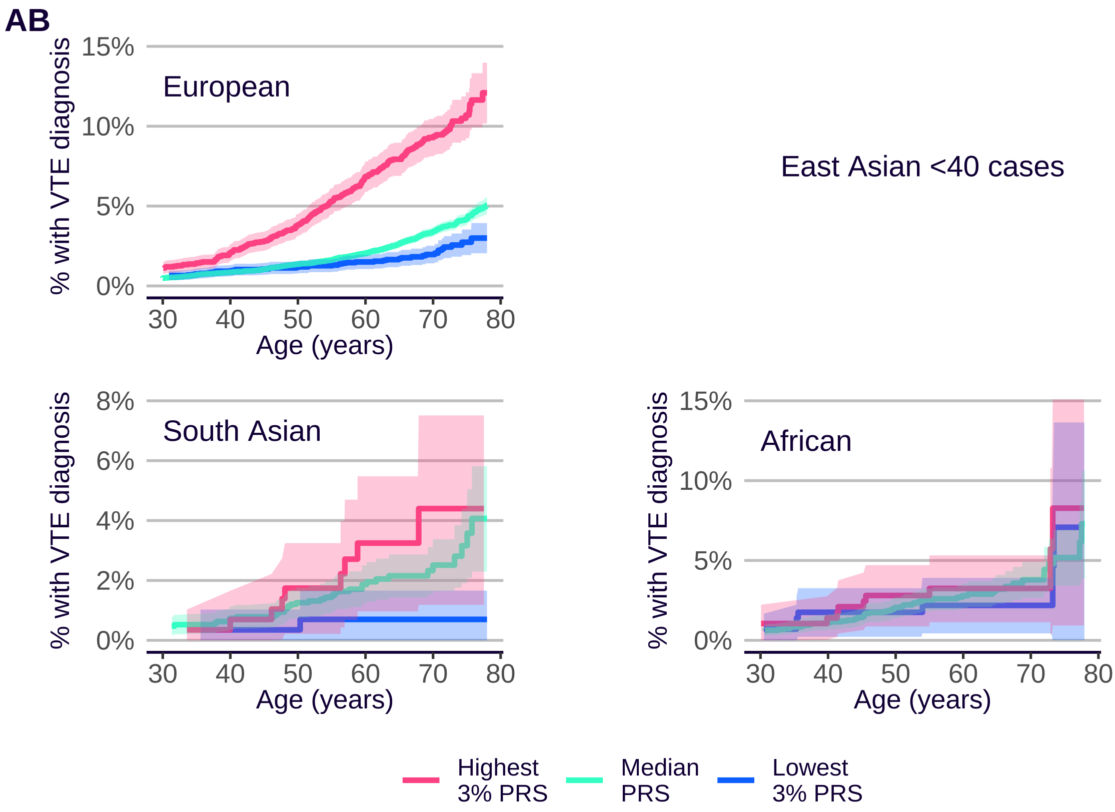
AB
European
East Asian <40 cases
South Asian
African
% with VTE diagnosis
Age (years)
15%
10%
5%
0%
8%
6%
4%
2%
30
40
50
60
70
80
Highest 3% PRS
Median PRS
Lowest 3% PRS
