# Supplementary material for: A systematic evaluation of the performance and properties of the UK Biobank Polygenic Risk Score (PRS) Release
Source: PLoS One. 2024 Sep 18;19(9):e0307270. doi: 10.1371/journal.pone.0307270 (PMC11410272; doi:10.1371/journal.pone.0307270)
Supplement: S11 Fig — Cumulative incidence plots are shown for each disease and each ancestry group in the UKB Testing Subgroup, provided more than 40 cases are available (the number of cases is printed otherwise), with separate curves for the highest 3% (red), lowest 3% (blue), and median 40–60% (green) of the PRS distribution. A. Alzheimer’s disease (AD). B. Atrial fibrillation (AF). C. Age-related macular degeneration (AMD). D. Asthma (AST). E. Breast cancer (BC), F. Bipolar disorder (BD), G. Coronary artery disease (CAD). H. Crohn’s disease (CD). I. Coeliac disease (CED). J. Bowel cancer (CRC). K. Cardiovascular disease (CVD), L. Epithelial ovarian cancer (EOC). M. Hypertension (HT). N. Ischaemic stroke (ISS). O. Melanoma (MEL). P. Multiple sclerosis (MS). Q. Osteoporosis (OP). R. Prostate cancer (PC). S. Parkinson’s disease (PD). T. Primary open angle glaucoma (POAG). U. Psoriasis (PSO). V. Rheumatoid arthritis (RA). W. Schizophrenia (SCZ). X. Systemic lupus erythematosus (SLE). Y. Type 1 diabetes (T1D). Z. Type 2 diabetes (T2D). AA. Ulcerative colitis (UC). AB. Venous thromboembolic disease (VTE). EUR = European ancestry group. EAS = East Asian ancestry group. SAS = South Asian ancestry group. AFR = Sub-Saharan African ancestry group. Shaded areas indicate 95% CI. (PDF) [file pone.0307270.s023.pdf]

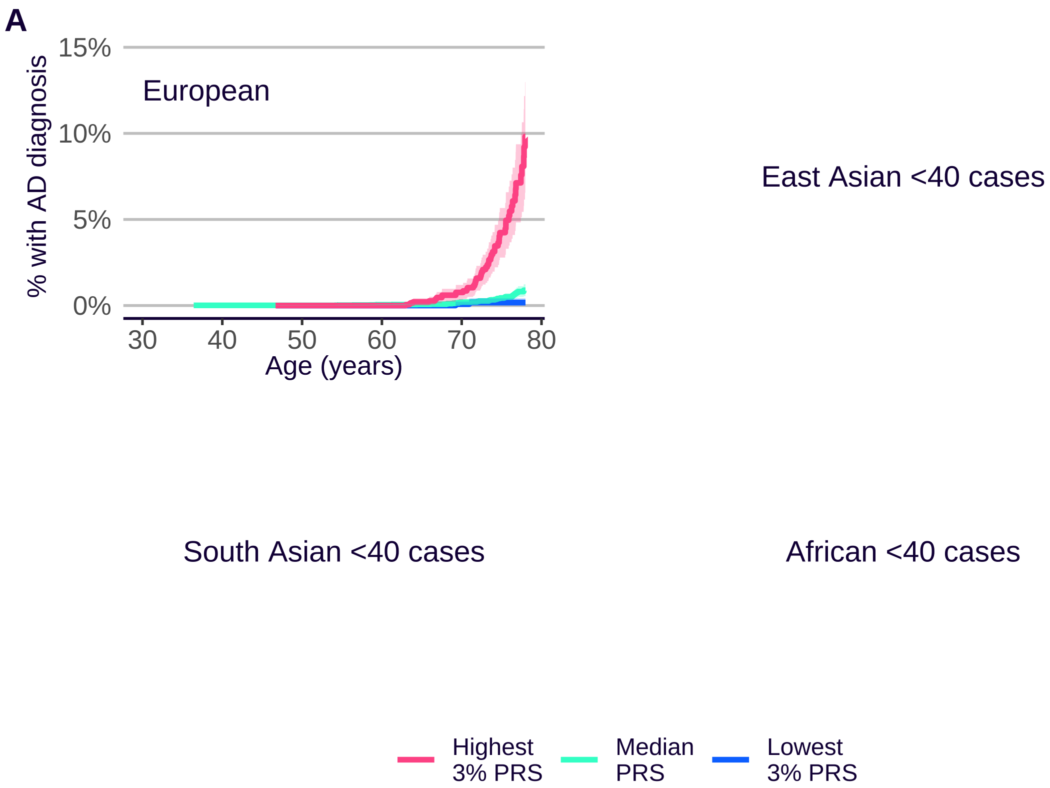
A
European
% with AD diagnosis
15%
10%
5%
0%
30
40
50
60
70
80
Age (years)
East Asian <40 cases
South Asian <40 cases
African <40 cases
Highest 3% PRS
Median PRS
Lowest 3% PRS

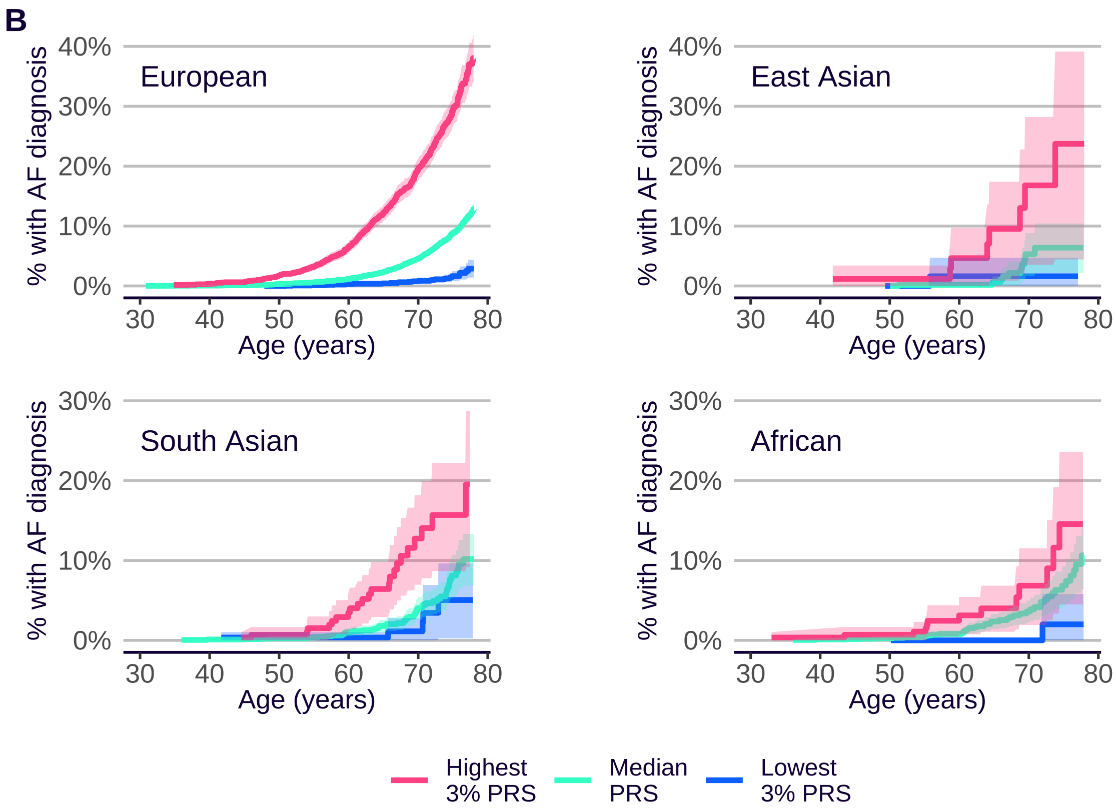
B
European
East Asian
South Asian
African
% with AF diagnosis
Age (years)
40%
30%
20%
10%
0%
30
40
50
60
70
80
Highest
3% PRS
Median
PRS
Lowest
3% PRS

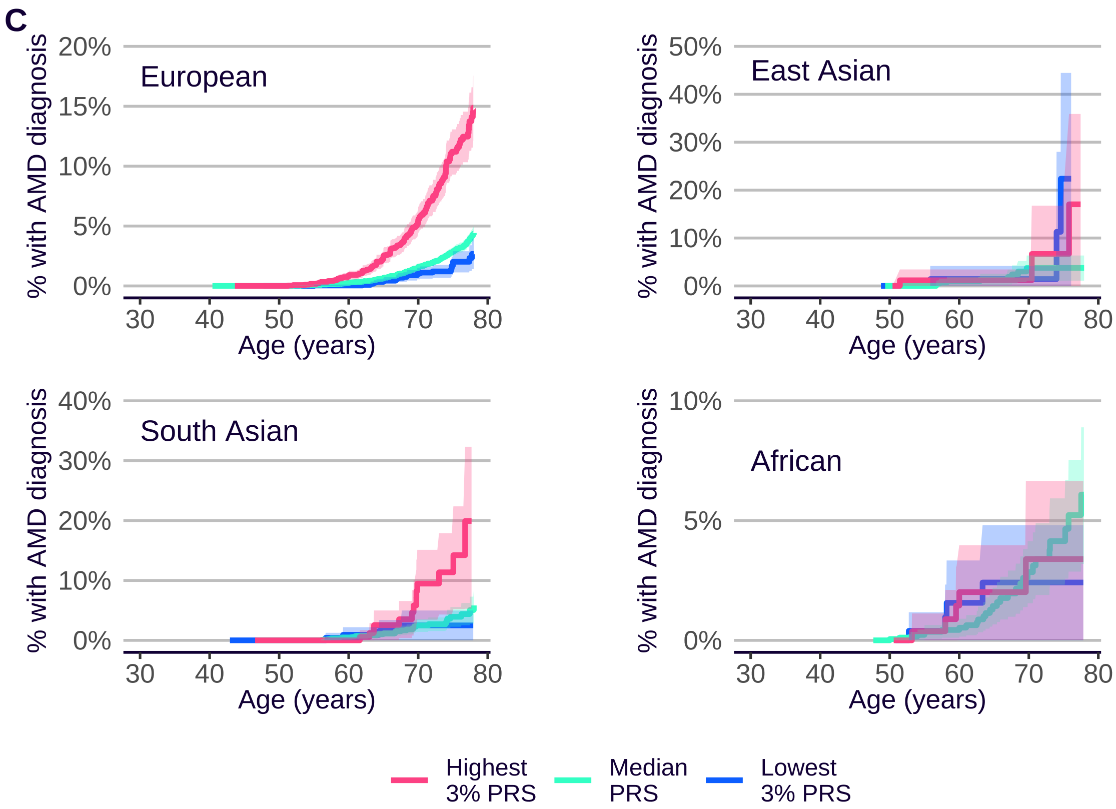
C
European
East Asian
South Asian
African
% with AMD diagnosis
Age (years)
0%
5%
10%
15%
20%
30%
40%
50%
30
40
50
60
70
80
Highest
3% PRS
Median
PRS
Lowest
3% PRS

D

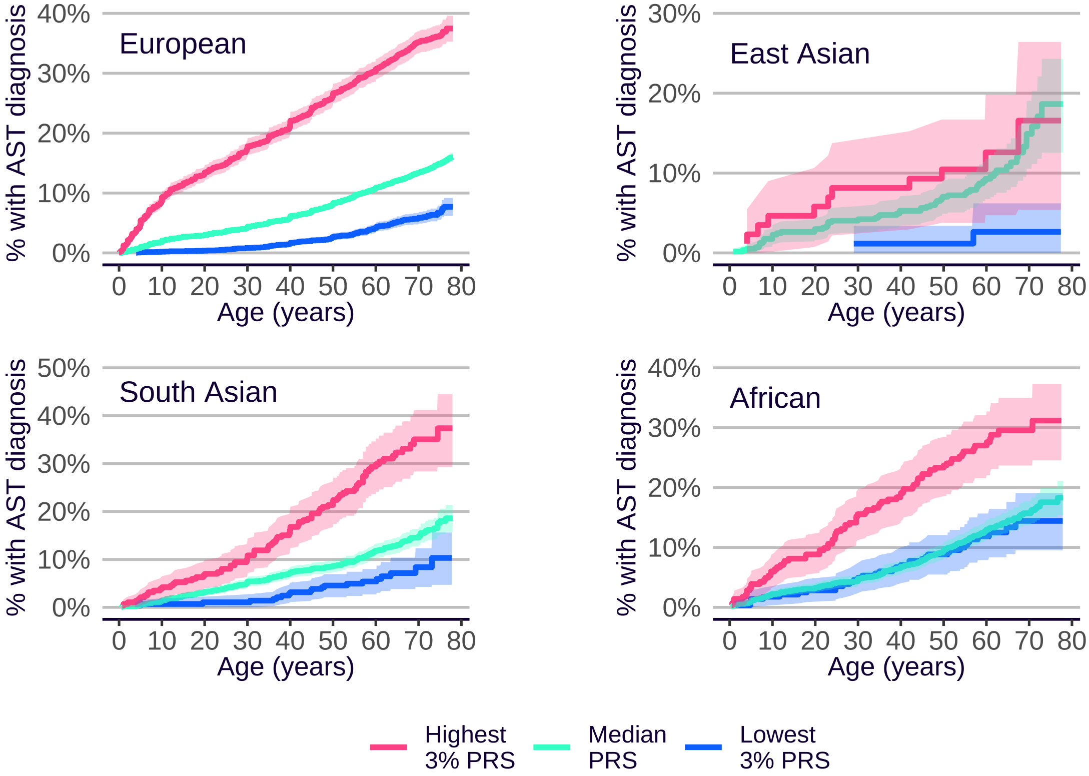
European
East Asian
South Asian
African
% with AST diagnosis
Age (years)
0%
10%
20%
30%
40%
50%
0
10
20
30
40
50
60
70
80
Highest 3% PRS
Median PRS
Lowest 3% PRS

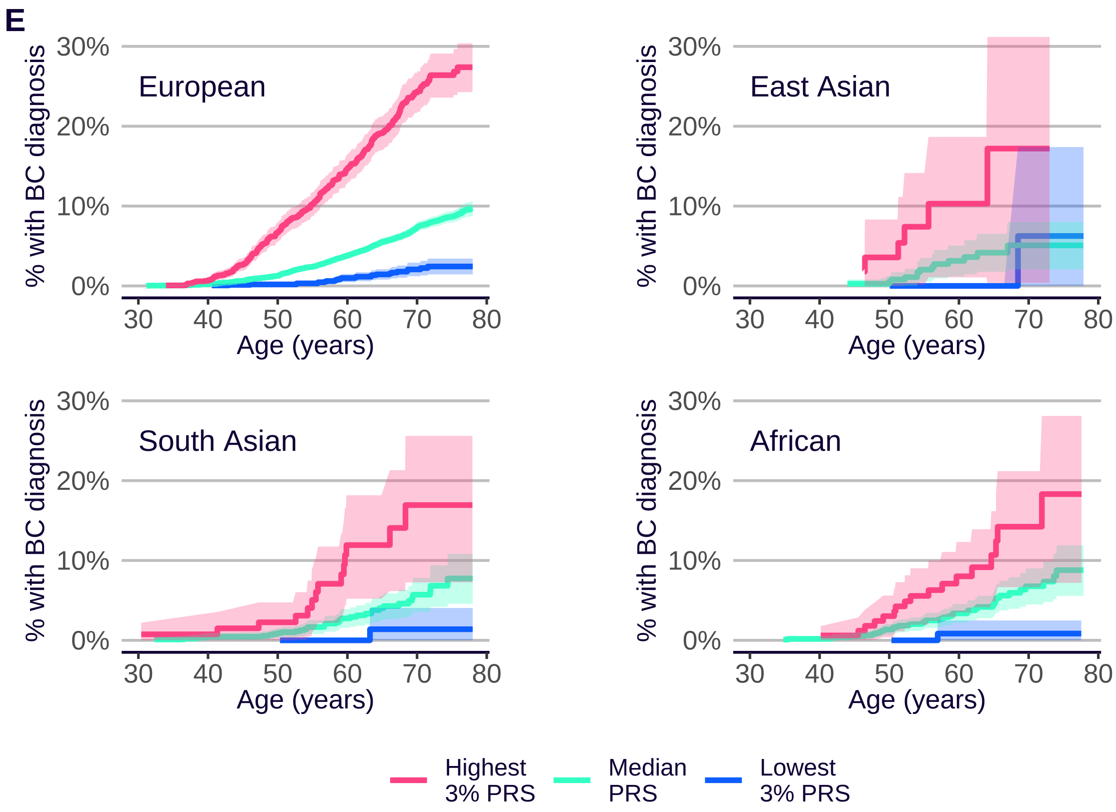
E
European
East Asian
South Asian
African
% with BC diagnosis
0%
10%
20%
30%
30
40
50
60
70
80
Age (years)
Highest 3% PRS
Median PRS
Lowest 3% PRS

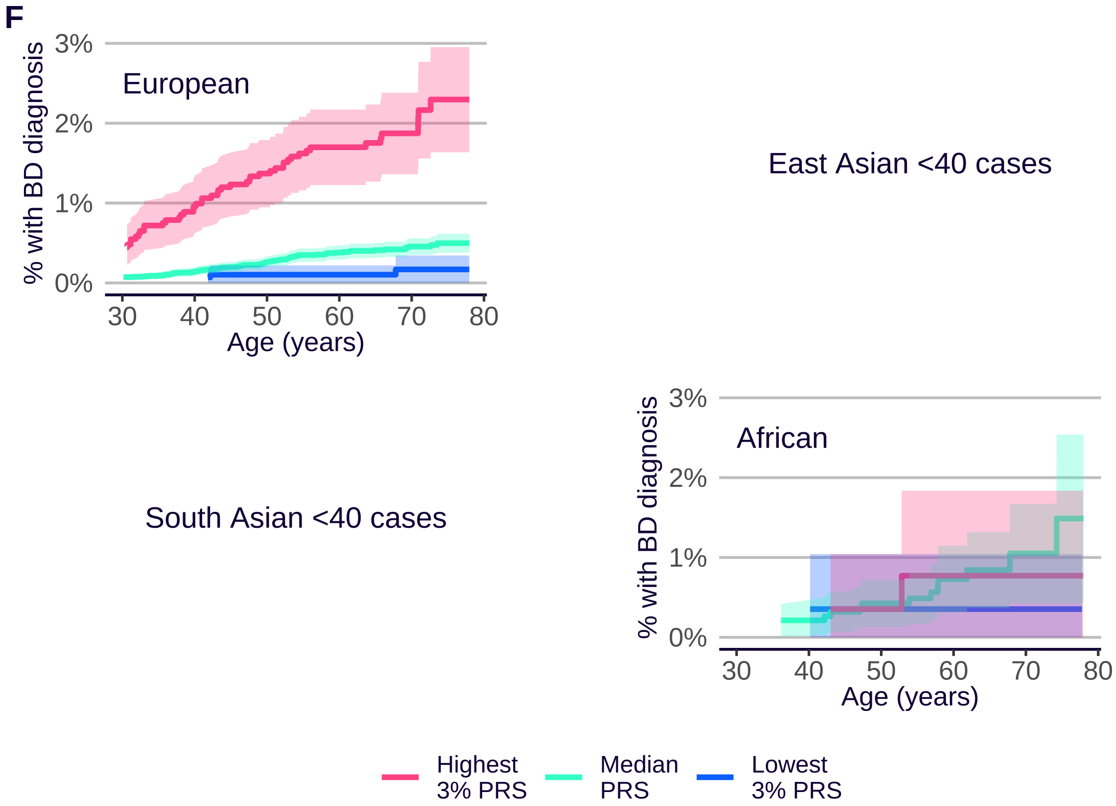
F
European
% with BD diagnosis
3%
2%
1%
0%
30
40
50
60
70
80
Age (years)
East Asian <40 cases
South Asian <40 cases
African
% with BD diagnosis
3%
2%
1%
0%
30
40
50
60
70
80
Age (years)
Highest 3% PRS
Median PRS
Lowest 3% PRS

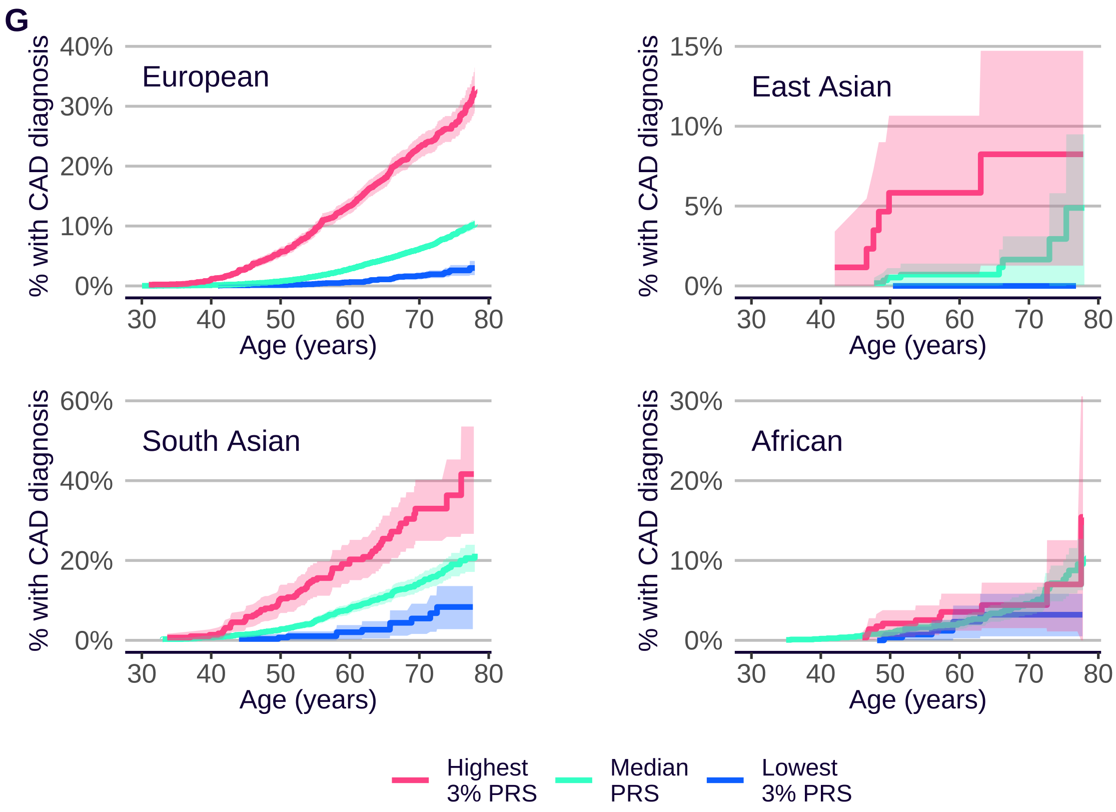

G
European
East Asian
South Asian
African
% with CAD diagnosis
Age (years)
0%
10%
20%
30%
40%
5%
15%
60%
30
40
50
60
70
80
Highest 3% PRS
Median PRS
Lowest 3% PRS

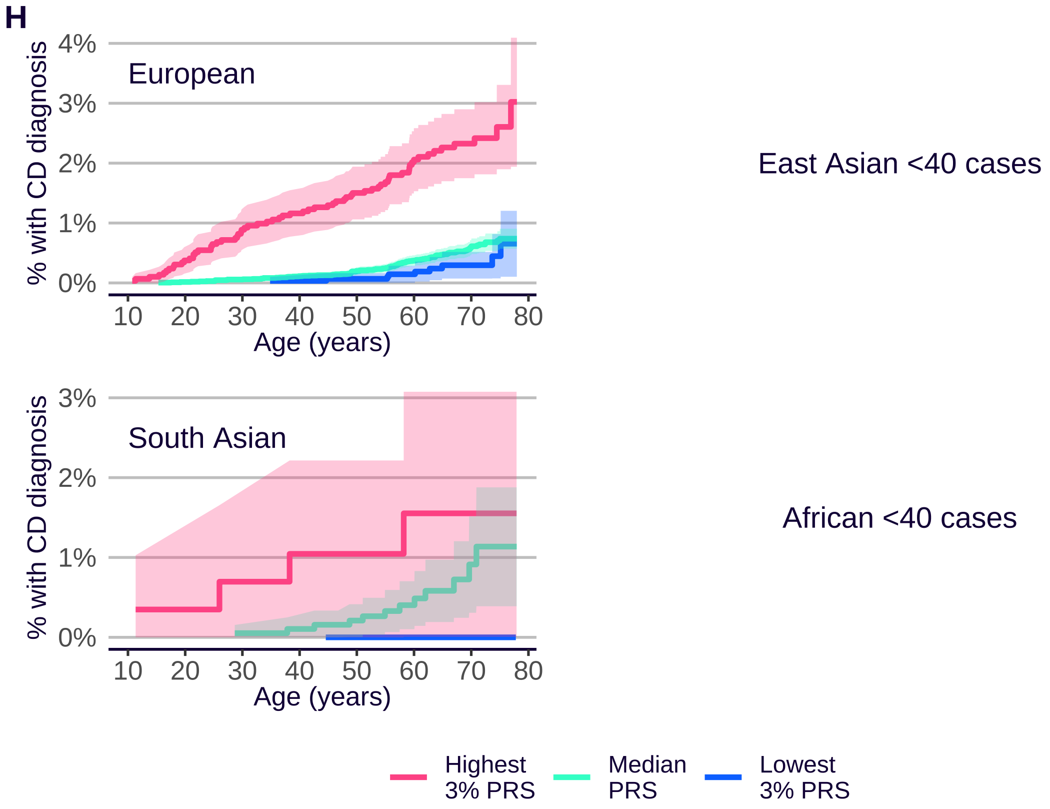
H
% with CD diagnosis
4%
3%
2%
1%
0%
European
10
20
30
40
50
60
70
80
Age (years)
East Asian <40 cases
% with CD diagnosis
3%
2%
1%
0%
South Asian
10
20
30
40
50
60
70
80
Age (years)
African <40 cases
Highest
3% PRS
Median
PRS
Lowest
3% PRS

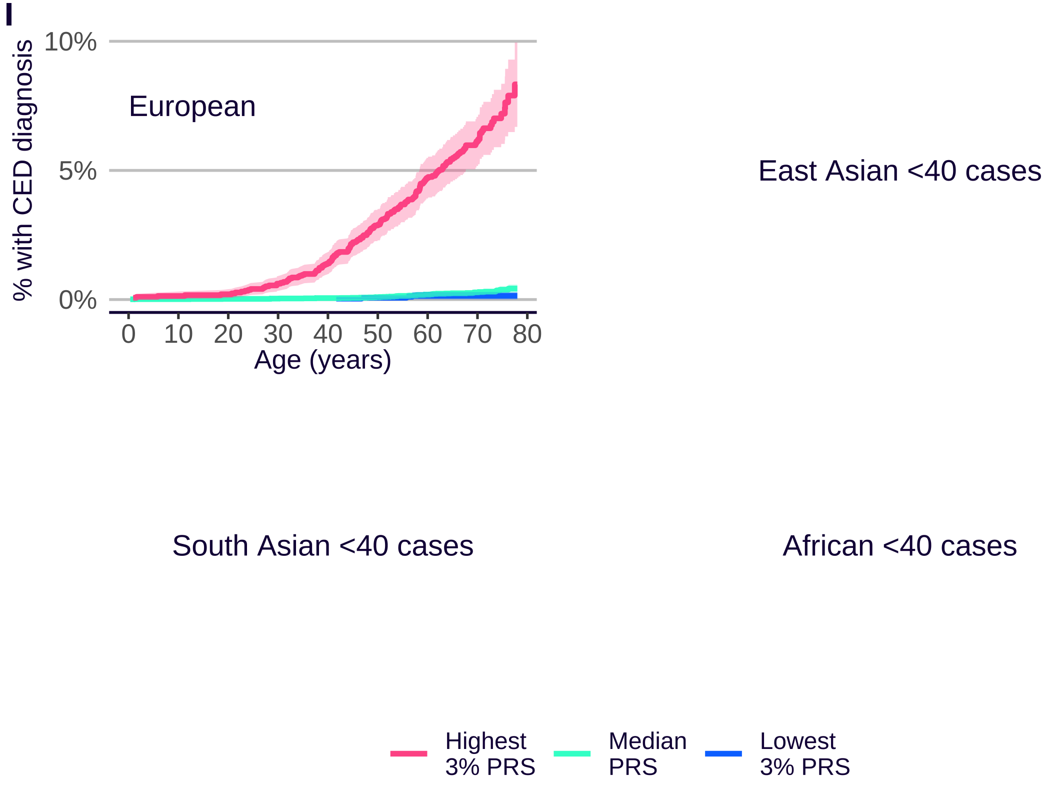
I
% with CED diagnosis
10%
5%
0%
European
0
10
20
30
40
50
60
70
80
Age (years)
East Asian <40 cases
South Asian <40 cases
African <40 cases
Highest 3% PRS
Median PRS
Lowest 3% PRS

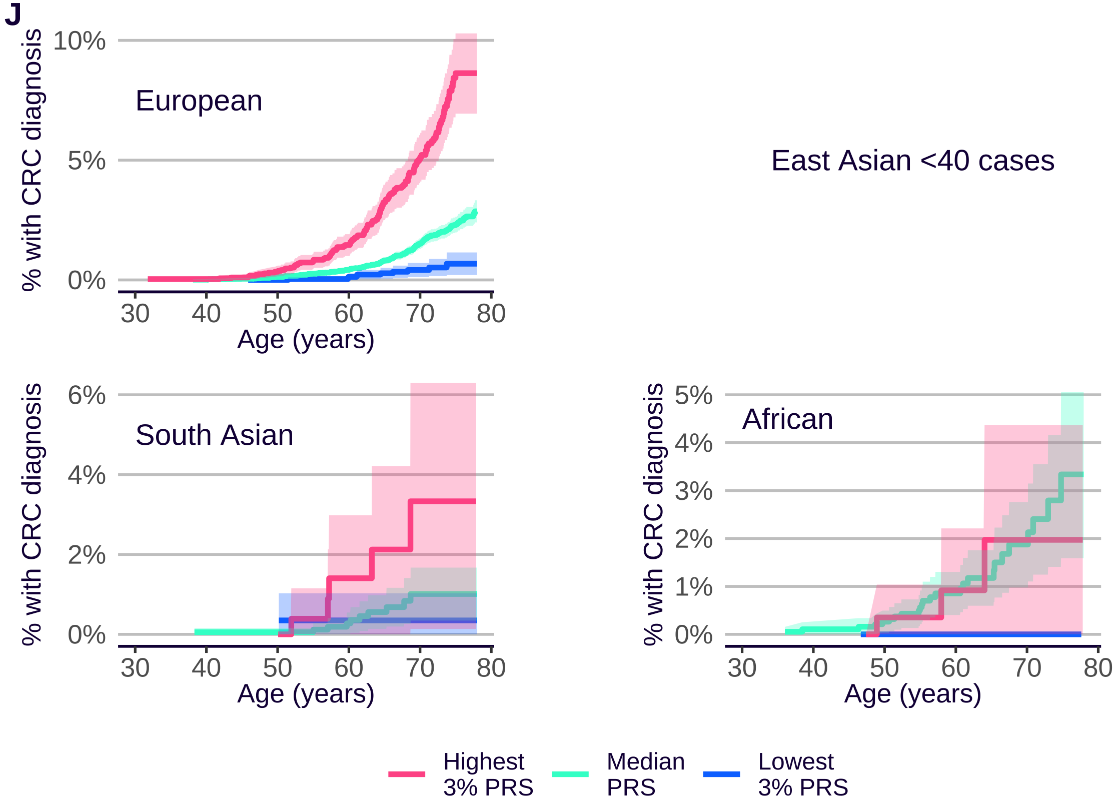
J
European
% with CRC diagnosis
10%
5%
0%
30
40
50
60
70
80
Age (years)
East Asian <40 cases
South Asian
6%
4%
2%
0%
African
5%
4%
3%
2%
1%
0%
Highest
3% PRS
Median
PRS
Lowest
3% PRS

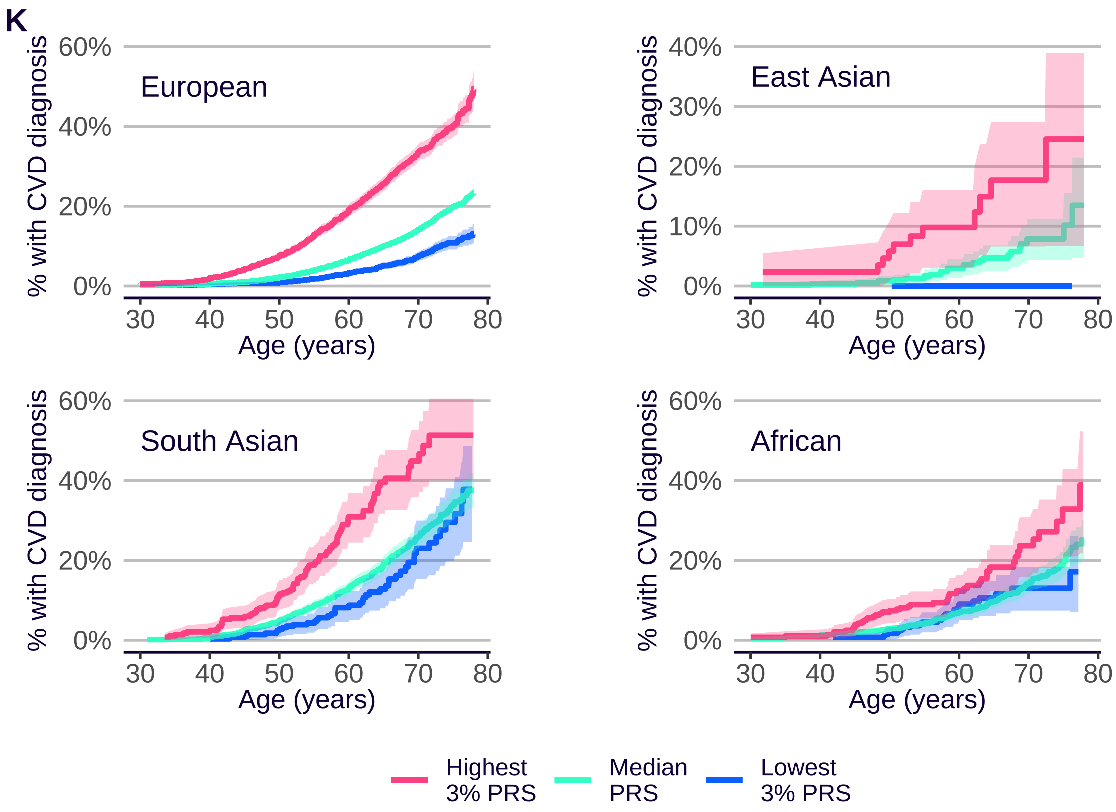
K
European
East Asian
South Asian
African
% with CVD diagnosis
Age (years)
0%
10%
20%
30%
40%
60%
30
40
50
60
70
80
Highest 3% PRS
Median PRS
Lowest 3% PRS

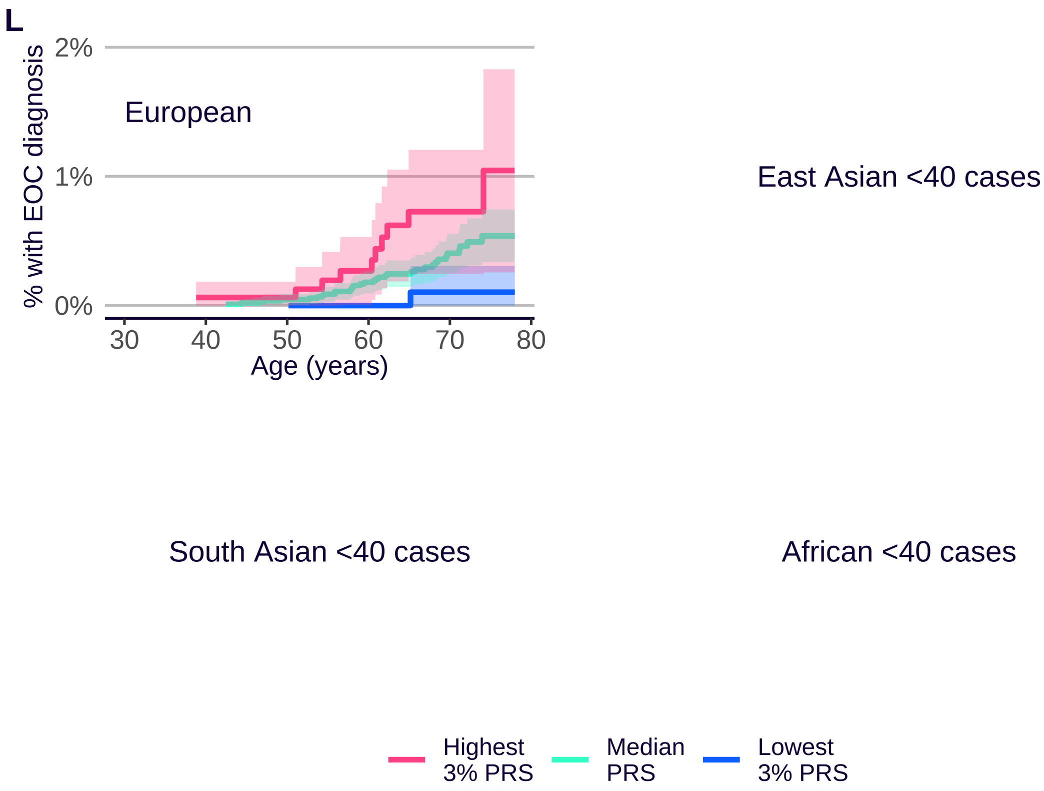
L
European
% with EOC diagnosis
2%
1%
0%
30
40
50
60
70
80
Age (years)
East Asian <40 cases
South Asian <40 cases
African <40 cases
Highest 3% PRS
Median PRS
Lowest 3% PRS

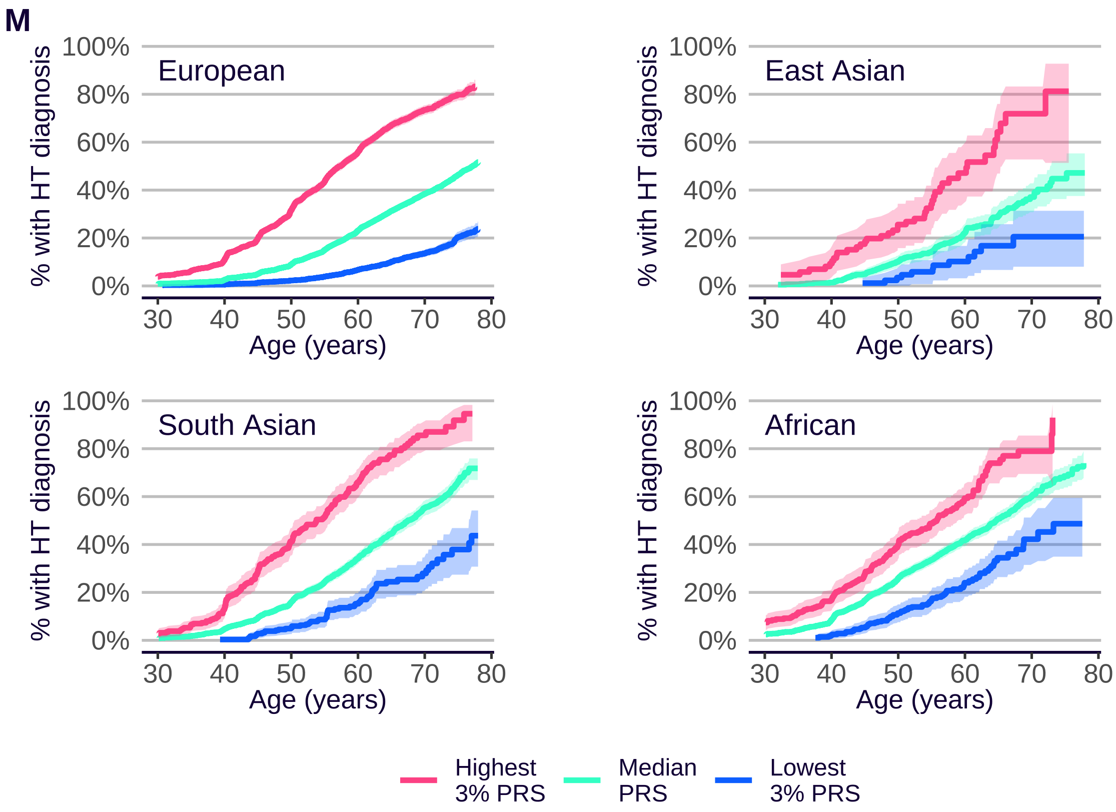
M
European
East Asian
South Asian
African
% with HT diagnosis
0%
20%
40%
60%
80%
100%
30
40
50
60
70
80
Age (years)
Highest 3% PRS
Median PRS
Lowest 3% PRS

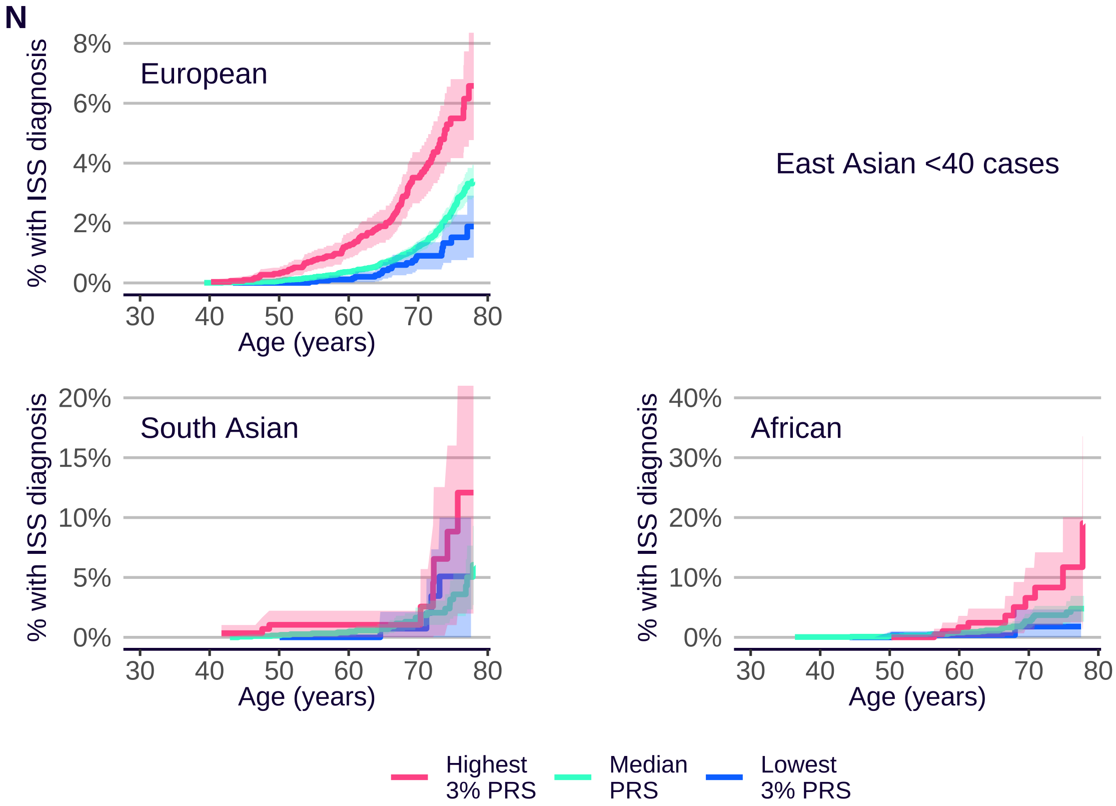
N
European
East Asian <40 cases
South Asian
African
% with ISS diagnosis
Age (years)
8%
6%
4%
2%
0%
20%
15%
10%
5%
40%
30%
30
40
50
60
70
80
Highest
3% PRS
Median
PRS
Lowest
3% PRS

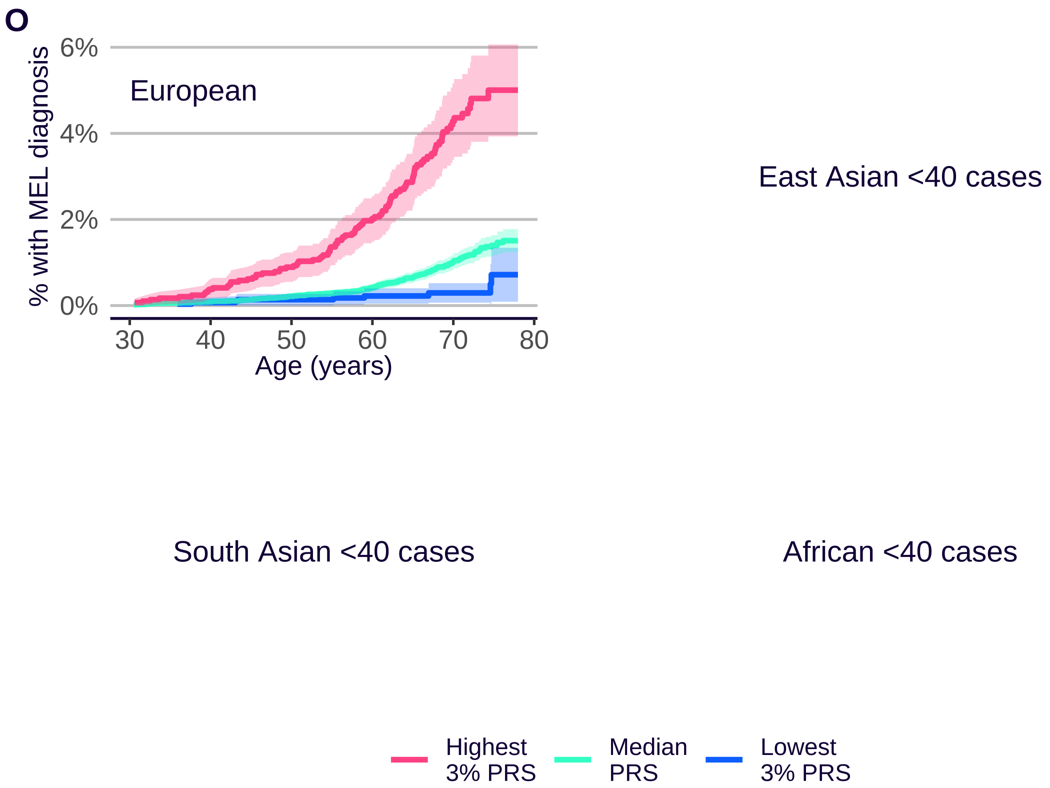
O
European
% with MEL diagnosis
6%
4%
2%
0%
30
40
50
60
70
80
Age (years)
East Asian <40 cases
South Asian <40 cases
African <40 cases
Highest 3% PRS
Median PRS
Lowest 3% PRS

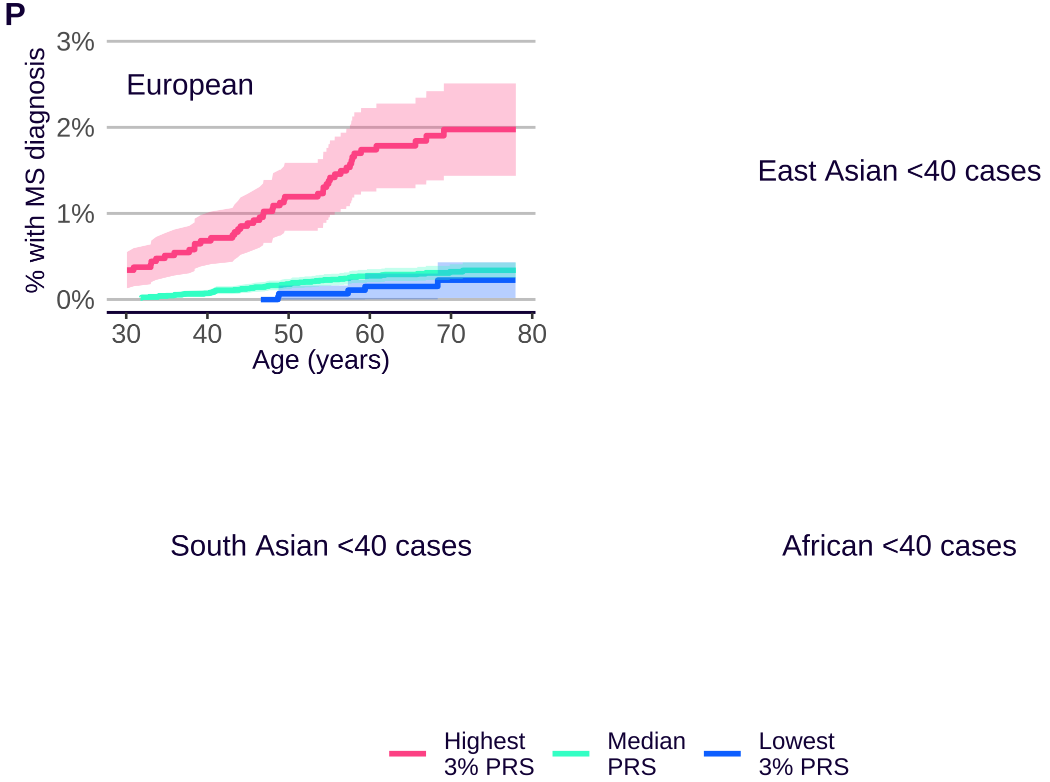
P
% with MS diagnosis
3%
2%
1%
0%
European
30
40
50
60
70
80
Age (years)
East Asian <40 cases
South Asian <40 cases
African <40 cases
Highest 3% PRS
Median PRS
Lowest 3% PRS

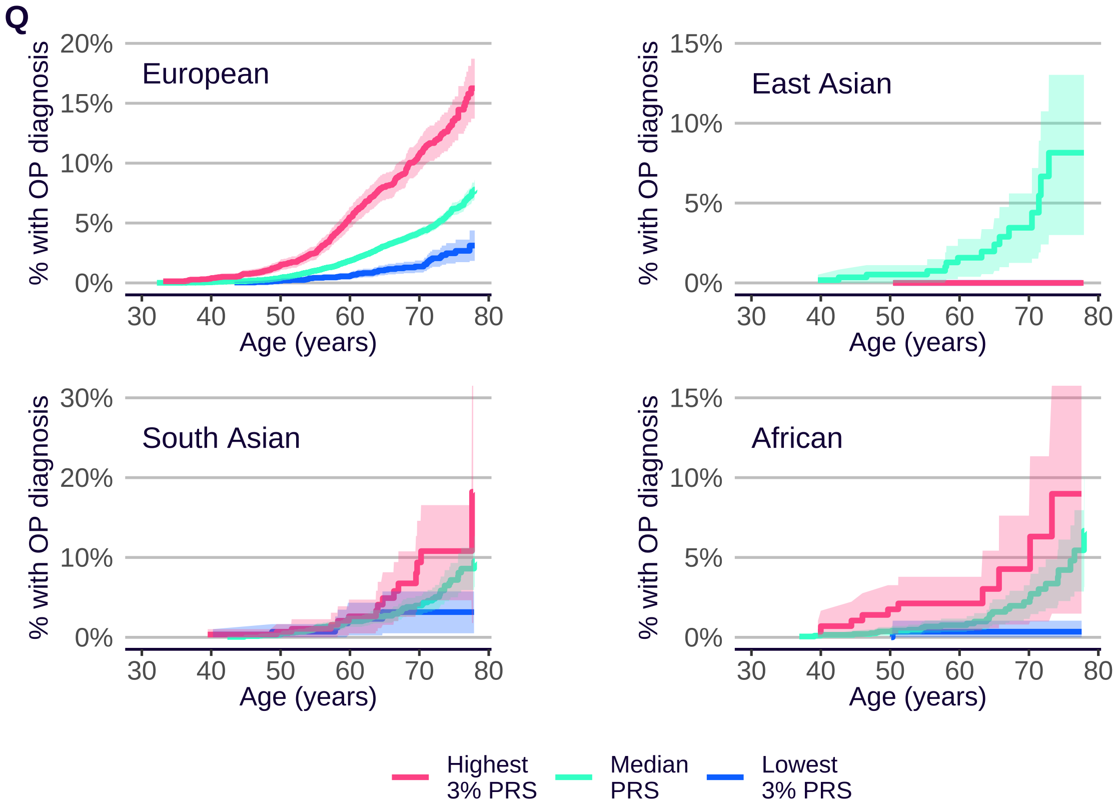
Q
European
East Asian
South Asian
African
% with OP diagnosis
Age (years)
0%
5%
10%
15%
20%
30%
30
40
50
60
70
80
Highest 3% PRS
Median PRS
Lowest 3% PRS

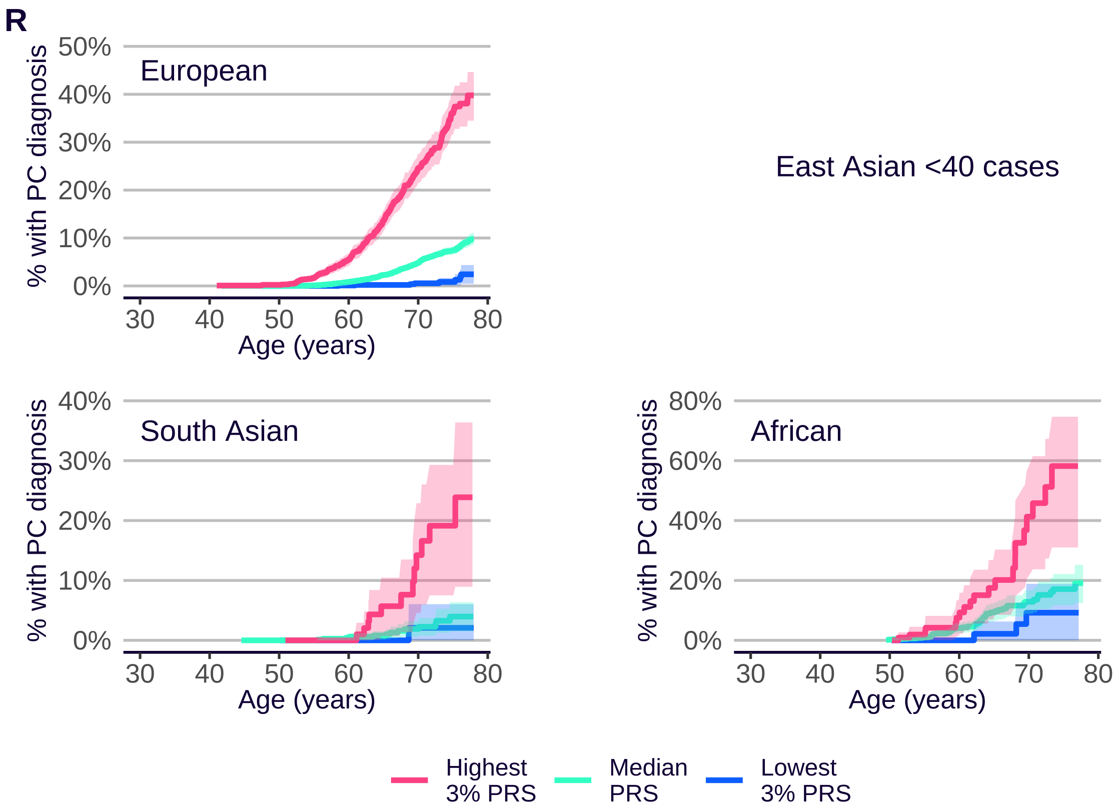
R
European
East Asian <40 cases
South Asian
African
% with PC diagnosis
Age (years)
0%
10%
20%
30%
40%
50%
60%
80%
30
40
50
60
70
80
Highest 3% PRS
Median PRS
Lowest 3% PRS

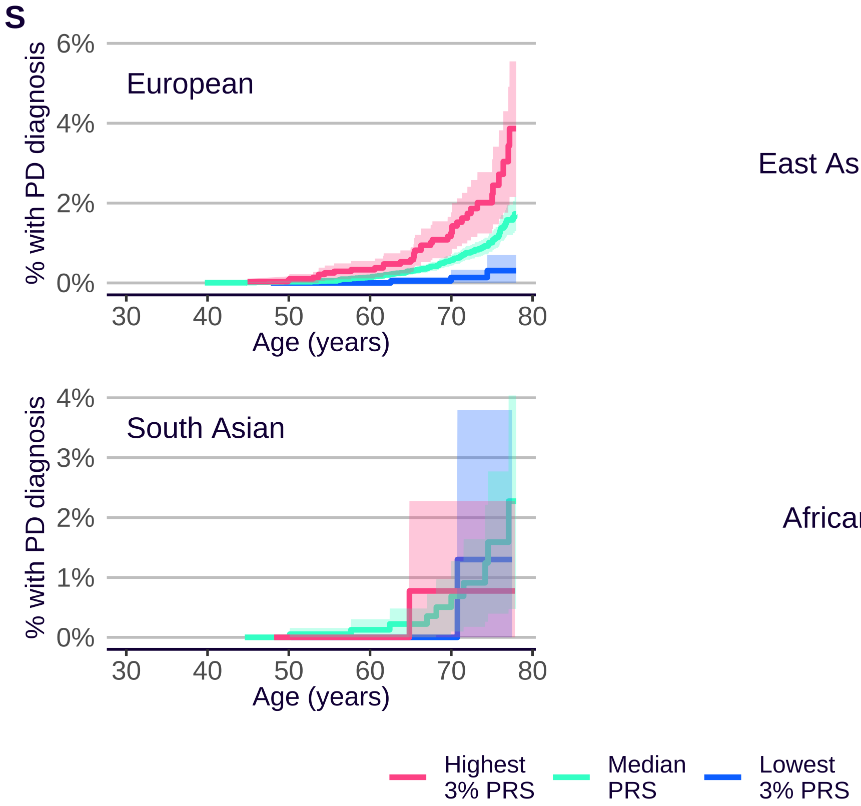

East Asian <40 cases

African <40 cases

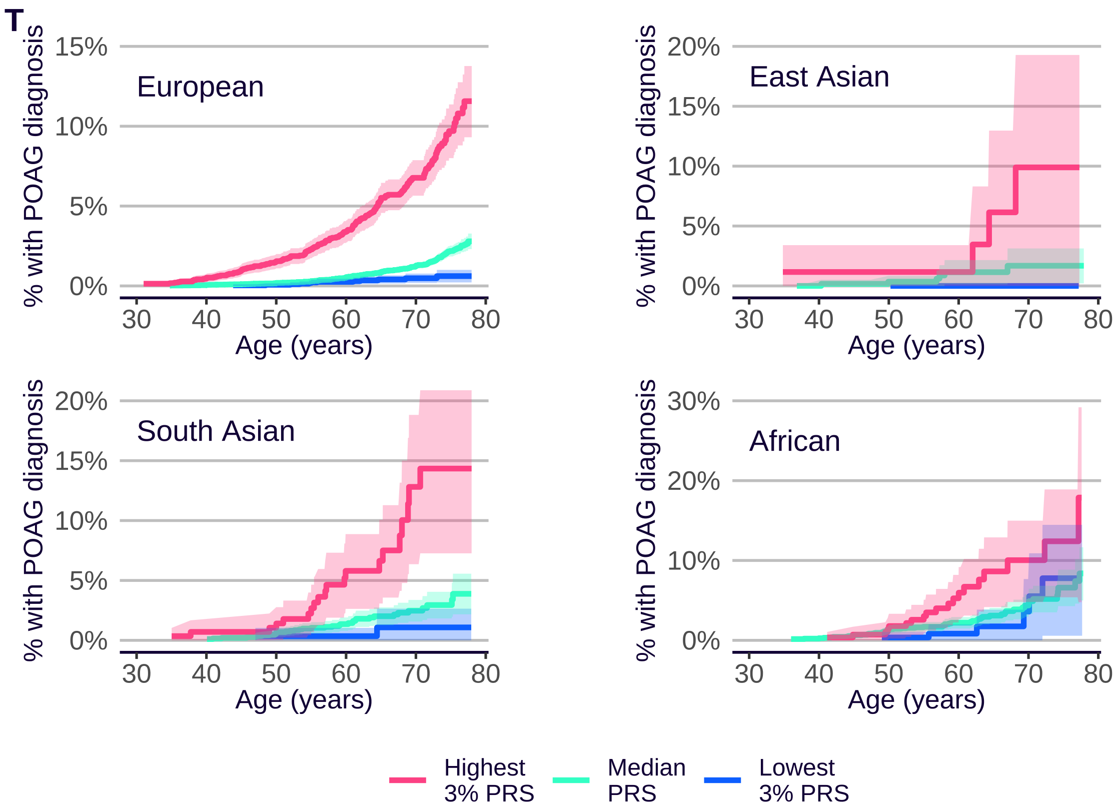
T
European
East Asian
South Asian
African
% with POAG diagnosis
Age (years)
0%
5%
10%
15%
20%
30%
30
40
50
60
70
80
Highest
3% PRS
Median
PRS
Lowest
3% PRS

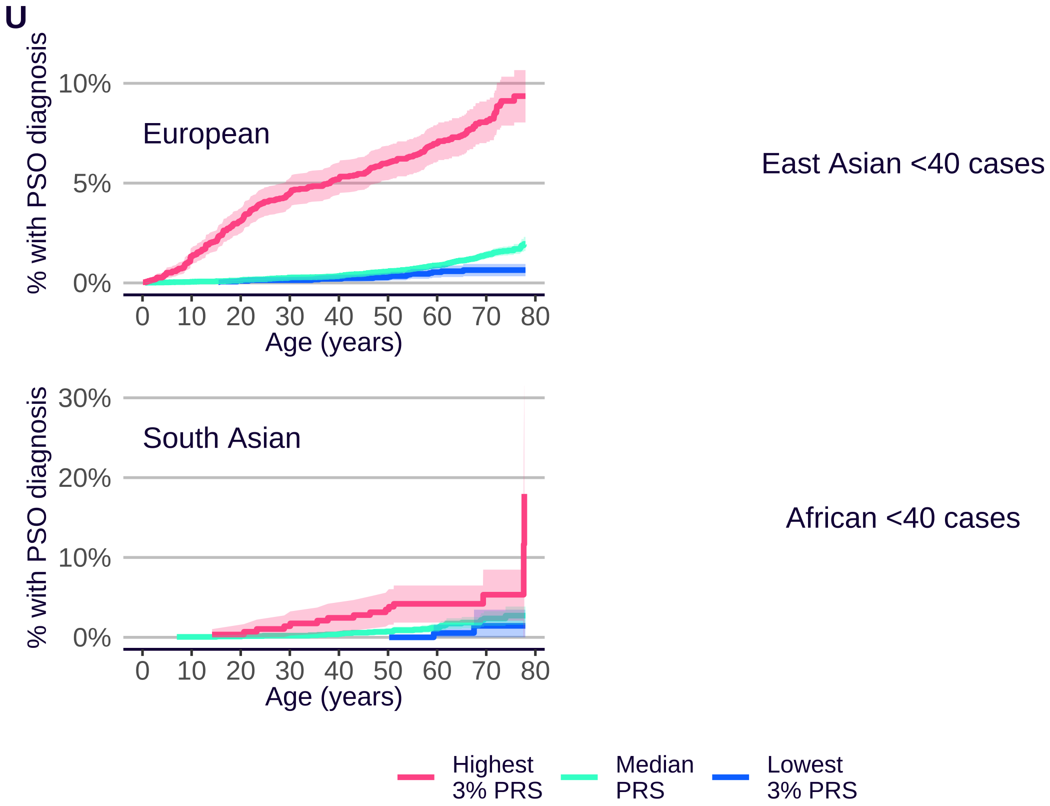
U
European
% with PSO diagnosis
10%
5%
0%
0
10
20
30
40
50
60
70
80
Age (years)
East Asian <40 cases
South Asian
% with PSO diagnosis
30%
20%
10%
0%
0
10
20
30
40
50
60
70
80
Age (years)
African <40 cases
Highest 3% PRS
Median PRS
Lowest 3% PRS

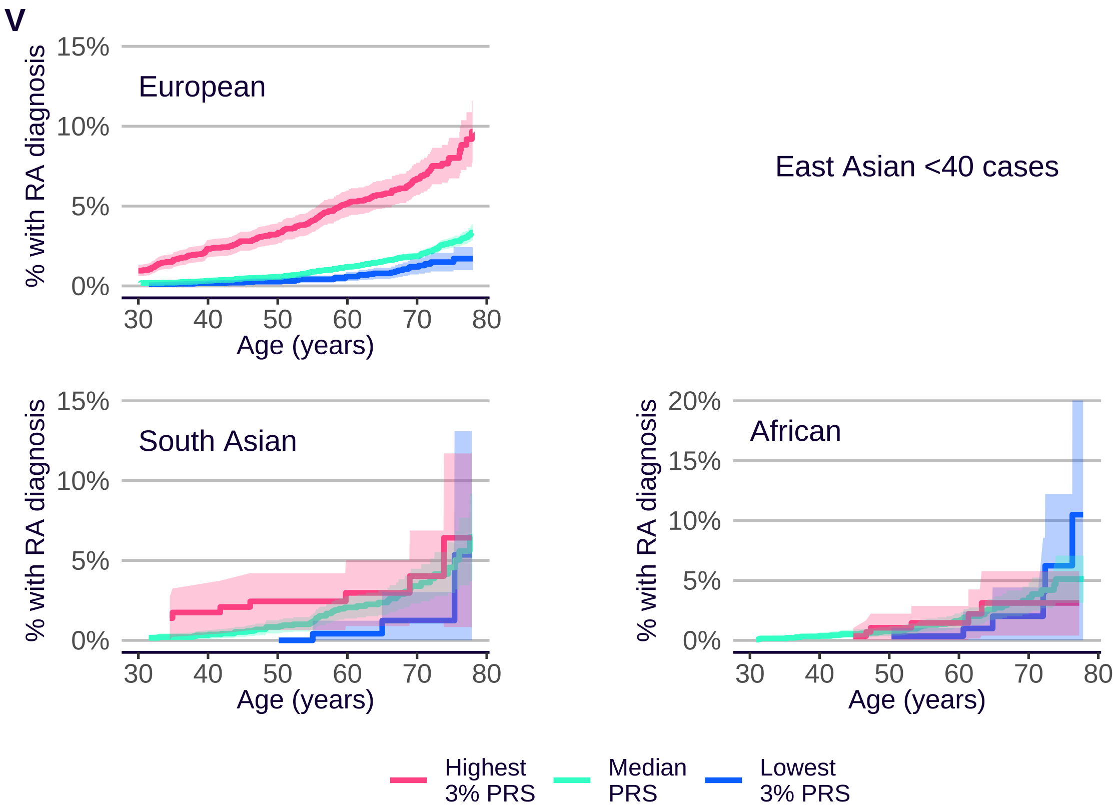
V
European
East Asian <40 cases
South Asian
African
% with RA diagnosis
Age (years)
0%
5%
10%
15%
20%
30
40
50
60
70
80
Highest 3% PRS
Median PRS
Lowest 3% PRS

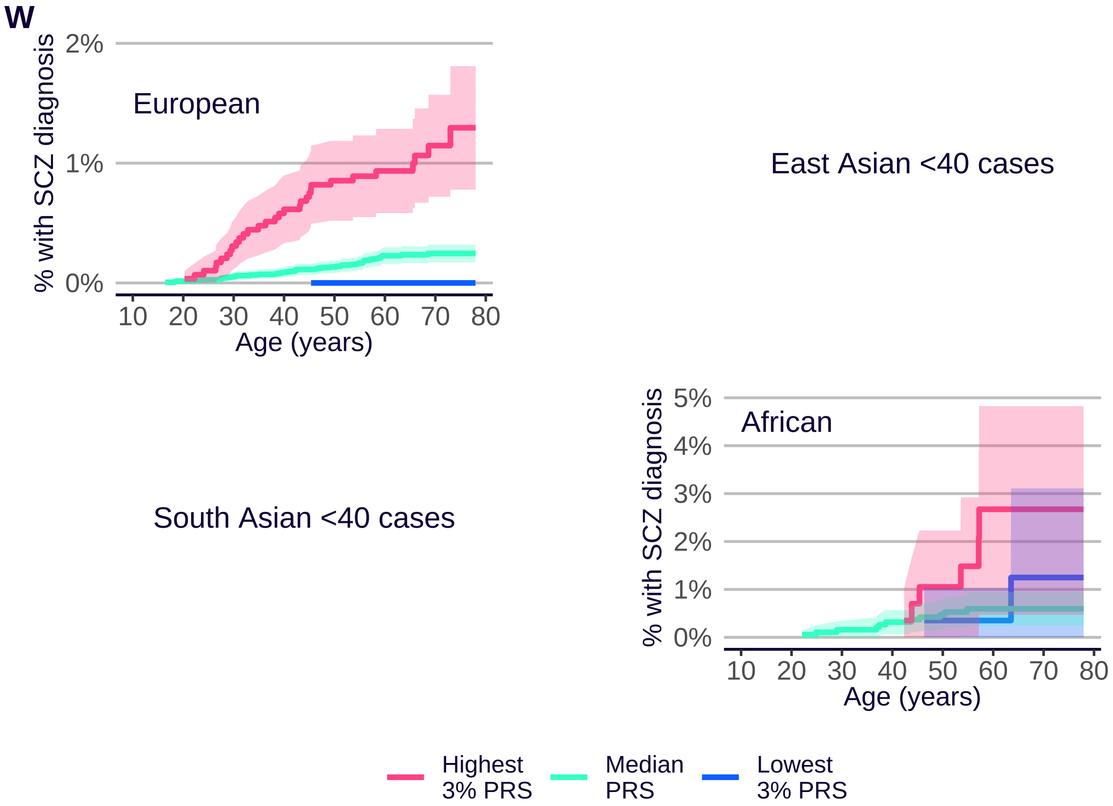

W
European
% with SCZ diagnosis
2%
1%
0%
10
20
30
40
50
60
70
80
Age (years)
East Asian <40 cases
South Asian <40 cases
African
5%
4%
3%
2%
1%
0%
Highest 3% PRS
Median PRS
Lowest 3% PRS

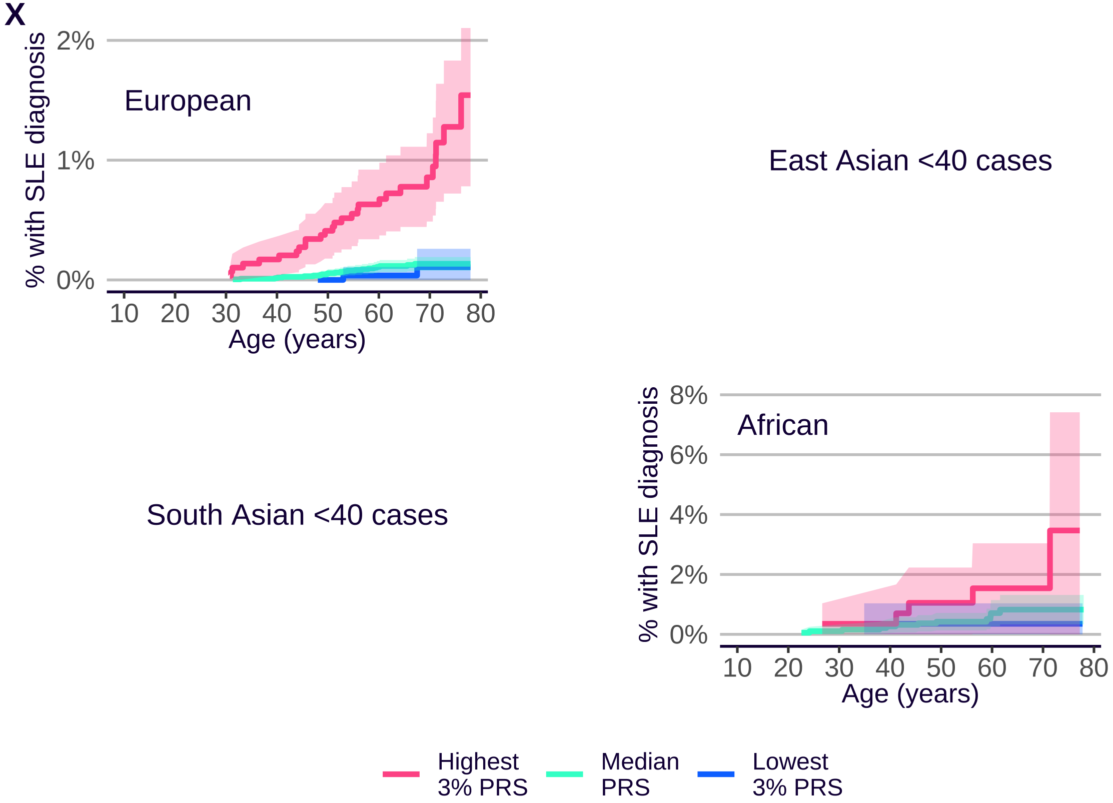
X
% with SLE diagnosis
2%
1%
0%
European
10 20 30 40 50 60 70 80
Age (years)
East Asian <40 cases
South Asian <40 cases
8%
6%
4%
2%
0%
African
% with SLE diagnosis
10 20 30 40 50 60 70 80
Age (years)
Highest 3% PRS
Median PRS
Lowest 3% PRS

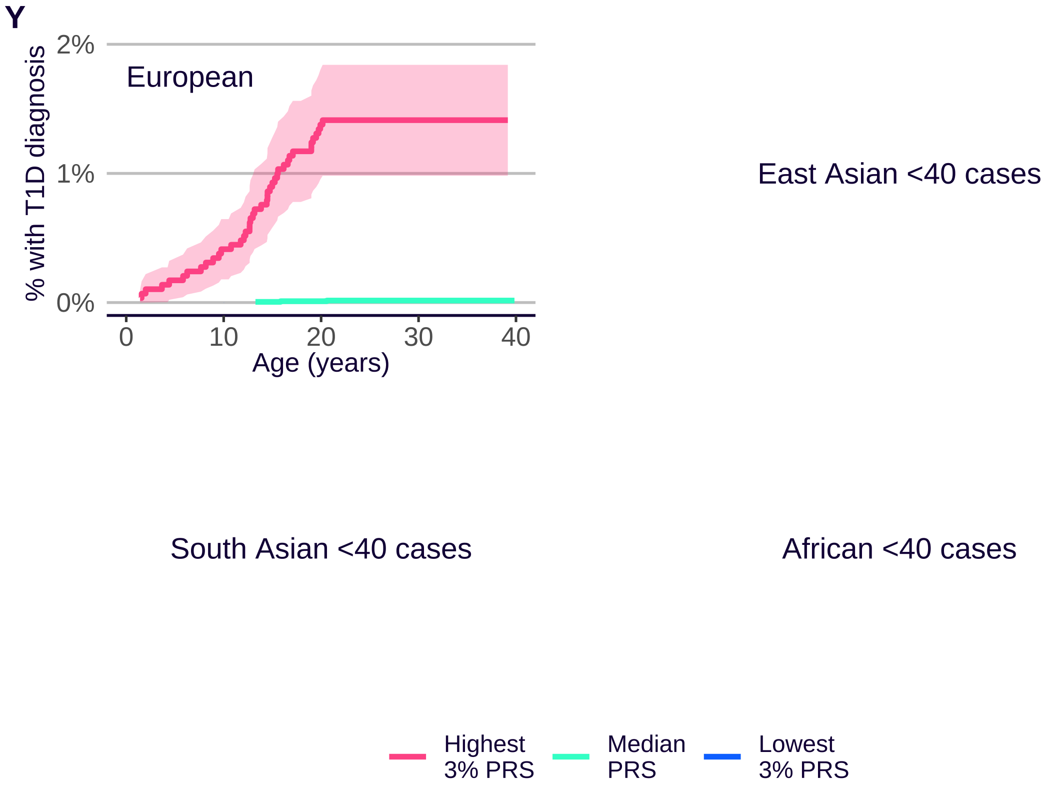
Y
2%
1%
0%
% with T1D diagnosis
European
0
10
20
30
40
Age (years)
East Asian <40 cases
South Asian <40 cases
African <40 cases
Highest
3% PRS
Median
PRS
Lowest
3% PRS

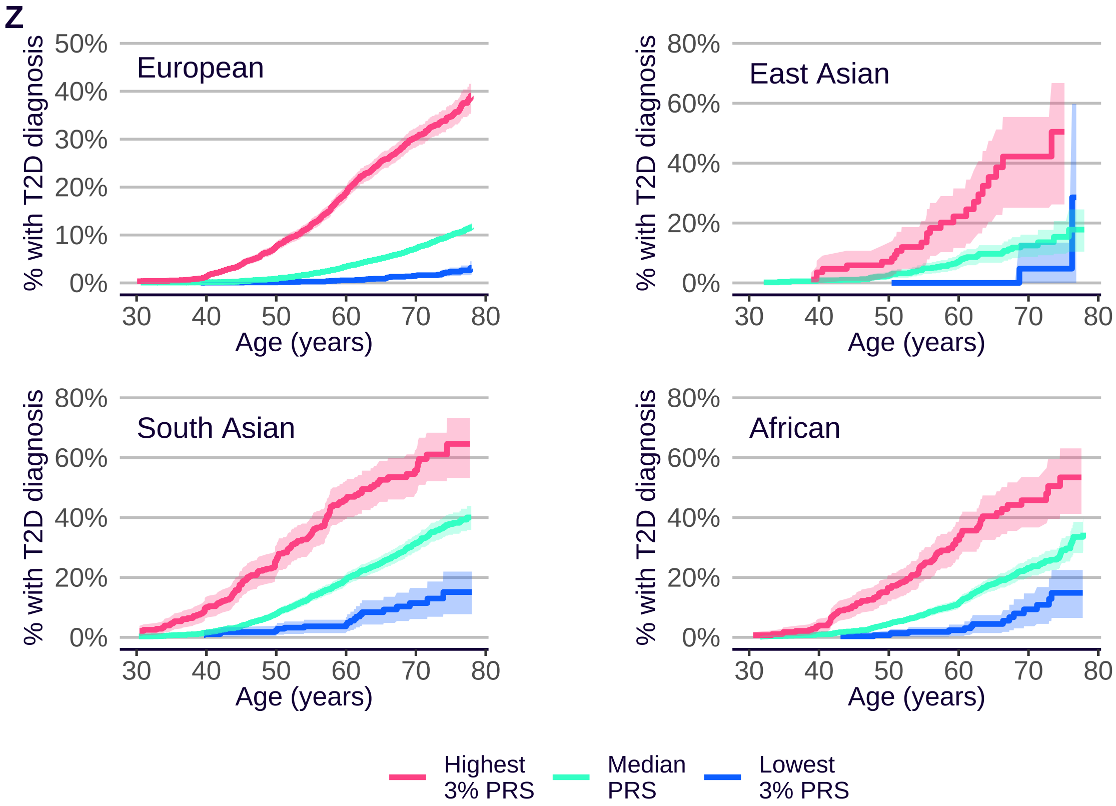
Z
European
East Asian
South Asian
African
% with T2D diagnosis
Age (years)
0%
10%
20%
30%
40%
50%
60%
80%
30
40
50
60
70
80
Highest
3% PRS
Median
PRS
Lowest
3% PRS

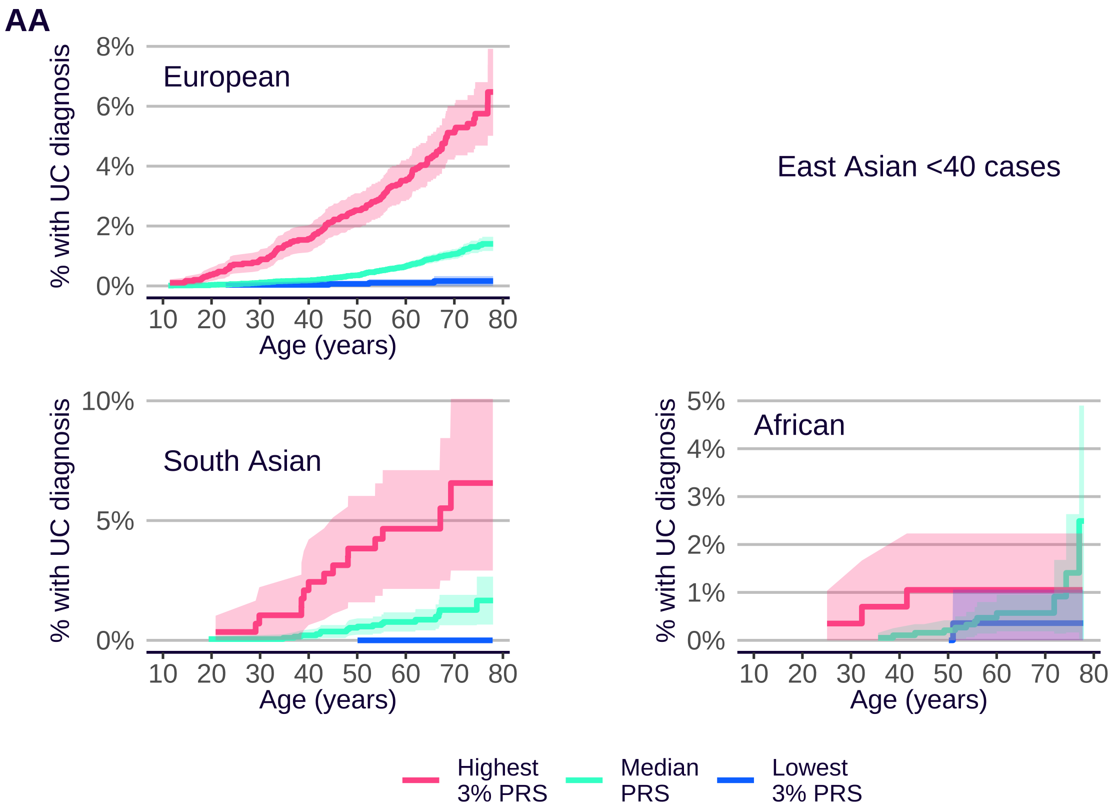
AA
European
% with UC diagnosis
8%
6%
4%
2%
0%
10 20 30 40 50 60 70 80
Age (years)
East Asian <40 cases
South Asian
10%
5%
0%
African
5%
4%
3%
2%
1%
0%
Highest 3% PRS
Median PRS
Lowest 3% PRS

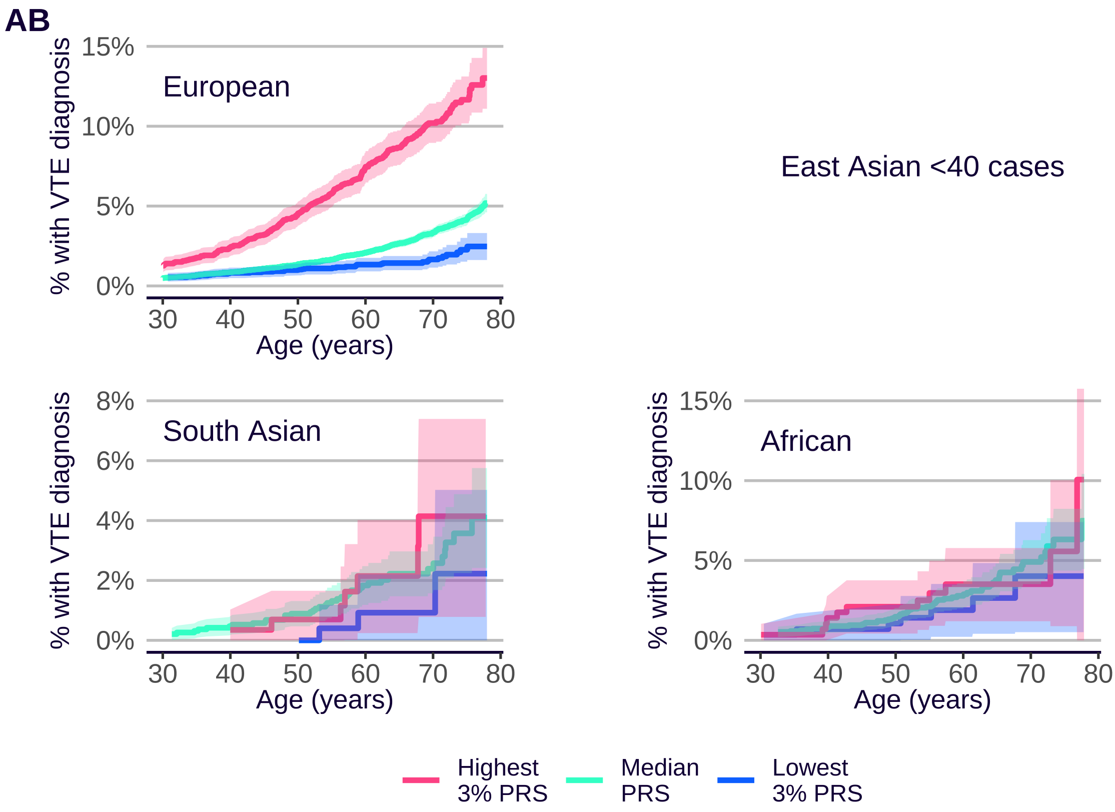
AB
European
East Asian <40 cases
South Asian
African
% with VTE diagnosis
15%
10%
5%
0%
8%
6%
4%
2%
30
40
50
60
70
80
Age (years)
Highest
3% PRS
Median
PRS
Lowest
3% PRS
